# Equity in HIV/AIDS services requires optimization of mainstreaming sectors in Ethiopia

**DOI:** 10.1186/s12889-024-19016-5

**Published:** 2024-06-01

**Authors:** Aklilu Endalamaw, Charles F. Gilks, Fentie Ambaw, Yibeltal Assefa

**Affiliations:** 1 School of Public Health, The University of Queensland, Brisbane, Australia; 2 College of Medicine and Health Sciences, Bahir Dar University, Bahir Dar, Ethiopia; 3 School of Public Health, College of Medicine and Health Sciences, Bahir Dar University, Bahir Dar, Ethiopia

**Keywords:** Challenges, Equity, HIV/AIDS, HIV/AIDS services, Mainstreaming, Ethiopia

## Abstract

**Background:**

Mainstreaming HIV and AIDS across sectors is crucial to close the disparities in service provision and coverage. However, evidence has shown that certain social groups are left behind in receiving HIV/AIDS services. The objective of this study was twofold: to understand the reasons behind the existing inequities and to explore challenges of equity in HIV/AIDS services in the Amhara region of Ethiopia.

**Methods:**

Twenty-two adults (aged 26–57 years) from eighteen sectors that are mainstreaming HIV and AIDS were purposefully selected until the point of saturation and participated in a semi-structured in-depth interview conducted between January 20 and February 17, 2023. Interviewees were asked to describe their mainstreaming experiences in equitable HIV/AIDS services, reflect on the challenges and barriers that impede equitable service provision, or explain the reasons behind the existence of inequity in HIV/AIDS services. The interviews were audio recorded, transcribed, translated, and iteratively analysed, with early analysis informing subsequent interviews. An inductive-reflexive thematic analysis was conducted, whereby themes and subthemes were identified, and the relationships between subthemes and patterns were critically reviewed.

**Results:**

The challenges to equitable HIV/AIDS service provision were grouped into eight thematic areas: (1) changing contexts that shifts public and government attention to emerging diseases, war and political instability, and poverty; (2) leadership-related, such as the lack of supervision and monitoring, not politicising HIV/AIDS (not providing political attention to HIV/AIDS) and weak intersectoral collaboration; (3) financial constraints due to a random budgeting and contract interruption with non-governmental organisations (NGOs); (4) lack of resources due to scarcity and unfair distribution; (5) inadequate skilled personnel due to inadequate numbers and lack of continuous professional and career development; (6) lack of equity-related evidence-based tools and guidelines; (7) inadequate understanding of equity due to lack of training and misunderstanding, and lack of access to equity-oriented tools and guidelines; and (8) cultural norms, values, and perceptions.

**Conclusions:**

This study identified critical challenges faced in the equitable HIV/AIDS services provision. To achieve equity in HIV/AIDS services, mainstreaming sectors need to invest in mechanisms to sustain services in emergency situations; identify effective leaders to maintain collaboration, monitoring, and evaluation; institutionalise responsive budgeting and establish alternative funds to maintain non-governmental organisations initiatives; provide continuous up-to-date training and create a common evidence-sharing platform; implement proper recruitment, education, and professional development of HIV/AIDS focal persons; and promote and practice culturally safe care. It is, therefore, essential to optimise sectors that are mainstreaming HIV/AIDS and incorporate equity considerations in their strategic plans and working guidelines.

**Supplementary Information:**

The online version contains supplementary material available at 10.1186/s12889-024-19016-5.

## Introduction

Mainstreaming human immunodeficiency virus (HIV) and acquired immunodeficiency syndrome (AIDS) refers to the process through which government and non-governmental sectors change the organisational policies and practices in order to effectively respond to the HIV/AIDS epidemic [1]. Sectors that are mainstreaming HIV and AIDS are engaging in infection prevention services to reach community members in need through internal and external mainstreaming [2]. Various sectors, which work for education, transport, health, food security, social services, employment, and administration, have been mainstreaming HIV/AIDS services [3, 4]. Prior to the initiation of this initiative, Ethiopia endorsed the National Policy on HIV/AIDS Control Programme in 1998 [5]. The strategy promoted the provision of HIV/AIDS services with due emphasis to human rights. In 2004, Ethiopia understood the importance of a Multisectoral National Strategy [6]. This strategy underlined the implementation of HIV/AIDS sector mainstreaming to limit the spread of the epidemic. Additionally, in 2011, the National HIV/AIDS Mainstreaming Implementation Strategy was established, identifying a focus on ‘gender equity and equality’, and vulnerable groups within the sector (e.g., peer education at various levels within the organisation) and outside the sector, aiming for customers and their families [7]. Nationally and globally, several equity-based initiatives besides mainstreaming HIV/AIDS have been implemented [8].

Equity is a critical fast-track driver for achieving the sustainable development goals related to HIV/AIDS services in different countries. Globally, equity has been taken as one of the principles in the universal ART provision since 2003 [9]. It has also been reported that the UN member states have agreed on the importance of prioritising equity in order to expand essential health care, including HIV/AIDS services, to everyone everywhere by 2030 [10]. The overarching goal is to reduce new HIV infections to below 200 thousand globally [11]. Achieving this target necessitates substantial efforts to prevent 1.3 million new infections, based on a baseline of 1.5 million new HIV infections in 2020 [12]. This further requires every country to have an effective HIV/AIDS programme [13]. Similarly, Ethiopia plans to reduce HIV/AIDS inequality by 2025 through the implementation of sustainable mainstreaming services, aimed at decreasing new HIV infections to less than 1 per 10 thousand people and achieve the three 95s by 2030 [14]. According to the 2022 Joint United Nations Programme on HIV/AIDS (UNAIDS) report, there were about 8,300 (ranging between 4, 000 and 18,000) new HIV infections recorded among all ages in Ethiopia [15]. It is crucial to continue efforts to provide equitable HIV prevention services.

Despite several strategies, inequities in HIV/AIDS persist. When socioeconomic health equity (also known as health equity) is not achieved, it contributes to this form of health inequity [16]. Health equity is ‘the absence of unfair and avoidable or remediable differences in health among population groups defined socially, economically, demographically or geographically’ [17]. HIV/AIDS prevalence varies across different social classes in Ethiopia. For instance, women were more likely to be infected by HIV compared to men [18]. The disparity in HIV infections may indeed be due to inequities in HIV/AIDS services across social classes and other structural factors.

Inequities in HIV/AIDS services persist in many countries. To illustrate, individuals facing social disadvantages (e.g., those from rural areas, lower household wealth rank, and lower education status) often encounter barriers in accessing HIV/AIDS services in Latin American and Caribbean countries, Malawi, and South Africa [19–23]. Similarly, in Ethiopia, inequity remains a challenge, even being identified as a challenge of the unsuccessful millennium development goals [24]. Others have also revealed socioeconomic inequality in knowledge about HIV/AIDS, as well as stigma and discrimination or accepting attitudes towards people living with HIV, and HIV testing practice in Ethiopia [25–28]. These studies primarily focused on inequities related to individual or social characteristics. In addition, there was no clear evidence on why inequities in HIV/AIDS services persisted. However, it is equally important to examine the challenges stemming from structural factors to achieve equity in service provision [29–31].

Structural factors encompasses social determinants of the health process, policies and norms that influence health outcomes [32]. Notably, social injustices cause systematic differences among different social groups [32]. Additionally, effective leadership, proper health financing, an adequate workforce, and equitable supplies contribute to equity in health or health care [33]. It is suggested that ‘ending the HIV epidemic for all’ needs investigating social structural determinants [34]. Consequently, understanding what and how parts of the social process cause inequity in HIV/AIDS services is a crucial step in setting up strategic interventions. Qualitative research in Uganda in mainstreaming sectors linked challenges to the role and sustainability of donors, responsibility ambiguity, and capacity development [1]. By examining the experiences of sectors that are mainstreaming HIV and AIDS towards equitable service delivery, valuable insights can be gained.

Therefore, this study aimed to explore the challenges of achieving equity in HIV/AIDS services in the Amhara region of Ethiopia. The research question focused on understanding the persistence of inequities in HIV/AIDS-related services, aimed at addressing why inequities in HIV/AIDS services persisted across different social classes. The findings from this research will identify gaps in utilising mainstreaming sectors for equitable service provision. Additionally, it will provide valuable insights for practitioners, policymakers, and implementers, enabling them to make progress towards the goals of ‘ending inequality’ by 2025 and ‘ending the global threat of HIV/AIDS’ by 2030. Furthermore, this study will serve as a foundation for future research in this area.

## Methods and materials

### Study design and setting

This study is a cross-sectional qualitative study to understand challenges or barriers that caused or widened inequity in HIV/AIDS service in the Amhara region of Ethiopia [35, 36]. Ethiopia has an estimated population of nearly 115 million people as of 2020, with 78.1% residing in rural area [37]. The first HIV from stored serum samples and clinically overt AIDS case were detected in 1984 and 1986, respectively [38]. Ethiopia declared AIDS epidemic in 2000 [39], and established councils across various sectors at the national level to prevent and control the expedited dissemination of HIV [39]. Antiretroviral therapy provision began in 2003, and free ART became available since 2005 [40]. In 2004, the first Multisectoral National Strategic Plan was established [6], followed by a second Multisectoral Strategic Plan for 2010 to 2015 [41]. These strategic plans are adapted and utilized by each region of the country. Ethiopia comprises 10 regions and two administration cities, one of which is the Amhara region where HIV infection is observed. Between 2019 and 2021, out of 3,129 individuals who underwent HIV testing in some parts of Amhara region, 14.2% tested positive for HIV [42]. Based on wealth quantile in 2016, the distribution in the Amhara region was as follows: 16.4% poorest, 21.0% poorer, 22.7% middle, 22.9% richer, and 17.0% richest. Additionally, based on education status, 52.3% of women versus 43.4% men were non-educated [43].

In-depth interviews were conducted with 22 key informants working in HIV/AIDS regional-level mainstreaming sectors. The main regional-level strategic sectors and civil society organisations, based on information from the Amhara Regional Health Bureau multisectoral office, were purposefully selected. The main regional strategic sectors have many employees within the sectors and many project sites within the community through which the health system reaches a large segment of the community with HIV/AIDS services. The regional-level sectors which participated in the current study are presented in Table 1.

**Table 1:** Sectors that are mainstreaming HIV and AIDS involved in the study and number of key informants in each regional level Bureaus

Regional level Bureaus	Number of key informants
Health Bureau	3
Industry and Investment Bureau	1
Work and Training Bureau	1
Trade and Development Enterprises Agency Bureau	1
Construction and Urban Development Bureau	1
Education Bureau	1
Agriculture Bureau	1
Labour and Social Affairs Bureau	1
Women, Children, and Youth Affairs Bureau	1
Civil service commission Bureau	1
Youths and Sport Bureau	1
Planning and Development Office	1
Amhara Development Association	1
Transport and logistic Authority Bureau	1
Road Construction and Transport Bureau	1
Bahir Dar University	2
Network of HIV Positive and Development Organisation	2
Amhara Mass Media Agency	1

### Recruitment

Recruitment for the key informants in sectors that are mainstreaming HIV and AIDS was conducted in collaboration with the Amhara Region Health Bureau multisector action and policy office, sector managers, and peer information. The participants were purposefully selected. First, the authors intentionally identified potential sectors and created lists of potential interviewees. However, when the pre-identified interviewees were not available, AE asked them to replace another potential interviewee. Then, they assisted AE by phone call to establish smooth communication with the next interviewee. Afterward, AE met the new interviewee and introduced the overall research processes and purposes. Author A.E. conducted semi-structured interviews between January 20 and February 17, 2023. Interviews explored key informants’ experiences with barriers to the provision of equitable HIV/AIDS services. After completing the one-on-one interview with the first key informant in each institution, another interview with the second interviewee was conducted on the same or another day. Recruitment was continued until the point of saturation was achieved, which is the time when no new information is explored by adding additional participants [44].

### Data collection tools and procedures

Author AE prepared a draft interview guide, and co-authors reviewed the written document. They conducted several meetings to enhance the interview guide. When disagreement arose regarding any of the questions, the authors referenced the study objective and added their comments based on their expertise and priori experiences. The guide was prepared with open-ended questions. Subsequently, the interview guide was shared with each interviewee before the interview date to identify potential doubts or questions. Comments and concerns were clarified and addressed prior to conducting the interview. The interview guide and reflection sheet are presented in the supplementary file (Supplementary file-1).

The interview guide, supplemented by probing techniques, lasted an average of 33 min. Structural determinants involved a semi-structured interview that incorporated structural contexts. These contexts are social determinants processes that allowed participants, through probing, to explore more on how these contexts barred equity in services [31]. Participants were asked to reflect on their role and responsibility in HIV/AIDS service provision, equitable service provision strategies that they are approaching, challenges that widened or caused inequity, and suggestions to overcome challenges or barriers. An audio recorder was used to record the audio data. The interview was conducted in the interviewee’s office, or a suitable place based on the participant’s choice. Reflective notes were taken during interview time.

### Data quality control

Prior to collecting data, participants were provided with an information sheet and agreed to participate in the study with informed consent details. The information sheet and consent form contained detailed information. The participant information sheet explained the research topic and its purpose, the principal investigator and supervisors’ roles, the reason why sectors that are mainstreaming HIV and AIDS are selected for this study, data collection method for interviewing about their experiences and challenges to equity in HIV/AIDS-related services using audio-recorder, the maximum time to stay with the interview (maximum one hour), data anonymisation by removing personal identifiers, and telling them that the recordings will only be used for research purposes, recorded audio will be kept in secured office and electronic data will be password protected. Participants were also informed that only the principal investigator and the supervisors have the right to use the recorded audio, telling them that they will not get any benefit or take any risk by participating in the current research and confirming their right to refuse to be study participants, stop an interview, or refuse any question whenever they feel uncomfortable or want to stop it. Furthermore, participants have received the email and phone contacts of all authors and the University of Queensland Research Institute Review Board in Australia and Bahir Dar University College of Medicine and Health Sciences Ethical Review Board in Ethiopia for any reason related to the current research. Then, participants asked questions and got responses from the principal investigator to fully understand the purpose and the overall data collection process.

Then, the interview guide was given to them in person and/or electronically to improve and/or clarify the interview questions. The recording device was checked prior to interview time for its functionality and stored in a safe manner. The interviewee’s statements were summarised at the end of each interview, and they were asked to confirm the accuracy of the summarised points. The interviewees confirmed all their discussion points at the end of the interview by summarising their main points. Additionally, through repeated listening to the recorded audio and data collected in the interviewees’ first language (Amharic), A.E became familiar with the audio, transcribed it, and carefully translated the transcribed content into English. Then, the transcribed and translated texts were sent to interviewees via email communication for their approval. Again, additional comments were incorporated and sent back to participants for their confirmation. Those who didn’t respond to the email were assumed to have confirmed the translation and transcription of the content.

Furthermore, in a regular weekly meeting, the authors also discussed and agreed on the interpretation of codes, concepts, and themes that ensured the rigour and trustworthiness of the findings. AE conducted the interviews, listened to the daily audio records, rehearsed them, transcribed the audio, translated the Amharic version to English, and performed all the analysis. In each step, AE prepared the first version and YA reviewed it. When disagreement arose, AE took responsibility for checking the developed codes, interrelated concepts, and themes. During each meeting, the agenda from previous meeting was reviewed, and agreements were reached before proceeding with the current meeting. The credibility of this research approach was ensured because the principal investigator had sufficient interaction time, took a qualitative research method course, participated in qualitative data-analysis trainings, and took NVivo 12 software training for qualitative data analysis. The other authors are experts in qualitative analysis.

### Data analysis

**Fig. 1:**
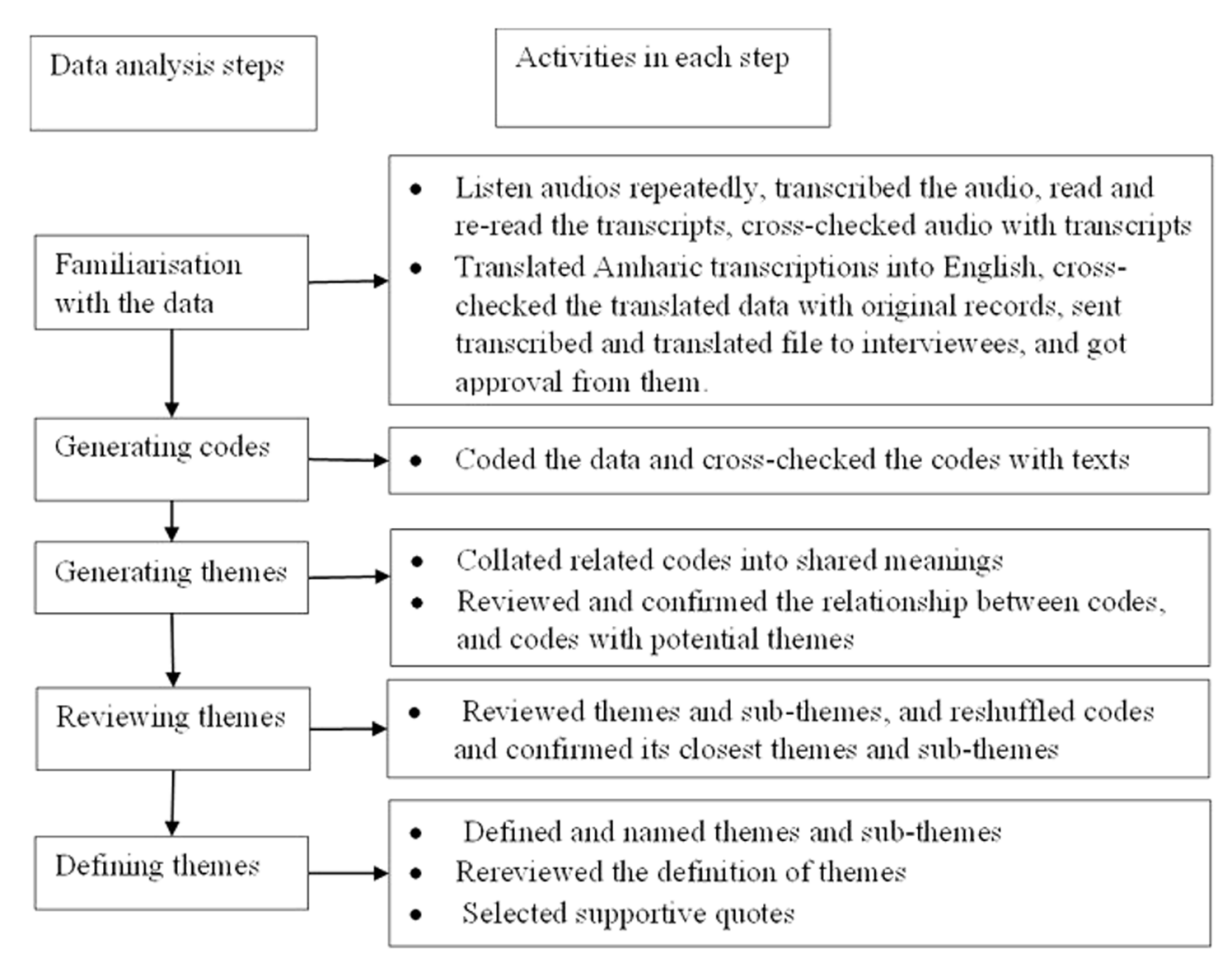
Schematic representation of data analysis steps

Inductive-reflexive thematic analysis was used to identify, analyse, and report recurring themes within the text [45, 46]. The authors utilised the WHO’s health system building blocks and social process structure to probe during the interviews and create themes during the analysis, supplementing the inductive analysis [47]. Once the interviews were completed, verbatim transcription was performed. The matching of transcribed content with records was done. The authors read the transcript repeatedly to get an understanding on it. Author A.E is fluent in Amharic as first language for the transcription into English (Supplementary file-2). The analysis was conducted using NVivo 12 software, in which thematic maps, coding, re-categorising, and description were performed [48]. FA provided a guide on how to conduct qualitative analysis and create code/theme, in addition to AE’s weekly meetings with YA. Author AE prepared the first draft of the code and/or themes, which YA reviewed at each step and discussed during weekly meetings. Disagreements were resolved by checking the audio records, transcribed, and translated files. Additionally, AE reviewed available literature on social determinants of health when recategorization of codes or themes was needed. CFG also reviewed the first and subsequent version of the results. Author AE repeatedly checked the coding and themes and made comparisons with the translated and transcribed texts, and brought discussion points to the team when facing challenges in coding and theme creation. Discussions solved any differences or disagreements in coding and themes. Texts were organised according to challenges that barred equity or aggravated inequity in HIV/AIDS services. Appropriate quotations were extracted to support the thematic analysis. The overall data analysis approached a stepwise process (Fig. 1).

### Ethical considerations

The University of Queensland Human Research Ethics Committee provided ethical approval of this research (Project number: 2022/HE001751). Additionally, the College of Medicine and Health Sciences, Bahir Dar University Human Research Ethics Committee in Ethiopia offered ethical approval. Then, written permission was secured for Amhara Public Health Institute. Finally, the Amhara Public Health Institute wrote a supporting letter to the Amhara Region Health Bureau and sectors that are mainstreaming HIV/AIDS. Participants were asked for informed consent. The names and other identification characteristics of participants are kept confidential. The data is used only for study purposes.

## Results

### Participant characteristics

Participants were aged between 26 and 57 years, with a mean age of 42 years. Twelve participants were female and ten were male. Participants had experience ranging from 1 to 15 years, with an average of 6 years and 8 months as an HIV/AIDS expert or focal person. Regarding the education status of participants, 11 had completed second-degree education (master’s degree), ten had completed first-degree education (bachelor’s degree), and one had completed diploma education.

### Overview of themes

The challenges of equity in HIV/AIDS services are revealed through major themes. These are generally structural problems, such as context change with its demands (emerging diseases: corona virus 2019 (COVID-19), hypertension, diabetes, cancer; and war or instability), resource shortage (scarcity and unfair distribution), ineffective leadership (absence of monitoring and supervision, weak intersectoral collaboration), human resource challenges (inadequate personnel and lack of recognition), budget problems (inappropriate budgeting, interruptions in support from non-governmental organisations (NGOs), high out-of-pocket costs), lack of updated equity-based tools and guidelines, inadequate understanding of equity, and cultural norms, values, and perception.

### Theme one: contextual changes with emerging demands

Sectors that are mainstreaming HIV and AIDS exist to close the gaps in services between social categories. However, the changing health, economic, and political contexts shift leaders’, communities’, and societies’ attention to install and invest resources to the current situation. Subsequently, a stagnant HIV/AIDS equity-based policy strategy, health financing problems, and leadership instability in prioritising agendas, budgeting, and implementing HIV/AIDS services challenged the system. Emerging diseases were one of the most cited barriers to the provision of equitable HIV/AIDS services as part of changing contexts. Participants responded that they were focused on COVID-19 prevention activities, though their main role is dedicated to HIV prevention services. Additionally, an increasing burden of chronic diseases (cancer, diabetes, and hypertension) diverts people and leaders from giving emphasis to HIV/AIDS issues. Participants’ direct quotes are as follows:

“These two years of COVID have slowed things down. We are investing resources for COVID-19 prevention and consequences instead of HIV.” Female, 49 years old.

“Sometimes, even if you offer the service to them, there is no interest, and it has been observed that people underestimate this virus because their attention goes to blood pressure, diabetes, and cancer rather than fearing HIV.” Female, 48 years old.

There was a consensus among participants that war or conflict prevents them from reaching communities in need with services. This not only interrupted their routine activities but also took their attention, budgets, and resources. The direct participants’ discussion is presented below.

“The political instability is preventing us from doing our jobs properly. It is not possible to do usual work, let alone work in special conditions that are inaccessible. The alternation and volatility of political agendas have led to this disease being deprived of attention.” Male, 29 years old.

“The war and the violence affect all sections of society, so we focus on the issues that are going to kill you presently, so no one remembers HIV transmission and services, despite its bad consequences.” Female, 45 years old.

### Theme two: ineffective or lack of leadership roles

Leadership has great importance to create political will for HIV/AIDS, identifying policy strategies, supporting and regulating intersectoral collaboration, and monitoring and supervision. Whatever the several dynamic contexts, mainstreaming sectors explained that leaders expected to continue giving emphasis to equitable HIV/AIDS services unless the disease disappeared or became of non-public health importance. Participants explained that a lack of government and public attention to HIV/AIDS, weak intersectoral collaboration, and the absence of monitoring and supervision widened inequity in HIV/AIDS service coverage.

#### Government and public negligence/lack of attention to HIV/AIDS

Participants consistently cited that government and public negligence or lack of attention cause or widened HIV/AIDS service inequity. They described in their discussion how current issues (war or conflict) and emerging diseases drive negligence both in the government and society. Once the political leaders do not place HIV/AIDS on the public agenda, they are unable to set and evaluate equity-based strategies. Female participants reflected upon their experiences:

“One day I prepared a plan and gave it to my boss. Then, he saw it roughly, smiled, and asked me to reply, whether HIV is our current burning problem in our sector.” Female, 42 years old.

“When I was aware, we used to hear about HIV in the media. The HIV/AIDS logo and the sign were posted everywhere, but now they are not. There is a very big gap; in general, those who have knowledge do not implement it, and those who do not know it ignore and neglect it. We forget it from top to bottom.” Female, 31 years old.

Moreover, participants agreed that ignorance about HIV/AIDS is also rooted in the community. They described how the ignorance of people in the community not only interrupts their work but also influences others who need to use HIV services, increasing inequity as a result. A 26-year-old female HIV/AIDS focal person reflected upon her experience during her public awareness creation activities:

For example, when you go out to post stickers about HIV, some say, ‘Are you out of a job?’ I mean… Many people believe HIV is an old and forgotten disease, so they told me, ‘What’s wrong with you here? What are you doing here?’ “They do not give you an ear to listen about HIV…. When one spoke this, I remember that others who were gathered around were distracted from paying attention to my poster.

#### Weak intersectoral collaboration

The structural problem in sectors that are mainstreaming HIV/AIDS was consistently cited as a challenge. In general, the issue of HIV was multifaceted, but there was a gap in how sectors worked together. Sectors that are mainstreaming HIV and AIDS at the regional level are responsible for arranging, coordinating, and leading the HIV/AIDS focal individuals at the zonal and district levels. However, they lack the well-established structure to implement it from top to bottom or bottom to top with the purpose of closing HIV/AIDS service inequities. This was explained as the absence of communication and/or a communication system between the strategic sectors at the region level and zonal or district sectors. In the absence of a weak collaboration and communication system, especially bottom-line sectors may not be updated with a strategic plan, working guidelines, and procedures and cannot report their gaps to the regional sectors, leaving community members in need continuously starved of services. Female and male participants reflected on challenges:

“I am not well connected to the district. The structure has not gone down or been well chained to the zone and district.” Female, 42 years old.

“The process of having a clear understanding and working together is based on personal friendship, not on a clearly established system.” Male, 41 years old.

#### Absence of monitoring and supervision

Participants also explained the consequences that arose due to weak collaboration as there was no monitoring and supervision, whether the activities were done based on the required procedures, proper use of budget, or how much services reached target populations. Female and male participants elucidated the absence of regular monitoring and supervision.

“On the grounds, I don’t see a strategy of monitoring and supervision of what has been planned and what has been done to reach disadvantaged community members.” Female, 48 years old.

“If we did build an institution, the system will ask us whether we have done it or not. But now, whether I work or not, no one asks me.” Male, 41 Years old.

### Theme three: HIV/AIDS mainstreaming workforce problems

Most sectors that are mainstreaming HIV and AIDS have at least one person who works on HIV/AIDS prevention and control activities in the workplace for employees and customers, and project sites, and coordinate reports from zonal or district activities. However, inadequate personnel, a lack of recognition, and a lack of professional or career development were mentioned as challenges in delivering equitable services.

#### Lack of personnel

Participants described that they have no adequate number of personnel to identify individuals in need, pursue all services, and organise reports.

“We have a structure all the way down to the lower administration, which are zonal and district administrations. We also have additional institutions. But there are no HIV/AIDS focal personnel in all sectors that we are administering. It doesn’t mean we will be equally accessible to everyone at the desired level.” Female, 48 years old.

#### Lack of recognition

HIV/AIDS focal personnel lack recognition in some sectors. They are assigned to a secondary role without having paid for the HIV/AIDS services that they are enrolling in pursuing service expansion. This leads them to not plan and implement activities aimed at controlling HIV and distributing services fairly. Some participants also explained that they had not received career development. They felt that they would be more responsive and responsible to tailored HIV service provision if their career development was considered.

“In most mainstreaming sectors, a person is assigned an extra job just like me. I mean what I do when I’m comfortable, it’s not my main task, so when I focus on other tasks, I forget it. When I request them (she mean regional bureau or senior manager) to give me condoms at some point, if they say no, I leave it and focus on my main duties.” Female, 49 years old.

“But the other thing is that while I was in this position, I was not promoted. I believe that as my term of service increases, I need to be promoted to another career; this can make me do more and better work.” Female, 42 years old.

### Theme four: budget problem

#### Inappropriate budgeting

There has been a 2% annual fund for HIV/AIDS services that each sector allocates from the gross annual budget. However, participants mentioned that budgeting issues create problems in providing equitable HIV/AIDS services. Most participants agreed that the budget for HIV/AIDS service mainstreaming was not enough. Budget allocation seems equitable because 2% is applied to every sector, but practically inequitable. It was arbitrary to allocate 2% of the sector’s budget for HIV/AIDS services without considering the number of employees, structures, and targeted populations. This causes under-budgeting or over-budgeting. The first two pieces of information show underbudgeting while the third describes overbudgeting.

“Money is budgeted as 2% of the sector’s budget, but not enough. For example, our sector has around 400 thousand Ethiopian birr for HIV/AIDS. … since the staff is about two hundred, it was not possible to do every activity of HIV/AIDS services.” Female, 49 years old.

“…in the first place, that 2% allocation, which has no calculation, was called 2% arbitrarily, not a clear way of budget allocation for how much work is to be done, and not a planned financial investment. This 2% budget was ended when I gave training to our employees once.” Female, 48 years old.

The 2% budget was overbudgeted sometimes.

“*A direction that each sector should allocate 2% of the available sectors’ budget is highly unlikely and impossible; the budget will be huge. For example, my sector has more than 1 billion birrs (Ethiopian birr). Think about how you’re going to modify 2% of this budget, and sometimes the rules should be made in a way to be practised. This is the part that is bothering us. So, we agreed that just do the work we want to do, and then we will check and balance the finances later.”* Male, 42 years old.

#### Non-contracting with non-governmental organisations

The existing finance and local funds could not cover the costs previously expedited by non-governmental organisations. In the past, many NGOs had been involved in building capacity and reaching selected geographic areas and populations. Participants described how the involvement of NGOs has declined and caused financial stress. Additionally, those organisations offered incentives used to pay higher per diem for trainers and employees, distributed diagrammatic brochures, and had an attractive work environment.

“In the past, there were many partner organisations, so various trainings were carried out. But now, it is decreasing from time to time due to NGOs phasing out.” Male, 54 years old.

“As you know, when NGOs give training, the person who was part of the training has received money. But now, staffs totally do not volunteer to take training without per diem.” Female, 40 years old.

“There are bad lessons from non-governmental organisations. They used to pay allowances or per diem when they gave training, today they are gone. When we say we are going to train, it is a person who asks you for an allowance or per diem. Because he is used to those organisations.” Male, 57 years old.

#### High out-of-pocket costs

For most people with lower income status, they want to earn enough for their daily necessities. They usually stay at work or look for jobs with better payments. Unlike their richer counterparts, even if they are aware of HIV prevention methods, the poor cannot buy condoms for safe sexual practice if they engage in it. Respondents highlighted that they had experienced similar responses from community members during the HIV/AIDS prevention activities.

“… Because the distribution of condoms has decreased and people are buying them on their own, people in poverty cannot afford to buy condoms”. Male, 50 years old.

“What kind of daily laborer buys condoms for 50 birr and uses them? They cannot.” Female, 31 years old.

### Theme five: resources-related challenge

Leadership roles lacked a fair distribution of resources. Despite the inadequate resources (e.g., shortage of condom supply), participants explained that the available resources were not distributed based on the need or number of populations at risk. Non-proportional allocation of resources challenged equitable service provision, and participants stressed the challenges of supply interruption and unequitable input distribution.

“There is a shortage of resources, no condom supply at all. There is a condom storage box in this building, but it has not been occupied with condoms for the last two years.” Male, 50 years.

“There is no fair distribution of resources. When resources are divided, it is the ability of leadership to properly distributed resources.” Female, 48 years old.

### Theme six: lack of tools and guidelines

All interviewees emphasised the challenges of not accessing recent tools and guidelines. Interviewees identified that reporting tools and training guides were prepared a long time ago and believe it should be changed to aiming for equitable service provision. For example, if the trainer brought a unique approach than the previous, they would listen to the trainer and take assignments to practice equity. In contrast, using a similar training approach and reporting tools that were prepared a long time ago, it could not improve equitable services provision.

“The manuals we’re using now are from a decade ago. They are not suitable for today’s generation, compared to the current technology-dependent period.” Male, 40 years old.

“The way we teach has not changed. People tell you to come up with a new method of training. For example, let’s say you moderated this module for two days (interviewee pointed into a module on the table). If you repeat the same person for the third time, what will he say? …. he/she will not happy hearing again.” Male, 57 years old.

Achieving equity in HIV/AIDS services needs to influence people’s resistance to HIV/AIDS information. They explained that there should be a welcoming training approach using recent information, showing the real suffering of living with HIV, such as costs of drugs, side effects of medications, the fate of the country due to the high HIV/AIDS burden, consequences that may happen to the new generations, and suffering from comorbidities. The interviewees agreed that people give due emphasis to cancer, blood pressure, and diabetes because the training they are giving about HIV/AIDS has not changed since a long time ago. Therefore, they believed that providing additional training about how life expectancy could be shortened if HIV infected with the presence of comorbid illness, including clinical and health care costs,

“The procedures or approaches in the sector are continuing as they were in the past. We must change our approach based on research. We have used discussion as a way of creating awareness for the past few years; what if a person does not want to discuss now? He hears it and hears it again and again, so he is bored with it.” Female, 26 years old.

The lack of a guideline or procedure to identify people who are far from services is another challenge to achieving equity. The absence or lack of strategy on how to reach parts of the population who are deprived of HIV/AIDS services results in difficulty achieving equity. The general perception was that the absence of guidance from the federal and/or regional offices negatively influenced service provision to the disadvantaged population.

“There is no specific method of procedure of identifying who is deprived of service delivery. We don’t have a system that focuses on people who we think have less knowledge and attitude, so we don’t have a unique strategy to work on these populations. There is no system that considers those who come from rural areas, have low economic status, are uneducated, or have a low level of education.” Female, 31 years old.

### Theme seven: inadequate understanding of equity in service provision

There was broad consensus on the presence of inequity in HIV/AIDS service provision. Among the consistently cited reasons for not achieving equity was an inadequate understanding of equity. Although respondents are participating in providing HIV/AIDS services to key groups of the population, they agreed that the limited understanding of social determinants of health process is causing or widening inequity in services. Some participants misunderstood the presence of inequity due to education status, which leads them to not deliver tailored services based on need. This is illustrated by the quote below from one of the respondents.

“… and in our office, we don’t deliver services based on education status because we believe all are well educated and got services equally.” Female, 40 years old.

Inadequate understanding of equity was described in other ways. They did not pursue the special strategy to the key and targeted disadvantaged groups because they misunderstood that those in need of services would come to the service delivery settings or would access services during general services provision to the public.

“Most of the work we do is general to the public. … I believe that we can find the inaccessible ones together. … Therefore, we do not follow a specific procedure because we think that those who are more susceptible to the disease or are not accessible to services will come to the service spot.” Female, 42 years old.

Sectors that are mainstreaming HIV and AIDS are also working for disabled individuals. Inclusive service provision was not in practice based on social determinants of health. Most believe that inclusiveness is at the system level, but it is not practiced due to inadequate understanding.

“The work we do is almost discriminatory or not inclusive. For example, when I was participating in delivering training previously, a blind man asked me to record my voice and give it to him. I gave him a notebook, a pencil, and a manual just so that he wouldn’t miss out, but if we ask if he really uses them, he doesn’t. No way. I just realised that the work I was doing was biased.” Male, 40 years old.

Inadequate understanding was due to a lack of information and training. They explained that it has been a long time since well-organised training towards equitable HIV/AIDS service provision was delivered.

“One of the problems is the lack of information about equitable service provision procedures. There is no consistent training on equitable service delivery.” Female, 42 years.

“Sectors can work towards equity if they have received short- or long-term training, but in the last five years, we have not given any training to a professional or focal person.” Male, 40 years old.

### Theme eight: cultural values, norms, and perception

The interviewees explained that cultural values and norms interrupted service provision. People are growing within the boundaries of culture and have developed habits that they do not want to explain fearlessly and confidently. Respondents believe that bad perceptions towards HIV/AIDS services (e.g., condom use) should be avoided. When the service providers met people that they believed lacked services, their attitudes towards services prevented proactive implementation. Respondents recommended about positive change in culture, norms, and perceptions.

“Society must bring about a cultural revolution to openly discuss sexual situations with youths. Cultural issues normally do not allow this, but we must break this.” Female, 42 years old.

“There is a problem with society’s attitude and perception. When we gathered the employees to create awareness, fewer than 20 or 30 people out of 105 employees attended. They tell you funny things… they said this virus is still not gone? … are you going to stress us despite HIV having disappeared.” Male, 37 years old.

“Our sector provides HIV testing to the partner if the partner’s test result is positive. I faced a problem with Males in the community. When women test positive for HIV, men (sexual partner) usually refuse HIV testing. I read them that it was due to males’ masculinity norm characters that barred them from using HIV/AIDS services.” Female 34 years old.

### Causal pathway for HIV/AIDS service inequity

We developed a conceptual model that illustrates the connection between the identified themes and the disparity in HIV/AIDS services. This model also addresses relationships among each essential structural feature. The diagrammatic synthesis will enhance common understandings among sectors that are mainstreaming HIV and AIDS. It may also inform and guide future empirical studies.

**Fig. 2:**
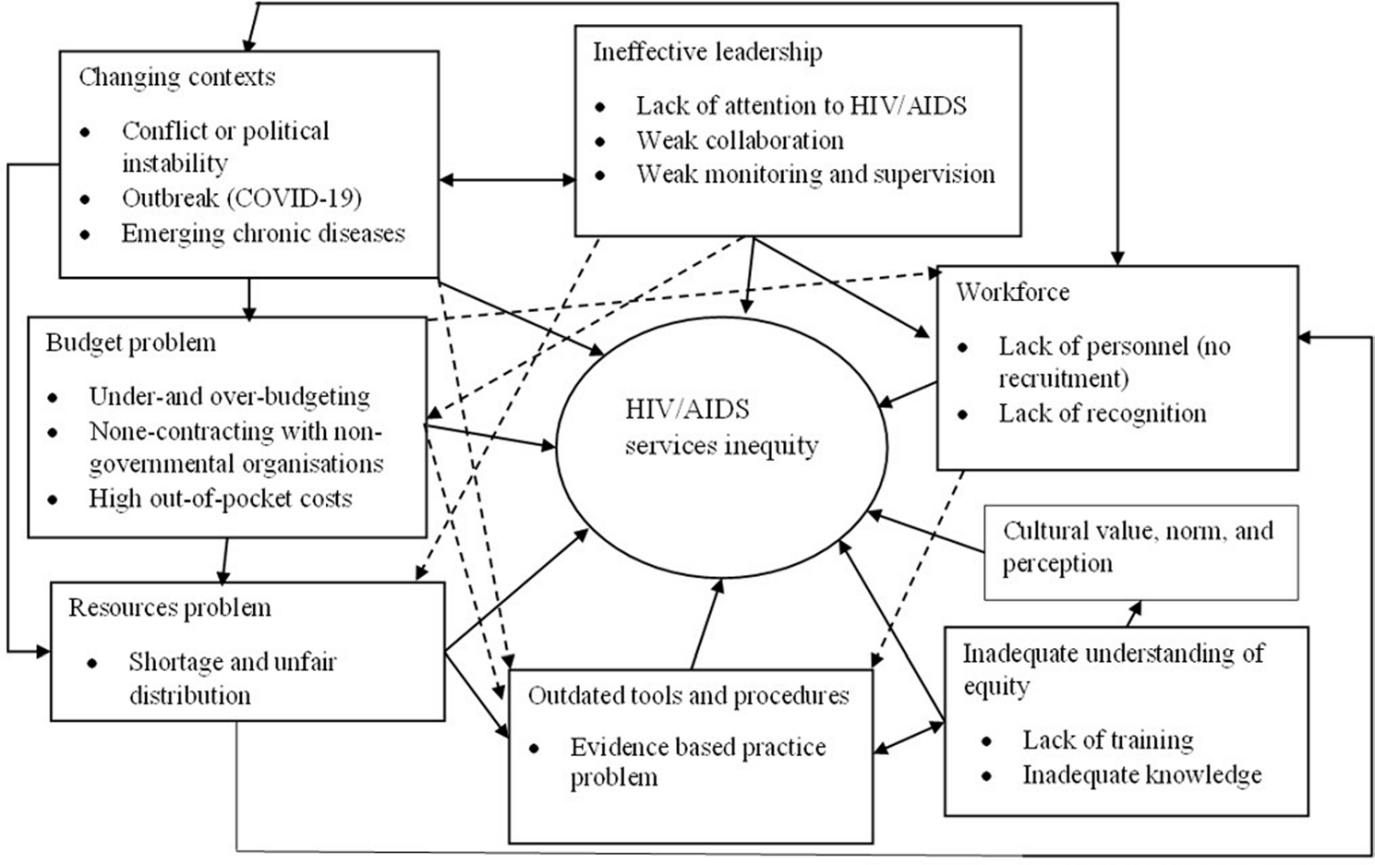
Causal pathway developed from the inductive analysis

The figure shows how key themes interact and contribute to HIV/AIDS service inequity, based on insights from discussions with participants. The resulting framework highlights contextual changes and emerging demands, which require material and human resources, pre-planned or unplanned budgets, and updating outdated tools and guidelines. This can make it difficult for service providers to reach those in need, and disadvantageous groups are unable to access HIV/AIDS services. Ineffective leadership, budgeting systems, shortages or unfair distribution of material resources, an inadequate workforce, tool and technical procedure unavailability, inadequate understanding of equity, and cultural norms all contribute to the inequity in HIV/AIDS services. The diagram does not distinguish between solid and dotted arrows, so we used dotted arrows to ensure clarity when one arrow crosses over another (Fig. 2).

## Discussion

Sectors that are mainstreaming HIV and AIDS have provided a rich context for the identification of challenges to achieve equity or causes of inequity in HIV/AIDS service, and that could lead to establishing future strategies.

Global health practitioners involved in health system are working in a holistic system to get a cross-cutting understanding of HIV/AIDS. While several sectors have substantial variation in their own roles and the system to combat HIV/AIDS, ranging from taking main roles to protect and promote the human rights of people living with HIV (UNAIDS) [49], financial support to HIV services (USA president’s emergency plan for AIDS relief/PEPFAR) [50], and assist service provision (WHO) [51], engagement in the closing gap in service utilisation is a common component, and hence the themes found in the current study are relevant in an international context.

Mainstreaming HIV/AIDS benefits from the integration of the health system and social welfare programmes to achieve equity in services [52]. Contextual changes, financial problems, workforce-related barriers, ineffective leadership, inadequate resources, lack of evidence-based tools and procedures, an inadequate understanding of equity, and cultural norms and values challenged the adoption and implementation of equity-based programmes and strategies. Federal and regional health bureaus have an opportunity to assist in countering these challenges that need agreement on solutions at the macro-level, which later being tailored at the meso- and micro-levels. Meanwhile, all mainstreaming sectors at the ministry and regional levels get the advantage of sharing responsibilities with the health bureau and working together to proactively react to the dynamic context and escalating economic costs.

Perspectives on sectors that are mainstreaming HIV and AIDS suggested that mainstreaming systems can create more integrated and collaborative energy for efficient resource use in achieving equity [53, 54]. The impact of mainstreaming to equity-based services could be optimised internally within sectors and built into a synergistic approach with external sectors towards the group of the populations who are far from or not using services. The synergistic partnership could create added value by leveraging the resources and concentrating collaborative activities on certain groups with common interests [55, 56]. The sectors that are mainstreaming HIV and AIDS are mainly self-funded, and therefore, individual sector commitment determines the greater contribution of the whole than individual sectors alone.

The themes identified in this study (inadequate understanding of equity, leadership, contextual changes, funding, workforce, resources, tools and guidelines, and cultural contexts) are closely aligned with the five out of six building blocks of health system function described in 2015 by WHO [57]. The health system building block framework describes leadership/governance, financing, access to essential medicines, health information systems, and a health workforce. Additionally, the three themes (economic domain/finance, lack of knowledge on equity, leadership/governance) and health system functions (five themes) are aligned with the social determinants of health framework described in 2010 by the WHO [58]. These two frameworks lay the foundation for system thinking and a roadmap for intervention implementation towards health and/or service equity. Other social determinants of the health equity framework comprises most of the current themes [31]. Another health equity measurement also encompasses socio-cultural context as one of the factors [59].

Ineffective leadership (absence of monitoring and supervision, negligence or inattention to HIV/AIDS, and weak collaboration) challenged equitable HIV/AIDS service provision. The role of sectors that are mainstreaming HIV and AIDS in demonstrating effective leadership towards equity will be critical. This resonates with the findings of Betancourt et al. who linked the role of leadership in health care equity through setting goals and priorities, ensuring that resources are allocated to the individuals who need them most, promoting diversity and inclusiveness, addressing poverty, and warranting the implementation of evidence-based practice [60]. Sadan and Braveman also discussed the role of monitoring and supervision of social determinants of health and health care equity; in the absence of this leadership role, one cannot realise whether sectors are aligned with standards and cannot identify service gaps [61, 62]. Supportive supervision provide feedback on staff performance in Haiti [63] and strengthen HIV/AIDS programmes in developing countries [64, 65]. More importantly, the role of leaders in creating collaborative activities is highly appreciated. Internationally, the UNs Commission Position on ending HIV through intersectoral collaboration was signed in May 2018 aiming to implement intersectoral collaboration in each country [66], but weak intersectoral collaboration cited as a barrier for equitable HIV/AIDS service provision or a reason for the existing inequities in the current study. This implies that sectors that are mainstreaming HIV and AIDS require leaders who can effectively engage and provide attention to the visible HIV/AIDS service disparity. It is more than a decade that recommended the need of leadership development among all sectors to combat HIV/AIDS [67].

There was a problem in giving attention to equitable HIV/AIDS service delivery by the public and leaders due to dynamic contextual changes and emerging needs. The happenstance of these contexts affected basic human values [68], while evidence underscored the need to persist on providing HIV service to encounter emerging or widened disparity due to social unrest [69]. Previous studies claimed similar findings: war and outbreaks disrupt services and cause inequity in service utilisation [70]. Similarly, the emerging and increasing epidemic of non-communicable diseases increased the burden on the health care system in developing countries, including Ethiopia, besides burden from communicable diseases [71]. Emerging non-communicable diseases (hypertension, diabetes, and cancer), outbreaks (e.g., COVID-19), and war or conflict divert leaders’ attention, financial allocation, resources mobilisation, and workforces if countries do not have prepared emergency plans [72].

Sectors that are mainstreaming HIV and AIDS described HIV/AIDS-related inappropriateness and inadequacy of budgeting as causes of service inequity. Under the current budgeting and payment model of mainstreaming sectors, a lack of adequate budget for reaching individuals far from services or those who have lower knowledge, attitude, and HIV testing is a significant barrier. Budgeting lacks transparency, making it difficult to understand how resources and services are designed for individuals who need them. This can limit mainstreaming sectors’ capacity to distribute resources fairly. The Ethiopian national strategy considers daily labourer as a target population in HIV prevention activities [14], but the current research revealed that high out-of-pocket costs (e.g., buying condom) create financial barriers for low-income individuals. Financial constraints were aggravated by the phasing out of NGOs’ contracts. The government contracted with NGOs to expand the limited public sector capacity by reaching certain geographic areas or populations [73]. In contrast, non-contracting with NGOs escalated the service coverage disparity between rich and poor because of financial deficiency and the inability to maintain services initiated by NGOs which was mentioned by the key informants in the current study. Reasonable budgeting, initiating, or strengthening local funds, economic empowerment, or support for the poor will be important solutions to handle financial problems. Prioritising public health budget and health financing also strengthen the workforce, availability of resources, and improved access to health services [74, 75]. Furthermore, contracting with local or international NGOs will support the country’s progress in achieving UHC [76, 77] that needs well-trained workforce to integrate NGO’s mission into the existing system.

Health workforce problem (absence of personnel and lack of recognition) caused inequity in HIV/AIDS services though continuous supports to health workforce internationally. The USA president’s emergency plan for AIDS relief invests about 1.2 billion united states dollar to support health workforce to sustain the HIV/AIDS response in African countries [78]. However, some sectors that are mainstreaming HIV and AIDS have no personnel to run mainstreaming HIV services, while some sectors have received neither financial incentives nor career development for their role in providing HIV/AIDS-related services. This lowers motivation, increases turnover, weakens collaboration, disrupts the work environment, and interrupts service delivery to those in need. Previous studies acknowledged the impact of health workforce on HIV/AIDS programmes [79, 80].

The unavailability of equity-related tools and resources was mentioned as a cause of HIV/AIDS service inequity. This finding aligns with a study in sub-Saharan African countries, where a lack of friendly tools was a common barrier to service accessibility to disabled individuals [81].

Another challenge lies in the inadequate understanding of equity in service provision. The limited grasp of social determinants of health process contribute to the widening inequity in services. This occurs due to a common misconception that individuals in need of services will naturally seek out service delivery settings or access services during general provision to the public, resulting from a lack of information and training. A scoping review on ‘connecting knowledge with action for health equity’ revealed that advancing health equity requires greater awareness and dialogue that entertain ways of knowledge translation [82]. Creating a dialogue to discuss how HIV/AIDS mainstreaming sectors can reach social classes that are distant from the services is crucial. While it is true that these sectors have recently targeted key population groups based on their HIV-risk levels, there remains a challenge. Some social classes have better access to HIV/AIDS prevention services, yet they are at a higher risk for HIV infection. For instance, urban residents have better knowledge about HIV/AIDS, exhibit accepting attitudes towards people living with HIV, and undergo HIV testing than rural residents over time [26–28]. However, paradoxically, HIV infection rates are higher in urban areas. Similarly, individuals primary, secondary and higher education levels were at a higher risk of HIV infection [42], despite having better knowledge, attitudes, and testing related to HIV compared to those with no formal education [26–28]. This may contribute to the misunderstanding of equity in health care services. This situation calls for collective dialogue and discussion on how the services should be provided to both urban and rural residents and for what specific purposes.

Furthermore, cultural norms (e.g., males’ masculinity gender norm) have an impact on service distribution and utilisation. This is supported by previous findings that men do not undergo regular health check-ups (e.g., HIV testing) unless they experience life-threatening or serious signs and symptoms [83–85]. This men’s masculinity behaviour exposed them to serious health risks [86]. Therefore, behavioural interventions may be important to improve males’ behaviour towards health care services because masculinity behaviour can be changed with context and time [87]. Positive behaviour in men can further influence women’s health [88].

Regarding the transferability of this study, the findings are applicable to other countries with similar socio-economic profiles to Ethiopia and facing similar challenges with mainstreaming HIV/AIDS. UNAIDS encourages HIV/AIDS mainstreaming sectors [89], and many of the challenges identified in this study are also recognised by international organisations such as PEPFAR and WHO [50, 51] and other frameworks [31, 57–59, 90]. However, there is room for further optimization to reach a broader community base, considering the extent of inequity. This study’s findings complement with a joint review commissioned by UNAIDS and United Nations Development Programme (UNDP), which involved key informants from three countries: Cambodia, Ghana, and South Africa [90]. Notably, one challenge identified during the process of mainstreaming HIV and AIDS in 2005 was a lack of shared understanding regarding mainstreaming AIDS [90]. Interestingly, shared understanding on mainstreaming may not be an issue in Ethiopia at this time. Instead, the current challenge lies in the fact that HIV/AIDS mainstreaming sectors lack clear understanding of equitable service provision. Additionally, limited participation of key actors was a challenge for mainstreaming process as identified by UNAIDS and UNDP in 2005 [90]. In contrast, there are many sectors involvement (despite limitations in engaging religious institutions) recently in Ethiopia, whereas the challenge is weak collaboration. Low commitment to mainstreaming AIDS, limited prioritization of HIV/AIDS, lack of alignment in budgeting processes, limited budgetary incentives, and tracking resources for AIDS that identified by UNAIDS and UNDP back in 2005 [90] continue to impact the equitable provision of services in HIV/AIDS mainstreaming sectors in Ethiopia. Hence, structural factors and cultural context play an important role in directing efforts and resources through mainstreaming sectors. The progress towards ending HIV/AIDS epidemics in Ethiopia will be accelerated through sectors that are mainstreaming HIV and AIDS if the identified structural factors are managed properly. The principle that the national mainstreaming sectors identified should be implemented in the way of addressing the causes of inequity in HIV/AIDS services. These principles are monitoring and evaluation, partnership, sustainability, a sense of ownership, gender equity and equality, multisectoralism, result-orientedness, a sense of urgency, avoiding stigma and discrimination, and confidentiality [7]. In general, the study’s findings can inform frameworks for HIV/AIDS service delivery and contribute to research in other countries.

## Strengths and limitations

The strengths of this paper lie in including study participants from multiple sectors, which provides a more diverse and comprehensive perspective on the issue being studied. Diverse representation enhances the validity and applicability of research findings.

The limitation of this study is that it does not include service users; it was not possible to consider the service users perspectives.

## Conclusions

This study identified key challenges that contribute to or exacerbate inequity in HIV/AIDS services. Implementation of tailored and evidence-based interventions has been central to minimising gaps in HIV/AIDS services. Therefore, sectors that are mainstreaming HIV and AIDS need to invest for emergency preparedness and response, identify effective leaders to maintain inter-mainstreaming sector convergence, make HIV/AIDS as public agenda, monitoring and evaluation, institutionalise responsive budgeting, develop alternative funds to maintain NGOs initiatives, provide ongoing and up-to-date training, create a common evidence-sharing platform, facilitate proper recruitment, education, and professional development of HIV/AIDS focal personnel, and adapting and implementing cultural safety interventions. Sectors that are mainstreaming HIV and AIDS might be effective and efficient if they integrate behavioural services for emerging non-communicable diseases that help them get attention from the public and leaders. It is essential to enhance HIV/AIDS mainstreaming sectors to provide tailored interventions for social classes, considering the varying levels of inequity in HIV/AIDS services. For instance, they can focus knowledge creation, stigma reduction, and HIV testing interventions on individuals with low-socioeconomic status, lower education levels, and rural residents. It is also essential to encourage men to undergo HIV testing, even though they already possess better knowledge about HIV/AIDS and maintain accepting attitudes towards people living with HIV. Further research is needed to explore the extent to which these challenges and solutions are embedded in policy documents to confront socioeconomic disparities. Additionally, a separate study may be necessary to explore users’ perspectives to get representative views.

### Electronic supplementary material

Below is the link to the electronic supplementary material.

Supplementary Material 1

Supplementary Material 2

## Data Availability

All data generated or analysed during this study are included in this published article [and its supplementary information files].

## Abbreviations

AIDS: Acquired Immune Deficiency Syndrome
COVID-19: Corona Virus 2019
HIV: Human Immunodeficiency Virus
NGOs: Non-governmental Organizations
PEPFAR: USA president’s emergency plan for AIDS Relief
UNAIDS: Joint United Nations program for HIV/AIDS
UNDP: United Nations Development Programme
WHO: World Health Organization
