# Supplementary material for: Equity in HIV/AIDS services requires optimization of mainstreaming sectors in Ethiopia
Source: BMC Public Health. 2024 Jun 1;24:1477. doi: 10.1186/s12889-024-19016-5 (PMC11144345; doi:10.1186/s12889-024-19016-5)
Supplement: Supplementary file 1 — Supplementary Material 1 [file 12889_2024_19016_MOESM1_ESM.docx]

## Supplementary file 1: Interview guides and reflection sheet

## Interview guides for stakeholder interview

- 1. Sector/organisation_____________
  2. Age______________
  3. Sex______________
  4. Educational status____________

We are fascinated to know your perspective on equity in HIV/AIDS-related service delivery. I would like to record this interview as long as you permit it. This helps me pay attention to your conversation, and it also allows me to capture the whole conversation accurately. All information will be confidential. Recorded audio will be kept in a secured place until we delete it. If at any point you would like me to turn off the recorder, please let me know. You have the right to refuse to participate in the interview, to stop the interview at any time for any reason, or to skip a question.

- 1. Please describe your background and involvement within HIV/AIDS-related service delivery.
- How long has the the role been?
- How long have you been in an HIV/AIDS-related role overall?
- Main responsibilities, including position in the sector and HIV/AIDS-related services
  1. What does equity in HIV/AIDS-related services mean to you?
     - Do you think inequity in HIV/AIDS exists? Why/why not?
  2. What challenges is your institution facing in achieving equity in HIV/AIDS-related services? or How do challenges cause inequity in HIV/AIDS services?

Probing when necessary:

- Probe multiple equity lenses for HIV services and challenges.
  - Horizontal equity (equal service for equal need)
  - Vertical equity (unequal needs receive unequal services)
  - Intersectionality inequity (how to see multiple disadvantaged individuals or groups)
- Probe leadership/governance
  - How the expanding number of functional institutions,
  - Inclusiveness in policy,
  - How equity is institutionalised,
  - Health system reform on equity,
  - How reach marginalised populations and displaced people,
  - How monitoring and supervision
  - Political willingness
- Probe how sectors plan/set strategies for delivering HIV/AIDS-related services
  - Presence of inclusive interventions for diverse populations
  - Tailoring services to ensure consumer acceptability
  - Service priority based on need
  - Use of key inequity-responsive care,
  - Culturally safe care offering services
  - How monitoring and supervision
- Probe how financing issues affect delivering HIV/AIDS services to all based on need.
  - Budgets issues
  - Investment in equity promotion
  - Flexibility of the financial system (responsive budget for unpredicted disasters)
  - Effective expenditure of resources
  - How monitoring and supervision
- Probe the source and dissemination of information.
  - Equity monitoring evaluation tool
  - Advocacy of health equity and social determinants of health
  - How monitoring and supervision
- Probe human resource issues related to HIV/AIDS services.
  - Distribution of individuals who are working towards HIV/AIDS related services,
  - Qualified/trained staff
  - How monitoring and supervision
- Probe medicines, supplies, and technologies issues related to HIV/AIDS services
  - Access services through phone call.
  - Allocation of drugs
  - Allocation of diagnostic tools (e.g. HIV test kit)
  - Allocation of supplies (e.g. condom)
  - How monitoring and supervision
    - Probe culture and societal value.
  1. What recommendations do you have to improve equity in HIV/AIDS services?
     - What would help you do a better activity related to the equity of HIV/AIDS-related services?
     - Overall recommendations for improving equity in HIV/AIDS-related services
  2. Is there anything else you would like to tell me about your thoughts on equity in HIV/AIDS-related services?

Thank you for all your participation and time!

## Interview Reflection Sheet

Participant Code:_____________

Participant status/role in sector:_____________________

Interview Date:_________________

Interview Place:________________________

Interview language:_______________________

Discussion focuses:

A short description of the core findings from the interview. These points highlight the real meaning of the interviewee and its distinctive nature or issues.

Any unique note/finding from the interviewee?

Do you think the interviewee was friendly and helpful? If he/she was not being forthcoming, recruit one additional interviewee from this similar sector.
