# Supplementary material for: Equity in HIV/AIDS services requires optimization of mainstreaming sectors in Ethiopia
Source: BMC Public Health. 2024 Jun 1;24:1477. doi: 10.1186/s12889-024-19016-5 (PMC11144345; doi:10.1186/s12889-024-19016-5)
Supplement: Supplementary file 2 — Supplementary Material 2 [file 12889_2024_19016_MOESM2_ESM.docx]

**Supplementary file 2: Amharic transcription contents followed by English translation for each participant**

**#1**

1. ስለ ኤች አይ ቪ ጉዳይ ያለብህን ኃላፊነት ብትነግረኝ

የኤች አይ ቪ ጉዳይና ስርዓተ-ጾታ ጉዳይ ባለሙያ ነኝ፡፡ ስለሆነም ከአምስት አመት በላይ በዚህ መደብ ላይ እገኛለሁ፡፡

1. ስለምትሰሩት ስራ በዝርዝር ብትነግረኝ

ኤች አይ ቪ በአንድ ጉዳይ የሚፈታ ባለመሆኑ በእኛ መስሪያ ቤት የዚህ ቫይረስ ጉዳይ በኢንዱስትሪዎች ላይ የግንዛቤ ፈጠራ፣ የመከላከል እና ክበባትን በመመስረት ልዩ ልዩ ስራዎችን መስራት፣ ወላጆቻችን በቫይረሱ ያጡ ልጆችን በገንዘብ ይደግፋል፡፡ ሰራተኞች ደግሞ ለተገልጋዩ፣ ለቤተሰብና ለጎረቤታቸው ስለበሽታው ስርጭት መከላከልና መዛመት መረጃ እንዲሰጡ ያደርጋሉ፡፡ በቢሮ ደረጃ በየወሩ እቅድ ነበረን፡፡ አሁን አሁን ግን ከሰራተኛው መዘናጋት የተነሳ በአቀድነው መጠን እየሰራን አይደለም፡፡

እነዚህን ስዎች ለመስራት የምትከተሉት ስልት ካለ ብትነግረኝ፡፡ ስትራቴጂ እቅድ መሰረት አድርገን እንሰራለ፡፡

አልተሰራም የምትለው ነገር ይኖራል ብለህ ካሰብህ ብትነግረኝ፡፡ የመዋቅር ችግር አለ፡፡ የሰው ሓይል እጥረትና መዋቅር ችግር አለ፡፡

1. ተደራሽ ያልሆኑ የማህበረሰብ ክፍል ካሉ ብትነግረኝ

በስራችን ብዙ አጋር ድርጅቶችና ሰራተኞች አሉን፡፡ በበጀት እጥረት ምክንያት ተደራሽ እያደረግን አይደለም፡፡

ትኩረት የሚደረግባቸው የማህበረሰብ ክፍል ካችሁ ብትነግረኝ፡፡ ትኩረት የምናደርገው ሰራተኞቻችን ላይ ነው፡፡

ተጋላጭ የማህረሰብ ክፍሎች ብለን የምንሰራባቸው የህግ ታራሚዎች፣ እረጅም እርቀት አሽከርካሪዎች፣ ሴተኛ አዳሪዎች እና በሽታው በብዛት የሚገኝባቸውን ነው፡፡ ገጠር የማህረሰብ ክፍልን ሰራተኞች ወደ ቤተሰቦቻቸው ሲሄዱ እንዲደርሱ እናሳስባለን ነገር ግን ውጤቱን መለካት አልቻልንም፡፡

1. ተደራሽ ለማድረግ ተግዳሮት ካለ ብትጠቅስልኝ

ተቁዋማት የጋራ እቅድ እናቅዳለን፡፡ ነገር ግን ትኩረት እየተሰጠው አይደለም፡፡ በህብረተሰቡም እንደ ሴክተርም ዝንባሌው አሁን አሁን እየተከሰቱ ላሉ በሽታዎች ነው፤ እንደ ደም ግፊት፣ ስኩዋር ለመሳሰሉት፤ ማህበረሰቡ ኤች አይ ቪ ደግሞ መድሃት እየወሰዱ አስር እና ሀያ አመት መኖር ይቻል አይደል እንዴ ነው የሚልህ፣ ሰው አቅልሎ ነው እያየው ያለ፡፡ ምን የኤች አይ ቪ ቫይረስ መከላከል ነው እናውቀዋለን የሚሉ እሳቤዎች አሉ፡፡ አመራሩም ትኩረት እየሰጠው አይደለም፡፡ ይህን ደግሞ ከዚህ በፊት በነበረን ስብሰባ ገምግመናል፡፡ ስለ ኤች አይ ቪ ስናወራ እሳቱ ተዳፈነ እንጅ ጠፋ ማለት አይቻልም፡፡ 2 ፕርሰንት ማለት አንድ ጊዜ የግንዛቤ ማስጨበጫ እንደሰጠህበት ያበቃለታል፡፡ ነገሩን እንደ አጀንዳ ወስዶ በማውራትና በመወያየት በኩል ችግር አለ፡፡

ሌላው ጦርነት ስራችንን በአግባቡ እንዳንሰራ እያደረገ ነው፡፡ ተደራሽ ያልሆኑ በልዩ ሁኔታ መስራቱ ይቅርና የወል ስራም መስራት አይቻልም፡፡ በተለይ ጦርነቱ እየተካሄደ በአለበት አካባቢ እና አዋሳኝ ወረዳዎች ላይ አይታሰብም፡፡ በተመሳሳይ ሰራተኞቻችን አብዛኛውን ኢንዱስትሪ ላይ ያሉ ናቸው፡፡ እናም ፋብሪካዎች ሲወድሙ ሰራተኞች ይተናሉ፡፡ ስለዚህ ማግኘት አንችልም፡፡

የሰው ኃይል እጥረት አለ፡፡ በክልሉ ወይም በባህር-ዳር ከተማ ያለውን ሰራተኛ ሁሉ በ2 ሰራተኞች ተከታታነት ያለው ኤች አይ ቪ ጉዳይ ለመስራት ያስቸግራል፡፡ ምክንያቱም መኪና አቅርቦት የለም፡፡ የግብዓት እጥረትም የለም፡፡ በትንሹ ኮንዶም የለም፡፡

የማህበረሰቡ ለኤች አይ ቪ ጀሮ የማይሰጠው የተለመደ፣ ሳቢና ማራኪ ያልሆነ መንገድ ነው የምንከተለው፡፡ ወቅቱን የሚመጥን የስልጠና ማንዋል እንኩዋን የለንም፡፡ ሌላው ግን ሰዎች መድሃኒት እየወሰዱ ስለሚቆዩ በሽታቸውን አቅልሎ ማየት አለ፡፡

እኛ ግን እንዳጠቃላይ የኤች አይ ቪ ጉዳይ ዘርፈ ብዙ ስለሆነ ተቁዋማት ተቀናጅተው አይሰሩም፤ እጅግ በጣም ትልቅ ክፍተት ነው ያለ፡፡

አዳዲስ የሚፈጠረው ትውልድ ግን ለኤች አይ ቪ ለው መረጃ ብዙም አይመሰልኝም፤ አንድ ወቅት ላይ ግን የተዳፈነ ነገር እንዳይፈነዳ ስጋት አለኝ፡፡

1. ቢስተካከሉ የምትላቸው ነገሮች

ዩኒቨርሲቲዎች ላይ ትኩረት አድርጎ ቢሰራ ሱስ በብዛት ይስተዋላል፡፡ አዲስ አሰራር፣ ሳቢና ማራኪ በሆነ መንገድ፡፡ ተቁዋማት ተቀናጅተው ቢሰሩ፣ ትም/ት ቤቶች ላይ መሰራት አለበት፡፡

**#1**

1. Please tell me about your responsibility regarding HIV/AIDS

I am a focal person of HIV/AIDS and gender issues. Therefore, I have been in this position for more than five years.

2. Please tell me in detail about the work you do

Since HIV is not solved by one issue, in our office, we are doing various activities on industries to create awareness, prevention activities and establish clubs on the issue of this virus, and financially support children who have lost their parents to the virus. Employees also provide information to the client, family and neighbors about the prevention and spread of the disease. At the office level we had a monthly plan. But now we are not working as much as we planned due to the negligence of the staff.

3. Let me know if there is a strategy you follow to perform these activities.

We work based on a strategic plan.

4. If you think there are something that is not done, please tell me.

There is a structural problem. There is a lack of manpower and structural problems.

1. Please tell me if there are any segments of the community that you could not reach.

We have many partner organizations and employees who need HIV/AIDS services in our work. However, we could not reach all due to lack of budget.

1. Please let me know if you have a community members that you would like to give special attention to.

We focus on our employees. The vulnerable sections of the population we work with are prisoners, long-distance drivers, prostitutes, and those where the disease is prevalent. We urge our employees to teach their rural families when they go to rural families because most of our employees are originally from rural communities, but we have not been able to evaluate these activities.

1. Please tell me any challenges to deliver equitable services.

Sectors have a shared or a joint plan. But attention is not being given. The community members and sector’s attention is usually diverted towards the diseases that are currently emerging, like diabetes, hypertension and similar conditions. When you want to take to people, they responded that it is possible to live for ten or twenty years while taking medication for HIV/AIDS… what are you taking we already aware of HIV prevention methods. People take HIV as a simple as lightly. The management is also not paying attention. We already agreed and discussed this in our previous meeting.

However, we have to know that when we talk about HIV seems declined, it is not possible to say that the fire has died down. 2 percent budget means once you give a one-time awareness creation activity, it will be end-up. There is a problem with taking HIV/AIDS as an agenda and discussing it.

Another, war/conflict is preventing us from doing our jobs properly. It is not possible to do usual work, let alone work in special conditions that are inaccessible. It is not considered especially in the area where the war is actively going on and the bordering districts. Similarly, most of our employees are work in the industries. And when factories are destroyed, workers leave the place or industries. So, we can't find them.

There is a shortage of manpower. It makes it difficult to reach all employee in the region or Bahir Dar city by only 2 personnel. consecutive HIV cases. Because there is no car supply. There is no shortage of resources. No condom at all.

**#2**

1. ስለ አለብሽ ኃላፊነትና ስለምትሰሩት ብትነግሪኝ

እውነት ነው ሴክተሮች ሜኒስትሪሚንግ አደርገን እንድንሰራ ይፈለጋል እና በዛ መሰረት የኤች አይ ቪ ፎካል ሁኘ እንድሰራ ከአለብኝ የስራ ኃላፊነት ተጨማሪ እንድንሰራ በተሰጠኝ ኃላፊነት መሰረት ያው ከጤና ቢሮ ጋር እየተነጋገርነ የምንሰራው ስራ አለን፡፡ ይህ ሲሰራ ደግሞ ጤና ቢሮ ያለው ዘርፈ-ብዙ የሚስተባብረው በየተቁዋቱ እንዲሰራ ይለጋል፡፡ የምንሰራው ስራ በተቁዋሙ ያሉሰራተኞች ከተቻለም ይህ ክልል ስለሆነ እስታችኛው መዋቅር ድረስ በኤች አይ ቪ ዙሪያ ውይይት እንዲያደርግ አንዱ ነው፡፡ ከዚህ በተጨማሪ ደግሞ ኤች አይ ቢ በደማቸው ያለባቸው ሰዎች ቢሮው መደገፍ በሚገባው ልክ መብታቸው እንዲከበርላቸው፣ የአቅም ችግር ካለም በኢኮኖሚ እንዲደገፉ ማድረግ ነው አንዱ አላማው፡፡ ከዚያ ውጭ ደግሞ በማህበረሰቡ ውስጥ በኤች አይ ቪ ምክንያት ወላጆቻቸውን ያጡ ህጻናትን ተቀብለን እናሳድጋለን፡፡ ሌላው በተቁዋማችን ስራዎች እንዲሰሩ ይደረጋል፡፡ ለምሳሌ ተጋላጭ የሆኑ ሴቶችን ማገዝ፤ በተለይ ሴተኛ አዳሪዎች ላይ እያቀደ እንዲሰራ፤ ከዚህ በፊት ወጣቶች ላይ ይሰራ ነበር፡፡ በተለይ ይህ ሁለቱን አመት ኮቪድም፣ ጦርነቱም ስራዎች የተቀዛቀዘበት ሁኔታዎች አለ፡፡ በአሁን ሰዓት ሶስት ወንድ ሶስት ሴት ልጆችን እናሳድጋለን፤ በየወሩ 500 ብር እከፍላለን፡፡ ሰራተኛው ከደሞዙ አንድ ፐርሰንት በየወሩ ይከፍላል፡፡ ሴክተሩም አልፎ አልፎ እዳለው በአቅሙ ያገዛል፡፡

ከዚያ ውጭ ደግሞ በስልጠና፣ በክበባት ሴቶችን ማሰልጠን፣ እራሳቸውን በኢኮኖሚ እንዲያበቁ ይሰራል፡፡ ከዛ ውጭ ስራ ላይ ውይይት ብዙም አይደለም፤ አምና ላይ ተገናኝተናል፤ ዘንድሮ ገና አስበናል ውይይትም አስበናል፡፡ ሰው ሰልችቷል መስማት አይፈልግም፡፡ እንደ እንትን ብትይዘው አካሄዱን ለወደፊት ቢስተካከል ብየ የማስበው የስራ ላይ ውይይቱ ሰው በቃ ኤች አይ ቪን… አሁን ምንድን ነው ካንሰር፣ ግፊት፣…ሌላው ስኩዋር በተኛበት እያስቀረው ነው ኤዲስ ያለባቸው ሰዎች 20 ዓመት እየኖሩ ነው ምንድን ነው ብሎ የመከላከል ተግባሩ እየተዳከመበት ያለበት ሰዓት ላይ ነው፡፡ እና ስብሰባ ስትጠራው ከዚህ የምትጠሩን ምንድን ነው ትምህርቱን አንደሆነ 20 30 ዓመት ብለነዋል ወጣ አድርጋችሁ ብታዝናኑን ምንም አዲስ ነገር የለም ይላሉ እና ምንድን ነው… ገንዘብ ደግሞ ሁለት ፐርሰንት ተብሎ በጀት አለ፡፡ ለምሳሌ እኔ እስከ አራት መቶ ሺህ ድረስ ብር ነበር፡፡ እና ሰራተኛው ወደ ሁለት መቶ አካባቢ ስለሆነ ወጣ ብሎ ማድረግም አልተቻለም፡፡ እና ከዚሁ እናድርግ ብለን ከዚሁ ስናደርግ ሰራተኛው አይገኝልህም፤ የተገኙትም አዳራሹን እየተውት ነው የሚወጡ፡፡ እና የሆነ ወረዳም ላይ ቢሆን አድርጎ ሻይ ቡና መድቦ ሰው ይመጣል፤ ከመጣ ደግሞ መረጃውን መስማቱ አይቀርም እና ቢደረግ ብለን እየተነጋገርን ነው ያለን፡፡ ቢያንስ እንኩዋን ባለሙያ አለም አቀፍ መረጃ ምን ይመስላል፣ እንደ ክልልስ ምን ላይ ነን፣ እንደ ሀገርስ ምን ላይ ነን፣ እንደ አካባቢስ ምን ላይ ነን የሚለውን መነጋገር አስፈላጊ ነው የሚል አተያይ አለኝ፡፡ ግን ሰው የመሰላቸት እና አቅልሎ የማየት ሁኔታ አለ፡፡ ይህን ይመስላል እንግዲህ ያለው ሁኔታ፡፡ በፊት የኮንዶም ስርጭት አገልግሎትም እንሰራ ነበር፤ ከዚህ ህንጻ ላይ የኮንዶም ማስቀመጫ ቦክስ ነበር፤ እና የዛሬ ሁለት ዓመት ጀምሮ እጥረት አለ፡፡ እና እኔ ፎካል ስለሆንሁ ማለቴ ደርቤ ሲያመቸኝ የምሰራው እንጂ ዋና ስራ ስራየ ስላልሆነ ወደ ሌላው ስራ ሳተኩር ይረሳል፡፡ የሆነ ወቅት ላይ ስጠይቃቸው የለም ካሉኝ እተወዋለሁ፡፡ በእኛ ቢሮም ግዢውን እንድንፈጽም የሚያስችል አሰራር የለም፤ ኮንዶም ከጤና ቢሮው ነበር የምናመጣ፡፡ በጀትም ቢሆን ሁለት ፐርሰንት ተብሎ በየመስሪያ ቤቱ የሚመደበው ሁሉንም ላንጠቀመው እንችላለን፡፡ ከሁሉም መስሪያ ቤት ያለውን ሰብስቦ በአንድ ላይ አድርጎ ጠንካራ ስራ ቢሰራ ጥሩ ነው፡፡ በጀቱ ካለ ደግሞ ከታች ወረዳ ድረስ ትወርድና ታወያይበታለህ፣ ግንዛቤ መፍጠር ይቻላል፡፡ እኔ ለራሴ ከዚህ ከስራ ቦታ ተወያዩ ከሚባል ገንዘቡ ከሚጠፋ ሌላ ስራ ቢሰራበት እላለሁ አንዳንድ ጊዜ፡፡ ሪፖርቱም ም ያክል በጅታችሁ ምን ያክል ተጠቀማችሁ አይት እንጂ ምን ታቅዶ ምን ተሰራ የሚል አይደለም፡፡ ባለፈው ወር ከፌደራል የመጣ ሰው መጥቶ ስለተጠቀምው በጀት ጠየቀኝ እና እኔም በጀት አይደለም ዋናው ነገር ምን ታቅዶ ምን ተሰራ የሚለው ላይ ነው ማተኮር ያለብን አልሁት፡፡ ያ ደግሞ ከሆነ ገንዘቡ በአንድ ቁዋት ሁኖ ጠንካራ ስራ መስራት ነበረበት፡፡ አሁን ያለው ምንድን ነው ፎካል ፐርሰን የሚባለው መዋቅር አለ፣ ሪፖርት እልካለሁ፣ እንደ ምንም ብየ ከአመት አንድ ጊዜ ወይም ሁለት ጊዜ ውይይት አደርጋለሁ፣ ያችን አያይዤ ሪፖርት አደርለሁ፡፡ ግን ሰው የእውነት ተቀይሩዋል የሚለው ጥርጣሬ አለኝ፡፡ እስከወረዳ ግን እየተገናኘሁ አይደለሁም፡፡ መዋቅሩ እስከ ዞንና ወረዳ አልወረደም በእኛ ቢሮ ስር፡፡

1. ይህ የምትሰሩት ስራ በሱቶችና ህጻናትም ሆነ በማንኛውም ማህበረሰብ ኤች አይ ቪን የተመለከተ የምትሰጡት አገልግሎት ለሁሉም ተደራሽ እንዳይሆን የሚያጋጥማችሁ ተግዳሮት ምንድን ናቸው

አንደኛው የበጀት ችግር አለ፡፡ ሁለተኛ ማህበረሰቡ ለኤች አይ ቪ ያለው አመለካከት አሁን ጨመረ ሲባል ትሰማለህ እንጂ እደ ተራ እና ቀላል በሽታ ነው እያየው ያለ፡፡ ለምሳሌ እድሜ ልክ መድኃኒት የሚወስዱ ሰዎች እኮ በመድኃኒት ጎንዮሽ ጉዳት ተጠብሰው መኖራቸውን ሰው አይረዳውም፡፡ ሰው ግን ከውጭ ሁኖ የሚያየው አቅልሎ ነው፡፡ ሶስተኛ በዘርፉ ላይ ያሉት አሰራሮች ድሮ እንደ ነበረው ነው እየቀጠለ ያለ፡፡ አይተህ ግን በጥናት ላይ ተመስርተህ መቀየር አለብህ የአሰራር ስልትህን፡፡ ለምሳሌ ድሮ ማወያየት ከሆነ አሁን ሰው መወያየት ካልፈለገስ…፣ ሰምቶት ሰምቶት አሁን እንወያይ ብትለው አይፈልግህም፡፡ እንዲያውም ያሾፋል፤ ስብሰባ ስትጠራው አረዲያ አሁን ኤች አይቪ (የሹፈት ስላቅ ያለበት) ነው…እንዲያው ዝም ብለሽ ሻይ ቡናውን በይንማ እባክሽ ነው የሚሉህ፡፡ ግን ደግሞ ተገናኝተህ ስታወራው ሰው መረዳት ይጀምራል፡፡ ስሜትን በሚኮረኩር መልኮ ልጆቻችን እየጠፉ ነው እና መሰል ስሜት ነኪ ጉዳዮችን እያነሳህ ስትነግረው ይሰማሃል፡፡ የሚለውጥ ነገር መሰራት አለበት ብየ አምናለሁ፡፡ ሰው ካንሰር፣ ኩላሊት ችግር ከተባለ እራሱን ይዞ ያዝናል፣ ይደነግጣል እና ለቫይረሱ ግን መላመድ ነገር አለ፡፡ ለውጥ በሚያመጣ መልኩ ሁሉም ተተኪውን ትውልድ እዲያተርፍ በሚያስችል መልኩ ሰልጠናዎችስፈልጋሉ፡፡ አሁን ግን ወቅታዊ ጉዳዩም ጭራሽ እንዲረሳ ሁኑዋል፡፡

1. ዝቅተና የገቢ መጠን ያላቸው፣ እሩቅ የሚኖሩ፣ዝቅተኛ የትም/ት ደረጃ ላይ ያሉትን ለይቶ በመስራት እና ለሁሉም እኩል በመድረስ በኩል ምንድን ነው የሚቸግራችሁ..ከላይ የጠቀስሽልኝ (በጀት፣ ወቅታዊ ጦርነቱ፣ ኮቪድ፣ ትኩረት አለማድረግ ያልሽኝ እንዳለ ሁኖ)

አንዳንድ ጊዜ እኮ በትንሽ በጀት ብዙ ነገር መስራት የሚቻልበት ሁኔታ ሁሉ መፍጠር ይቻላል፡፡ ነገር ግን ቀራጥ መሆን ያስፈልጋል፡፡ ነገር ግን ኮሚትድ መሆን ይጠይቃል፡፡ ሌላው አንዳንድ መስሪያ ቤት ላይ በባለሙያ ነው የሚሰራው፣ አንዳንድ ላይ ደግሞ ልክ እደ እኔ እንደተጨማሪ ስራ ተደርጎ ሰው ይመደባል፡፡ እና በትክክለኛነት ሃላፊነትና ተጠያቂነት በአለው ሁኔታ ሰው መመደብ አስፈላጊ ነው ብየ አምናለሁ፡፡ አንድ ሰው እዴት ነው ሁለትና ሶስት የስራ ዘርፍ የሚመራ ያውም በክልል ደረጃ፡፡ አንዳድ ጊዜ የሴቶች፣ ህጻናትና አካል ጉዳተኞችን ጉዳይ ስለምሰራ ጤና ቢሮ ስብሰባ ሲጠራ ሌላ የማይመለከተውን ሰው ሁሉ ወክየ እልካለሁ ስልጠናው ላይ እንዲሳተፍ፡፡ እና ከሪፖርት የዘለለ ስራ መሰራት አለበት፡፡ ተደራሽነቱ በምን አግባብ ይሰራ የሚል ስትራቴጂ መንደፍ ያስፈልጋል፡፡ ከሁሉም ሴክተሮች ጋርም ተቀናጅቶ የመስራት ችግር አለ፡፡ ትም/ት፣ ጤና እና ሌሎችም በጋራ በመሆን መስራት ያስፈልጋል፡፡ ሌላው ጉዳዩን በፖለቲካ አመራሩ ትኩረት አለማግኘቱም ጭምር ይመስለኛል፤ እደ በፊቱ መሬት የነካ ስራ፣ ቫይሱን አጀንዳ አድርጎ ቶሎ ቶሎ የመወያየት ሁኔታ አይስተዋልም፡፡ አሁን አሁንማ ከኤዲስ ይልቅ ለኮቪድ የበለጠ ጀሮ ተሰጥታል፡፡ እንደ መጣ አካባቢ እንደ ነበረው አይፈራም፤ አንድ ቀላል በሽታ የያዘውን ያክል ኤዲስ ቢይዘው ይኖራል የሚል አመለካከት አለ፡፡ ጤና ዘርፉ ጠንክሮ መምራት አለበት፡፡ ሰው ደግሞ ሳያውቀው እናውቃለን ብሎ የሚያስብ አለ፡፡ ስሙን መስማቱን እንጂ እዲያው ሁሉንም የመከላከል መንገዶች ብትለው በፍጥነት ጊዜ ሳይወስድ የሚዘረዝርልህ ሰው ጥቂት ሊሆን ይችላል፡፡ ትልቁ ነገር ትኩረት መስጠት አለበን፤ ትኩረት ካልተሰጠው አሁን አሁን በየመንገዱ ምሽት ላይ የሚቆሙ ሴቶች በዝተዋል፤ ስለዚህ በሽታው በስፋት የመተላለፍ ሁኔታው ሰፊ ነው፡፡ ባለሙያ ቢመደብ ግን ስራየ ብሎ እቅድ ያወጣል፣ በጀት ያስበጅታል፣ በትክክል ስራው ይሰራል፡፡ እኔ ግን ይህን ላደርግ አልችልም፡፡ ሰራተኛው ሻይ ቡና እያለ እንዲወያይ ነው የሚፈለግ በዓመት አንድ ጊዜ ወይም ሁለት ጊዜ እንደምንም አድርገን ሞክረን ሪፖርት እንፈልጋለን ጤና ቢሮውም የሚፈልግ ይህን ስለሆነ፡፡

1. ቀረ የምትይው ወደፊት የሚሰራ ወይም እንደ ተግዳሮት የምታነሽው ሐሳብ ካለ

ሁሉንም ከላይ የጠቀስሁት ነው ባለሙያ ይመደብ፣ የተለየ የስልጠና አሰጣጥም አካሄድም ሆነ ስልት መንደፍ፣ ወርዶ እያንዳንዱን ማህበረሰብ ማወያየት፣ በይበልጥ ደግሞ አገልግሎት የማይደርሳቸውን የማህረሰብ ክፍል ማወያየት ያስፈልጋል፡፡

**#2**

1. Tell me about your responsibilities and what you do.

It is true that we are required to manage the sectors and we are required to work as an HIV focal person. When this is done, the multi-sectoral action and policy of the health office communicate me to work together. The work we are doing is one of the things that the workers in our sector can discuss, if possible, because this is the region, we go down up to the lower structure /district/ to discuss on HIV.

In addition to this, one of the objectives is to make sure that the rights of people with HIV are respected as the office should support them, and that they can be supported economically if they have economic problem. Apart from that, we accept and raise children who have lost their parents due to HIV in the community. We are currently raising three sons and three daughters. We pay 500 Birr every month. The employee pays one percent of his salary every month. The sector also support based on its capacity. The other will be made to do the work of our team. For example, helping vulnerable women; To work especially on prostitutes. At previous time, we were work on youths.

Especially these two years of covid and the war has slowed things down. Apart from that, we work to train women through training and clubs, so that they can support themselves economically.
Other than that, there isn't much discussion at work; We met last year. This year we have thought about it, and we have thought about discussion. People does not want to hear that he is bored about it. They responded currently that HIV... What is it now? Cancer, blood pressure... diabetes is keeping him in bed, but people with AIDS are living for 20 years; so what you are talking about...?. And when you call a meeting, they responded as ‘what do you call us from here? We heard about HIV just 20 or 30 years old and they say there is nothing new... please come out and entertain us…’ Money is budgeted as two percent, but not enough. For example, our sector has around 400 thousand Ethiopian birr. And since the staff is about two hundred, it was not possible to do it outside. And when we say let's do it from here, the worker will not be available for you; Those come to discuss are leaving the hall before we end the meeting. So what I suggest is to take them and make in other town because those who come there should be stay until the end of the meeting; if they wait, they have a probability listen what is new on HIV and may take in the message.

I believe it is important to talk about what international information looks like, how we are doing as a region, what we are doing as a country, and what we are doing as an area. But there is a tendency for people to get bored and take HIV too simple. This seems to be the case. We used to do condom distribution service too. There was a condom storage box on top of this building. And there has been a shortage for two years. And because I'm focal, I mean what I do when I'm comfortable, it's not my main task, so when I focus on other tasks, I forget. When I ask them at some point, if they say no, I leave it. There is no procedure in our office that allows us to make the purchase. We used to get condoms from the health office. We may not use all the two percent budget allocated to each office. It would be good if we could gather what we have from all sectors together and do a strong job. If there is a budget, you can go down to the lower district and discuss it; awareness can be created for all people as a result. Sometimes I say to myself, I wish I could do something else instead of workplace discussion and meeting where the money is lost. The report is about how much you have used, not what was planned and what was done. Last month, someone from the federal government came and asked me about the budget that was used, and I told him that it is not the budget that matters, we should focus on what is planned and what is done. If that was the case, the money would be collect together and deposited in a single account to work better job. What is it now…? There is a structure called focal person, I send a report, whatever I say, I have a discussion once or twice a year, I attach it and report. But I doubt that people have really changed. But I am not connected to the district. The structure has not gone down to the zone and district under our office.

2. What are the challenges you face in making the services you provide related to HIV not accessible to all?

One is the budget problem. Second, you will hear that the society's attitude towards HIV has increased, but it is seen as an ordinary and simple disease. For example, community is not understood that people who take medicine all their life are suffering from side effects of medicine. But a person from the outside sees it lightly. Thirdly, the procedures or approaches in the sector are continuing as they were in the past. You see, but based on research, you have to change your approach. You may use discussion for the past years, what if a person does not want to discuss now? He hears it and hears it again and again, so that he is boring on it. Even, he mocks on you (እንዲያውም ያሾፋል). When you call a workplace meeting to discuss on HIV, they may laugh on you and asks you to give them a tea or coffee. But when you meet and talk to him, they start to understand if you raise sensitive issues in a very emotional way and you are raising such sensitive issues, like our children are disappearing. I believe that something needs to be done. If a person is told about cancer or kidney problem, he will hold his head and himself, he will be shocked, but there is something to adapt to the virus. Training is needed in a way that makes a difference so that everyone can benefit the next generation. But now this issue seems totally forgotten.

3. What is the problem for you by targeting those with low incomes, those who live far away, and those with low education levels and to reach them equally?

Sometimes it is possible to create a situation where a lot can be done with a small budget. But you have to be persistent and committed. But it needs commitment. The other one is done by a professional in some offices, and in others, a person is assigned as an extra job just like me. And I believe that it is important to assign a person in a position to do responsibility and accountability. How can one person lead two or three sectors of work, that is at the regional level? Sometimes, because I deal with the issues of women, children and disabled people, when a meeting is called by the health office, I send everyone else who is not concerned to participate in the training. And more work than reporting must be done. It is necessary to design a strategy for how the equity will be achieved.

There is also a problem of coordination with all sectors. Education, health and others need to work together. I think the other thing is that the political leadership did not pay attention to the issue. They don't notice the situation of discussing frequently and work that touched the ground. Now more attention is given to covid than to HIV/AIDS. Community is not afraid HIV as the time when the epidemic happens. There is a view that if he has AIDS, he will live as much as he has a simple disease. The health sector must be managed and lead strongly. There are people who think that they know without knowing it. But, if you ask them immediately tell you all the ways to prevent it, there may be few people who will quickly list it for you. The biggest thing is that we have to pay attention. If it is not paid attention to, now there are many women standing on the streets at night (sex workers). Therefore, the spread of the disease will be widespread. If an expert is assigned, he will make a plan, adjust the budget, and do the job properly. But I can't do that. It is required for the employee to discuss while having tea and coffee once or twice a year because the health Bureau need this.

4.If there is an idea that will work in the future or that you can take as a challenge, please tell me.

All I have mentioned above is to appoint an expert on the field, design a unique training method and strategy, go down and discuss each community, and more importantly, reach and discuss the underserved sections of the community.

**#3**

1. እባክዎ ስለእርስዎ ይንገሩኝ

የሁለተኛ ዲግሪ የትም/ት ደረጃ፡ የሴቶች፣ ኤዲስ አና ልዩ ፋላጎት ዳይሬክተር ለ1 ዓመት በዚህ ዙሪያ ሰርቻሉ፡፡ እድሜ 31 ዓመት

1. የምትሰሩትን ስራ በዝርዝር ይንሩኝ

ሰራተኞችና እና ተማሪዎች ላይ አዌርነስ ክሬሽን እንሰራለን፡፡ ክለብ ማቁዋቁዋም፣ የኤዲስ ቀን ማክበር፣ ፒር ፕሬዠር እንዳያጠቃቸው፣ ወላጆቻቸውን ላጡ በገንዘብ እንደግፋለን (ለ21 ልጆች) ከሰራተኞች ገነዘብ በመሰብሰብ፡፡

እንደ ተግዳሮት ግን ኤች አይ ቪ ብለህ ስራ ለመስራት ብትነሳ ጀሮ የሚሰጥ የለም ምክንያቱም አዌርነስ ላይ ችግሩ ያለ አይመስልህም ነገር ግን ኢምፕሊመንቴሽኑ ነው ችግሩ፡፡

ሴቶች ላይ የበለጠ እንሰራለን

1. ስራዎችን ስትሰሩ ለየት ያለ የምትከተሉት የአሰራር ስልት፣ ፖሊሲ/ስትራቴጂ ይኖር ይሆን

በስትራቴጂ፣ እቅድ ተነድፎለት ከፍ ባለ ደረጃ ቦታ አልተሰጠውም፡፡ እንዲሁ ይህን እንስራ በሚባል ከፍ ባለ በእቅድ የለንም

ኮንዶም ስርጭት የለም፤ እንደ ዩኒቨርሲቲም አንገብጋቢ ጉዳይ ተገርጎ አልተወሰደውም፡፡ የስራ ሂደቱ አንድ ሰው ይወከላል እንጂ ኤዲስ እንድ አንገብጋቢ ጉዳይ አድርጎ አለመውሰድ፣ ለቫይረሱ ትኩረት አለመስጠት፡፡

ራሱን የቻለ በጀት የለም፣ የሰው ሃይል እጥረትም አለ፡፡ ብዙ ወጪ አያስወጣም በለን የምናስበው አዌርነስስ ክሬሽን ሳይቀር በጀት ይፈልጋል፤፤ ነገር ግን በጀት የለም፡፡

ኤት አይ ቪ አንድ ኢሹ አይነሳም፤ ፕሮጀክትም ሆነ በምርምር ስለ ኤት አይ ቪ ፕሮፖዛል ሲቀርብ አላይም፡፡

1. አገልግሎቱን ፍትሓዊ ተደራሽ ለማድረግ ምን ትሰራላቸው (ድሃ፣ ዝቅተኛ ገቢ ያላቸው፣ ከገጠር የመጡ)

ፍትሓዊ ተደራሽ አለ ብየ አላምንም፡፡ እንደ ስረቪስ ደሊቨሪ ቪክቲም ናቸው ተብሎ ለይቶ መስራት የለም፡፡

እንደሚያውቁት አደርጎ መቁጠርም ይስዋላል፤ ሰዎች እኮ ላያውቁት ይችላሉ፡፡ የተለየ የአሰራር ስልት የለም፡፡ እውቀት፣ አመለካከት አናሳ ነው ብለን የምናስባቸውን ሰዎች ትኩረት ያደረገ አሰራር የለንም፤ በጀት እጥረት ስላለ ለየተን የምንሰራበት ስትራቴጂ የለነም፡፡ እንዲያውም መረጃውን ደጋግሞ የሚሰሙት ሰዎች ላይ ነው ስራዎች ሲሰሩ የሚስተዋሉት፡፡ ሌላው ያን ያክል ትኩረትም እየሰጠነው አደለም፡፡ ጀነሬሽን ጋፕ አንዳይፈጠር እፈራለሁ ምክንያቱም የዛሬ አስር፣ አስራ አምስት አመት ለኤት አይ ቪ የነበረው ትኩረት ሆት ኢሹ ነበር፣ ዛሬ የለም፡፡ አሁን አሁን እየተወለዱ ያሉ ልጆች ስለቫይረሱ ምንም እውቅና የላቸውም፡፡ ለሴክሹዋል ኢንተርኮርስ ሲደርሱ ምንም አይነት የበሽታ መከላከል ሁኔታ ላያደርጉ ይችላሉ፡፡

በፖሊሲ ደረጃ ለእናንተ አለመድረሱ እና ስለፍትሓዊ የአሰራር ስልት መረጃ ያለመኖር አንዱ ችግር ነው፡፡

1. ለምን ይመስልዎታል ሁሉም ተደራሽ ያልሆኑ

ራሱን የቻለ ፋይናንስ የለውም፤ ፋይናንስ ነዳጅ ነው አንድ ነገር ለመስራት፤ ኤት አይ ቪ ቅድሚያ የሚሰጠው አጀንዳ አለመሆኑ.. ነግሌክት ተደርጉዋል፡፡ ቫይረሱ አልጠፋም፣ ሲገድልም እናያለን ገዳይ ነው፡፡ ተደራሽ ያልሆኑ የማህበረሰብ ክፍሎችን ተደራሽ ለማድረግ የሚል አሰራር ስልትም አልተዘረጋም፣ ስራውም አይሰራም፡፡ በፊት መንግስታዊ ያልሆኑ ድርጅቶች ሳይቀር ይመጡ ነበር፤ አሁን ማንም ዞር ብሎ አያውቅም፡፡ ትኩረት ያለመስጠት ጉዳይ ኮሌጁ አሁን ትኩረት የለውም ስለ ኤት አይ ቪ፡፡

ጋፖችን ለይቶ እና መርጦ፣ በጀት መድቦ፣ ትኩረት ሰጦ ስልጠናም አይሰጥም፡፡ ከገጡር ለመጡ፣ ኢኮኖሚ ዝቅተኛ የሆኑ፣ ያልተማሩ ወይም ዝቅተኛ የትም/ት ደረጃ ላይ ያሉትን ያገናዘብ አሰራር የለም፡፡

ወቅታዊ ጉዳዮች ላይ የመጠመድ ሁኔታ ሌላው ተግዳሮት ነው፡፡ መረጃ ክፍተት አለ፤ ምን ያክል የማህረሰብ ክፍል የእኛን ሰራተኛ ጭምር ቫይረሱ በደማቸው አለ ለሚለው መረጃ የለንም፡፡

በጀት፣ ትኩረት ማነስ በሃላፊዎች፣ የሰው ሃይል አለመኖር…

ሜንስትሪም ማድረግ ስለሆነ ተቀናጅቶ ለመስራት እንሞክራለን፤ ተፈጻሚነቱ ግን ችግር አለበት፡፡ ለምሳሌ፡ ቢሮዎች ኤት አይ ቪ የሚመለከት እቅድ እንዲኖራቸው እንሰራለን፡፡

ደም እንዲለግሱ ማድረግ፣ ሞቶዎችን መለጠፍ አልፍ አልፎ አለ፡፡

እንደ አጠቃላ ግን ትኩረት ማጣት ነው፡፡

እንደ ማህበረሰብ ስቲግማ አለ፤ ስለዚህ ኤት አይ ቪ ያለባቸው ሰዎች ራሳቸውን በመደበቅ የመበቀል ነገር እዳለ የምንሰማው ነገር አለ፡፡ ማንነታቸውን ካወቅን እኛ አግኝተን ለመዋያየት እንሞክራለን፡፡

ትኩረት ማነስ እኔ ሳውቀው በየሚዲያው በየከተማው ስለኤት አይ ቪ እንሰማ ነበር አሁን ግን የለም፡፡ ይህ ነገር አሁን የለም፤ እንደ ምሳሌ እሩቅ ሳልሄድ የኔ ልጆች ምንም አያውቁም፡፡ በጣም ትልቅ ክፍተት አለ እንደ አጠቃላይ ያወቀው ኢምፕልመንት ባለማድረግ ያላወቀው ደግሞ ነግሌክት ተደርጉዋል፡፡

ረጅም ጊዜ መኖራቸው እና ሰለ መድሃኒቱ ጥሩነት ብቻ መወራቱም በሽታው ቦታ እንዳይሰጠው ምክንያት ይመስለኛል፡፡

1. ፍትሓዊ አሰራር ለመስራት ምን ቢሰራ ጥሩ ነው ይላሉ

አንብቦ መረዳት የሚችልን ሰው እና ማንበብ የማችልን ሰው በተመሳሳይ አይነት መንገድ ተደራሽ እንሆናለን ለማለት ይከብዳል፤ ስለዚህ ታርጌትድ የሆነ የአሰራር ስልት ያስፈልጋል ብየ አስባለሁ፡፡ ማንበብ አይደለም የሚሰሙትን በተለያየ መንገድ ሊረዱት ስለሚችሉ የስልጠና አሰጣጥ፣ ስትራቴጂክ ፕላን አውጥቶ፣ ያልተማረው በምን አይነት ስትራቴጂ ነው መድረስ የሚቻል የሚል አካሄድ የለም፤ ፍትሃዊ አደለም አሰራሩ፡፡ በራሪ ወረቀትም በለው፣ ድራማም በለው ስቲል ሰም ካይንድ ኦፍ ማህበረሰብ ክፍልን ነው አድሬስ የሚያደርገው እና በመስማት፣ በማየት፣ የሚሰርጹ አስራሮችን መዘርጋት ያስፈልጋል፡፡

በደንብ ትኩረት እንዲሰጠው ማድረግ ያስፈልጋል፡፡ ኤት አይ ቪ አለ እንዴ ብሎ የሚያስብም አለ፡፡

1. ቀሩ የሚሉት ነገር ካለ

በተለይ ጀነሬሽን ጋፕ እንዳይፈጠር መሰራት አለበት፡፡ ኤዲስ አርማው፣ ምልክቱን አሁን እየደረሱ ያሉ ልጆች ላያውቁ ይችላሉ፡፡ ሆስፒታል ላይ ምክር አገልግሎት ስመሰጠቱ እጠራጠራለሁ፡፡ ይህን ምሳሌ ስጠቅስልህ በጦርነቱ ምክንያት ብዙ ቁስለኞች ሆስፒታል ተኝተው ድንገት አንድ ሙያተኛ መድፌ ወጋውና በሽተኛው ደሙ ሲመረመር በሽተኛው ፖዘቲቭ ነበር፡፡ ይህ የሚያሳየው በሽተኛው ቫይረሱ በደሙ መኖሩን አያውቅም ነበር፤ እንግዲህ ይህ ሰው የማህበረሰቡ አንድ አካል ነው፤ አጋጣሚ ሆስፒታል መጥቶ በተፈጠረው ሁኔታ ቫይረሱ እርሱ ጋር መኖሩን ባያውቅ ኑሮ ተመልሶ ሂዶ ሌሎችን መበከሉ አይቀርም ነበር (ሙያተኛው ከዚህ በፊት ቫይረሱ በደሙ መኖሩን ሲጠይቀው እንደማያውቅ ነው የተናገረ)፡፡ ይህ የሚያሳየው ምንም ትኩረት እንዳልተሰጠው ብዙ ሰው እንዲመረመር እንደማይደረግ ነው፤ አደለም ተደራሽ ያልሆኑ ብሎ ለይቶ ስራ ለመስራት፡፡ እና እንደ አዲስ ነገሩን ማነሳሳት ያስፈልጋል፡፡ አንተ ያነሳሃው ጉዳይ በጣም አንገብጋቢ ግን የተረሳ ጉዳይ ስለሆነ ውጤቱን እንጠብቃለን፡፡

**#3**

1. Please tell me about yourself

Post-Graduate Education Level, I am Women, HIV/AIDS and Special needs Director. Worked in this area for 1 year. Age 31 years

2. Tell me the details of the work you are doing

We create awareness among employees and students. We establish and strengthen AIDS Club , Aids day celebration, and we financially support those who have lost their parents (for 21 children) by collecting money from employees.

As a challenge, if you set out to work on HIV, no one will care because I don't think there is a problem with awareness, but the implementation is the problem.

We work more on women.

3. Do you have a special operating strategy, policy/strategy that you follow when doing work?

In a strategy, a plan is not designed and placed at a higher level. So, we don't have a lofty plan to do this. It was not taken lightly as a pressing issue/agenda for the university. Despite one focal person is assigned, not taking AIDS as an urgent issue, not paying attention to the virus.

There is no independent budget, and there is a shortage of manpower. Even Awareness Creation, which we think doesn't cost much, needs a budget. But no budget. HIV is not taken as an issue; I don't see any proposal about HIV either in project or research. No condom distribution.

4. What will you do to make the service accessible to them (poor, low-income, rural)?

I don't believe there is fair and equitable access. There is no identification as a service delivery victim. There is no specific method of operation.

People think as they know about the disease, but they do not know comprehensively. We don't have a system that focuses on people who we think have less knowledge and attitude. There is a lack of budget, so we don't have a unique strategy to work on disadvantaged populations. In contrast, activities are on people who hear the information repeatedly. We are not paying that much attention to the other. I am afraid that there will be a generation gap because the focus on HIV ten, fifteen years ago was a hot issue, it is not today. Children who are being born now have no knowledge of the virus. When they comes to sexual intercourse, they may not have safe sexual practice. One of the problems is the lack of attention at the policy level and the lack of information about equitable service provision procedures.

5. Why do you think they are all inaccessible or inequitable?

It does not have independent financing; Finance is the fuel to do something. The fact that HIV is not a priority agenda, HIV is being ignored. The virus is not gone, we see it killing, it is deadly. A strategy to make inaccessible community sections accessible has not been developed, nor will it work. Before, even non-governmental organizations used to come and involved in HIV services. Now, no one ever turns to work with us. A lack of attention. The college is now not give attention to HIV. There is no planning, training provision, budget allocation, based on gap identification.

There is no system that takes into account those who come from rural areas, have low economic status, are uneducated or have a low level of education. Keeping busy with current issues is another challenge. There is a data gap. We do not have information about how many people, including our employees, have the virus in their blood.

Budget, lack of attention by managers, lack of manpower...

Since it's mainstreaming, we try to work together. But implementation is problematic. For example, we work to ensure that offices have an HIV-specific plan. Getting them to donate blood, posting the motto sometimes... But overall, it's a lack of attention. There is a stigma as a society. So, we hear that people with HIV are retaliating by hiding themselves. If we know who they are, we will try to find them and talk to them.

Lack of Attention… When I was aware, we used to hear about HIV in the media in every city, but now it is not. This thing no longer exists. As an example, my kids don't know anything. There is a very big gap, in general, those who has knowledge cannot implement, and those who do not know it, ignored, and neglected it.

I think that the fact that they exist for a long time and that the goodness of the medicine is only talked about is the reason why the disease is not given a place.

6. What is your suggestion to provide equitable HIV/AIDS services?

It is difficult to say that we will reach the literate and the illiterate in the same way; So, I think a targeted and tailored strategy is needed. It is not only by reading but also, they can understand what they hear in different ways. The current procedure is not fair.

Be it a pamphlet or a drama, Still Some Kind of Society addresses a section of society, and it is necessary to lay down the tenets that can be written by hearing, seeing.

It needs to be well focused. Some wonder if there is HIV.

7.If there is something left to say

In particular, it should be done to prevent generation gap. HIV/AIDS' logo and the sign may not be recognized by children who are reaching it now. I doubt that proper counseling is provided at the hospital. I will mention this example to you because of the war, many wounded were lying in the hospital and suddenly a professional shot a cannon and the patient's blood was tested and the patient was positive. This suggests that the patient was unaware of the presence of the virus in his blood. So, this person is a part of the community. In the event that he came to the hospital and did not know that the virus was with him, he would have gone back and infected others (he said that he did not know if the virus was present in his blood before). This shows that no attention has been paid to the fact that many people are not being investigated. Not to do work that is identified as inaccessible. And it needs to be stirred up HIV as anew agenda. The issue you have raised is a very urgent but forgotten issue, so we await the results.

**#4**

1. ኤች አይ ቪ ቫይረስን በተመለከተ ያለብህን ኃላፊነት ብትነግረኝ

ፎካል ፐርሰን ነኝ፡፡ ቢሮው የተደራጀ ጸረ-ኤች አይ ቪ ክለብ አለው፡፡ ሰራኛው ከደሞዙ አንድ ፐርሰነት በየወሩ ያዋጣል፡፡ እኛ ስንጀምር የነበረው ካፒታል ወደ 32 ሺህ ብር ነው የነበረው አሁን 180 ሺህ ብር ደርሱዋል፡፡ የምግዛቸው ልጆች አሉ፡፡ በአጠቃላይ ስድስት ልጆች ሁለት ወንድና አራት ሴቶች እናግዛለን፡፡ ግንዛቤ እናስጨብጣለን፣ የኤች አይ ቪ ኤዲስ ቀን እናከብለን፡፡ ወደ 240 የሚሆኑ ሰራኞች 1 ፐርሰንት እየከፈሉ ነው ያሉት፡፡ ለልጆች አሳዳጊዎች እንደ እየ ሓይማኖታቸው ባዕል ሲሆን ገንዘብ ይሰጣቸዋል፡፡ ቫይረሱ በደማቸው ያለባቸው ሰራተኞችን አልፎ አልፎ በገንዘብ እናግዛቸዋለን፤ በተለይ ታመው ከታከሙ ሪሲት አምጥተው እናወራርድላቸዋለን፡፡ ሰብጀክት ማተር ስፔሻሊስት የሚባሉ ሙያተኞችም አሉን አብረው ስራውን እንዲገመግሙ ይደረጋል፡፡ ኮንዶም ስርጭት ይሰራል፡፡ ከዚህ በፊት ጤና ቢሮው ነበር የሚሸፍን አሁን ግን በራሳችሁ ስሩ የሚባል ነገር አለ፡፡ የኤች አይቪን ጉዳይ እየገደለው ያለ የጤና ሴክተር ይመሰልኛል፡፡ በልጽገናል ብለን መሰለኝ እርዳታም የለም፣ ድጋፍም የለም፡፡ እኛ ግን ሲስተሙ የገባን ስለሆነ ሪፖርት በመላክ ደረጃ አንድ ጊዜም ተቁዋርጦ አያውቅም፡፡ ስራው ቢሰራም ባይሰራም ሪፖርት ይደረጋል፡፡ የኤች አይ ቪ ጉዳይም በመስክ ስምሪት ጊዜም ይሰጣል፡፡ ምናልባት ግን ያን ያክል ሲሪየስ ነው ማለት አይቻልም፡፡ ምናልባት የሚሄደው ባለሙያ ሲፈልግ ብቻ የሚሰራው ነው የሚሆነው፡፡ በደንብ ተደርጎ አይገመገምም፤ ለምሳሌ እንደ በጋ መስኖ አሰራር ተደርጎ ላይገመገም ይችላል፡፡ ቢያንስ ግን በአቴንሽን ደረጃ በዚያ ልክ እየተደረገ ነው፡፡የግብርና ቢሮ አሁን በያዝነው አመት የአመታዊ የሚዲያ ውሎች አሉን፤ ከፋናም፣ ከአማራ ቴሌቪዝንና ሬዲዮም ጋር ውል አለን፡፡ በውላችን መሰረት በየሁለት ሳምንቱ የ20 ወይም 30 ደቂቃ ፕሮግራም አለ በዚያ ውስጥ የግብርና ጉዳይ እንደሚወራው ሁሉ፣ የወጣቶች ተሳታፊነት፣ የስራ ፈጠራ፣ የኤች አይ ቪ ኤዲስ ጉዳይ ሁሉ ይነሳሉ፡፡ በብዛት የገጠሩን ማህረሰብ ለመድረስ በሬዲዮ እንጠቀማለን፡፡ በባነሮች ትኩረት ለኤች አይ ቪ እያልን እውቅና እንለጥፋለን፡፡ በራሪ ወረቀት መጠቀም እንኩዋን እየቀነሰ ነው፡፡ ከጤና ቢሮ ተጨማሪ ልጆች የምግዛቸው እንዲሰጡን ተነጋግረናል፡፡

ያጋጠመን ችግር ኮሮና ሲከሰት ትኩረቱ ሁሉ ወደኮረና መሆኑ ነበር፡፡ ሌላው የአለም አቀፍም ሆነ የአገራችን የጤናው ሴክተር ለኤች አይቪ የሚሰጠው ሽፋን እጅግ አነስተኛ መሆኑ እና አሁን ግን ሕዝብ እያለቀ ነው ማለት ይቻላል፡፡ በአንዳንድ ከተሞች የሚሰሙ ጭምጭምታዎች ኮንዶም እንኩዋን 50 ብር… አምሳ ብር ገዝቶ የሚጠቀም ምን አይነት ገበሬ ነው፡፡ እንዴት አይነት የቀን ሰራተኛ ነው ሀያ ብር ሰላሳ ብር ገዝቶ የሚጠቀም ዝም ብሎ ተጠቅሞ ይሄዳል እንጂ፡፡

1. የኤች አይ ቪን መከላከል መሰረት ተደርጎ የምትሰሩት ስራ ምን ያክል ታች መዋቅራዊ ነው

እስከ ታች ወረዳ የሚደርስ መዋቅራዊ አሰራር የለውም፡፡ የሰው ችግርም አለ፡፡ ለምሳሌ የሴቶች ጉዳይ፣ የስራ እድል ፈጠራ እና የኤች አይ ቪ ጉዳይን የምትመራ ኤክስቴንሽን ኮሚኒኬሽን የተወከለች አለች፡፡ ሶስቱም ከበባድ ስራዎች እንዴት አንድ ሰው ሊሰራው ይችላል፡፡ ቤቱ ራሱ ይመልሰው፡፡

1. ሁሉን ማህበረሰብ ክፍል ለመድረስ ምን ታደርላችሁ ከላይ ከጠቀስኃቸው በተጨማሪ

ምንም እንኩዋን ጦርነቱ ኮረናው ትኩረቱን ቢወስዱትም በሬዲዮም ተደራሽ ለመሆን እንሰራለን፡፡ ለሁሉም ግን ተደራሽ ነው ማለት አይቻልም፡፡ ምክንያቱም ሚዲያ የማይከታተለው፣ ሬዲዮም የሌለው ይኖራል፡፡ እንዲሁም ድኃው ከየት አምጥቶ ነው ኮንዶም በውድ ዋጋ ገዝቶ የሚጠቀም፡፡

1. የኤች አይ ቪ ኤዲስ አገልግሎት ለሁሉም ተደራሽ እንዳታደርጉ የሚደርጉ ተጨማሪ እንከን ካለ ብትነግረኝ

ግብርና ቢሮው ዋና ተቀዳሚ ስራው ምርታማነትን መጨመር ነው፡፡ ኤች አይ ቪ ጉዳይ ግን ሁለተኛ ወይም ተካታች ነው፡፡ በፊት የነበሩት ድርጅቶች የሉም አሁን፡፡ አሁን አሁን ስብሰባ የለም፣ መመካከር የለም፣ በአመራሩ ጋር አጀንዳ አድርጎ ማውራት የለም፡፡ ዋናው ተግዳሮት አቴንሽን ነው፤ የበጋ መስኖ ስንዴ በለው፡፡ የበጋ መስኖ ስንዴ በአማራ ሰማይ ስር መሬት አለ፣ ውሃ አለ፣ ሁሉ ነገር ነበር ከዚህ በፊት ያልበር አሁን ግን የትኛውም ቦታ ምርት እየተመረተ ነው፡፡ ለምን ቢባል የመስኖ ስንዴ ፖለቲሳይዝ ስለተደረገ ነው፤ ሌላ ምንም ምክንያት የለም፡፡ ይህ ባህል ሁኑዋል፡፡ ወይንም ደግሞ ጂያንት የሆነ ጤና ሴክተር ቢኖር ኑሮ ጤና ቢሮው ብቻውን ይሰራው ነበር፡፡ ገብቶሃል… ጤናው ቢሮው ከእኛ ጋርም ተቀናጅቶ መስራት አለበት፤ ተቀናጅቶ የመስራት ሁኔታው ደካማ ነው፡፡ ግልጽ የሆነ ጥርት ያለ ተረዳድቶና ተባብሮ የመስራት ሂደቱ በግለሰቦች መወዳጀት የተመሰረተ እንጂ መዋቅሩም ደካማ ነው፡፡ ተቁዋም አልገነባንም፡፡ ተቁዋም ስትገነባ ሰራህም አልሰራህም ሲስተሙ ይጠይቅሃል፤ አሁን ግን እኔ ብሰራ ባልሰራ ማንም አይጠይቀኝም፤ በእኔ ፍላጎት ላይ ብቻ የተመሰረተ ነው፡፡ ተቁዋማዊ አስተሳሰብ ያስፈልጋል፡፡ ጤና ቢሮው ትታል፤ ስልጠና መስጠት ከጀመረ ቆየ፡፡ ለኤች አቪም ባለሙያ ይመደብ ራሱን የቻለ፡፡ አንድ ሰው ሁለት ሶስት ስራ ደርቦ እንደት ይሰራል፡፡

ቴክኖሎጂው የራሱን አስተዋጽኦ ያደርጋል፡፡ ገጠሩ የት ያገኛል ቴክሎጂ፡፡

1. ተጨማሪ የምትነግረኝ ካለ

ቀበጥሁ የሚለው፣ የሚቀብጥ ሚጠጣ ያለው ነው እና ተጠቂ ነው፡፡የተማረውና ሐብታሙ ተጠቂ ሆነ ማለት አደገኛ ነው ለማህበረሰቡም፡፡ እና ፖሊሲ የሚያስቀይር ምርምር ተደርጎ የኤች አይ ቪ ጉዳይ ቀጣይነት ያለው አሰራር እንዲኖር አነጋጋሪ አጀንዳ ማድረግ ያስፈልጋል፡፡

**#4**

1. Tell me about your responsibility regarding the HIV virus

I am a focal person. The office has an organized anti-HIV club. The employee contributes one percent of his salary every month. When we started, the capital was about 32 thousand birr, now it has reached 180 thousand birr. There are children I buy. In total, we help six children, two boys and four girls. We raise awareness, celebrate HIV AIDS Day. About 240 workers are paying 1 percent. Children's guardians are given money according to their religion.

We occasionally financially support workers who have the virus in their blood; Especially if they are sick and treated, we will urge them to bring a receipt. We also have professionals called subject matter specialists who will be assigned to evaluate the work together. Condom distribution works. Before, it was covered by the health office, but now there is something called self-sponsored. I think the health sector is killing the HIV issue. I thought we were getting betteroff economically and there was no help or support. But since we are logged into the system, there has never been a single change in the level of sending a report. It will be reported whether the job works or not. The issue of HIV is also given during field deployment. But maybe it's not that serious. Maybe it's the only thing he does when he needs a professional to go. It is not well evaluated. For example, it may not be evaluated as a summer irrigation system. At least, it gets attention to this level. The Agriculture Bureau now has annual media contracts in the current year. We have a contract with Fana, Amhara Television and Radio. According to our contract, every two weeks there is a 20- or 30-minute program in which the issue of agriculture, youth participation, entrepreneurship, HIV and AIDS are discussed. We use radio to reach the mostly rural community. With banners we will post recognition saying Attention to HIV. Leaflet use is decreasing. We talked to the health office to give us more children to grow them.

The problem we faced was that when Corona happened, all the attention was on Corona. Another problem is that the health sector, both international and national, provides very little coverage for HIV and now I can say the population is infected. Rumors heard in some cities are: What kind of farmer buys condoms for 50 birr...50 birr and uses them. What kind of day laborer is he who buys twenty birr and thirty birr and uses it, but just goes away using it.

2. How structural is the work you do as a basis for HIV prevention?

It does not have a structural system that reaches down to the district. There is also a human power problem. For example, a woman is assigned by Extension Communication, which handles women's issues, job creation and HIV issues. How can one person do all three difficult tasks? Let the house itself answer it back.
3. In addition to what you mentioned above, what will you do to reach every part of society? Even if the war takes away the attention, we will work to be accessible on the radio. But it cannot be said that it is accessible to all. Because there is no one who doesn't follow the media and doesn't have a radio. Also, where did the poor man buy and use condoms at an expensive price?

4. Tell me if there are any additional challenges that prevent HIV AIDS services from being accessible to all

The main priority of the Agriculture Bureau is to increase agricultural productivity. HIV is a secondary or inclusive issue. The former organizations no longer exist. Now, there is no meeting, no consultation, no talking with the leadership as an agenda. The main challenge is attention, which is like summer irrigated wheat. Summer irrigated wheat is under the Amhara sky, there is land, there is water, there was everything that was not flown before but now it is being produced everywhere because irrigated wheat is politicized, there is no other reason. This becomes a culture.

Or if there was a giant health sector, the living health office would work alone. You understand... the health office must work together with us; Coordination is weak. The process of clear understanding and cooperation is based on personal friendship, but the structure is weak. We did not build the institution.

When you build an institution one, the system will ask you whether you have done it or not. But now, if I work, no one will ask me if I don't work. It just depends on my preference. Critical thinking is required. The health office left it, training provision stopped now. Independently assigned to HIV specialist is required. How a person works two or three jobs. Technology plays a role, but how does the countryside find technology?

5. If you have more, please to tell me

The one who says that he is drunk is someone who drinks and drinks and is a victim. It is dangerous for the society to say that the educated and the wealthy have become victims. And policy-changing research needs to be done to make the HIV issue a sustainable agenda.

**#5**

1. እባክሽ ስለኤች አይቪ ያንቺን ኃላፊነትና የስራ ድርሻሽን ንገሪኝ

ስለኤች አይቪ ያለብኝ ኃላፊነት ስልጠና እንዲሰጥ ማስተባበር፣ ኮንዶም ስርጭት ላይ እንሰራ ነበር፡፡ አሁን አሁን ግን ትኩረቱ የለም፤ ስንጠይቅ በጀት የለም ነው የሚሉህ፡፡ በመስሪያ ቤት 2 ፐርሰነት በጀት ይመደባል፡፡ አሰራሩ ዞንና ወረዳ ድረስ ወርደን እንድንሰራ ነው፡፡ ነገር ግን በጀቱ በክልል ደረጃ ያለውን ስራ የሚሽፍን በጀት የለም፡፡

1. ተደራሽ ያልሆኑ የማህበረሰብ ክፍልን ለይቶ የመድረስ ስራ ምን ይመስላል

አዎ ሁሉም ሰው የአገልግሎቱ ተጠቃሚ አይደለም፡፡ በተለይ አሁን ያለው ጀነሬሽን በሚገባው ልክ ኤች አይ ቪን ያውቃል ማለት ይከብዳል፡፡ በፊት የነበረውን ሁኔታ ሁላችንም የበሽታውን አሰቃቂነት እናስታውሳለን እናውቃን፡፡ አሁን ግን ከቫይረሱ ጋር የሚኖሩ ሰዎች መድኃኒት ስለሚወስዱና ጠባሳ ስለሌለባቸው ትውልዱ ላይ ካልተሰራ አያውቁትም፡፡ ይህ ደግሞ የሁላችንም ኃላፊነት ነው፡፡ እኔ ግን የመንግስት ጉዳየ ብሎ መነጋገሪያ አለማድረግ ነው ብየ አምናለሁ፡፡ መንግስት ግን ዝምታው ምንድን ነው… ኤች አይ ቪ ጠፋ ብሎ ነው ወይስ ምንድን ነው ብየ ስብሰባ ላይ ሁሉ ጠይቄ አውቃለሁ፤ ነውስ ወይስ ባሕታዊ ናቸው ጾታዊ ግንኙነት አያደርጉም ተብሎ ነው ወይስ ምንድን ነው ዝም የተባለ ብየ ስብሰባ ላይ ጠይቄ አውቃለሁ፡፡የመንግስት ትኩረት አለማድረግ ችግር አለ፡፡

1. ተጨማሪ ፍትሐዊ ተደራሽ እንዳይሆን እንቅፋት የሆነባችሁ ነገር ምንድን ነው

ትኩረት ባለመስጠ፣ ፖሊሲ ባለማውጣት፣ ህዝቡ እያለቀ ዝም ማለት ያሳስበኛል፡፡ የበጀት ችግር ሌላው ነው፡፡ እና ደግሞ አንዳንድ ግዜ ግልጽ የሆነ የአሰራር ስልት ያለመኖር፡፡ በእርግጥ ጤና ቢሮ በዓመት ሁለት ጊዜ ስልጠና ይሰጣል፡፡ በእኛ በኩል የሰው ኃይል እጥረት የለም፡፡ ኮንዶም እጥረት ነው ያለ፡፡ ከዚህ በፊት ኮንዶም ስርጭት በደንብ እንሰራ ነበር፤ አሁን ግን እጥረት ስላለ ማሰራጨት አልቻልንም፡፡ ሆስፒታል ጠየቅሁ፣ ጤና ቢሮ ጠየቅሁ የላቸውም፡፡ ተቀናጅታችሁ ስሩ ተቀናጅታችሁ ስሩ ይባልል ግን ተግባር ላይ አታገኘውም፡፡ ከስምንት በላይ የስራ ሂደት አለ ነገር ግን ተቀናጅቶ የመስራት ነገር የለም፡፡ ከበጀቱም፣ ከመንግስት ትኩረት አንጻር በዘላቂነት ስራዎችን ዳር የማድረስ ችግር ይስተዋላል፡፡ የአጀንዳዎች መፈራረቅ እና ተለዋዋጭነት ስለዚህ በሽታ ትኩረት እንዲነፈገው ሆነ ፡፡ ጦርነቱ፣ ብጥብጡ ሁሉንም የማህበረሰብ ክፍል ስለመሚመለከት በሰዓቱ ሊያጠፋህ ስለሆነ ችግር እንጂ ሰለ ኤች አይ ቪ ማን ትዝ ይለዋል ብለህ ነው፡፡ የምናውቃቸው ወታደሮች፣ ጦር ሜዳ የሆዱ ሰዎች በቀላሉ ተጋላጭ ናቸው፤ እነሱም ሌላ ሰው ይበክላሉ፡፡

1. ተጨማሪ ቀረ የምትይው ተግዳሮት ካለ እባክሽ

እስከ ዞን ወረዳ ውረዱ ይባላል እንጂ ያንን የሚመጥን በጀት የለም፡፡ መደቡን ፈቅደው ቁጭ አደረጉት እንጂ ተፈጻሚነቱንና ተግባራዊነቱን የሚከታተል ኃይል የለም፡፡

1. ወደ ፊት ቢሆን ወይም ቢስተካከል ጥሩ ነው የምትይው ካለሽ

እኛ እሳችን በቂና የአሰልጣኝ ስልጠና ወስደን በነቃ ተሳትፎ ተደራሽ ላልሆኑት ማህረሰብ ክፍል ብናደርስ እላለሁ፡፡ ዞን፣ ቀበሌ ላይ ወርደን ብናስተምር፡፡ ምንግስትም ትኩረት ቢሰጠው መልካም ነው፡፡

1. በመጨረሻም የምትይው ነገር ካለሽ

የኸው ነው፤ የምለው የለኝም፡፡

**#5**

1. Please tell me your responsibility and role regarding HIV

I was responsible for coordinating training on HIV and distributing condoms. But now there is not attention given to HIV. When we ask, they say there is no budget. 2 percent of the office budget is allocated to HIV. The procedure is for us to go down to the zone and district, but the budget does not even cover the work at the regional level alone.

2. How you reach community members who do not get services?

Yes, not everyone is a user of the service. It is difficult to say that the current generation, in particular, knows HIV as well as it should. We all remember the horrors of the disease before. But now people living with the virus take medication and don't have scars, so this generation don't know if more work is not done for this generation. And this is the responsibility of all of us. But I believe that it is a matter of the government which should make a talk of agenda. But why is the silence of the government?... Do we think this generation do not perform sex…, or why it is be silent for this virus? I do ask this usually in meeting. There is a problem of lack of government attention.

3. What is the obstacle for you to deliver more fair services?

I am concerned about not paying attention, not making a policy, and being silent while people are dying. The budget problem is another. And sometimes not having a clear strategy. In fact, the Health Bureau provides training twice a year. There is no shortage of manpower on our side. There is a shortage of condoms. We used to do a good job of distributing condoms. But now we are unable to distribute it because there is a shortage. I asked the hospital, I asked the health office, but they didn't have it. It's called the joint root, but you won't find it in action. They called to work in collaboration, it is said work in collaboration, but you won't find it in practice. There are more than eight workflows but nothing to work together. Due to the problem of budget and the government's attention, the problem of sustaining projects can be seen. The alternation and volatility of agendas has led to this disease being deprived of attention. The war, the violence affects all sections of the society, and we focus on the issues which is going to kill you presently, so who no one remember HIV but it is a problem. There are soldiers I know, people who have been on the battlefield are vulnerable; They infect other people.

4. If there is additional challenge you want to say, please

It is recommended going down to the zone district, but there is no budget that fits that. There is also no force to monitor its implementation and practicality.

5. What should do in the future to solve these challenges?

I would like to say that if we take enough training of trainers and reach out to the unreached sections of the society through active participation. It will better if we go down to Keble and reach them. It is good if the government pays attention to it.

6. If you have something to add, please

All are this. I have nothing to say.

**#6**

1. ስለ ኤች አይ ቪ ጉዳይ ያለብሽ ኃላፊነት

በመንግስት ኮሚኒኬሽን ጉዳዮች ቢሮ የሚዲያ ልማት ባለሙያ እና የኤች አይ ቪ ፎካል ነኝ፡፡ ከጤና ቢሮ የሚወርዱ መመሪያዎችና የሚኒስትሪንግ ተግባራት አካቶ መስራት ነው፡፡

ይህን ለመስራት የራሱ ችግር አለበት፡፡ እንደ አገር አቀፍ ደረጃ ስለ ቫይረሱ እየተፋዘዘ ቢመጣም በመስሪያ ቤቴ ግንዛቤ ፈጠራ ላይ እሰራለሁ፡፡ ግንዛቤ በመፍጠር በኩል የምከተለው መንገድ ተመሳሳይ ስለሆነ ሰው ቸልተኛ መሆንና ሰው ያወቁ ቢመስለውም አለመዋቃቸውን በደንብ እዲረዱ እንሞክራለን፡፡ በቫይሰቡ ቤተሰቦቻቸውን ያጡ ልጆችን እንረዳለን፡፡ በህመት ሚዲያ በሚሰሩ ስራዎች በቲ-ሸርት፣ በራሪ ወረቀት ግንዛቤ እንዲፈጠር እናደርጋለን፡፡

ደንበኞችን ያማከለ ስራ ደግሞ መረጃ ዴስክ ላይ በራሪ ወረቀት በማስቀመጥ እና ኮንዶም አንዳንድ ቦታዎች ላይ በማስቀመጥ እንዲወስዱ እናደርጋለን፡፡

1. ያልተሰሩ ስራዎች አሉ ብለሽ ካሰብሽ ብትነግሪኝ

አንደ አገር እየተረሳ ነው፡፡ ሰው ስሚዘነጋው አስተምህሮትን መቀየርና ቀጣይነት ያለው ስራ መስራት ያስፈልጋል፡፡ እውነቴን ነው ይህ ኤች አይ ቪ አስቸኩዋይ ጊዜ አዋጅ የሚባለው ራሱ ያስፈልገዋል፡፡ እኔ ልጆች አሉኝ፤ ለልጆቼ እፈራለሁ፡፡ ከቫይሱ ነጻ የሆነ ሀገር ተፈጥሮ ማየት ቀላል ነገር አይደለም፡፡ እየሆነ ነገር ያለውን ስታይ እየተሰራ ያለው ስራ ምንም ነው ማለት ትችላለህ፡፡ ዛሬ የቴክኖሎጂ ዘመን ነው፣ ልጆችን ምንም ነገር መከልከል አትችልም፣ እንደ ልባቸው ነው ሁሉን ነገር የሚያዩት፣ የማያውቁት የማያዩት ነገር አይኖርም፡፡ እና ደመነፍሳዊ የእድሜ ክልል ላይ ሁሉን ነገር መንካት የሚፈልጉበት ዘመን ስለሆነ እኔ በዚያ ልክ ያስፈራኛል፡፡ በፊት በተቁዋም ደረጃ ተደራጅቶ የራሱ መስሪያ ቤት ነበረው፣ በክልል ደረጃ ኤች አይ ቪ ሴክሪታሪያት የሚባል ነገር ነበረው፡፡ አሁን ግን ያ ሁሉ ተቀይሮ በጤና ቢሮ ውስጥ ዳይሬክቶሬት ተብላ እንደ አንድ የስራ ሂደት ነው ያለችው ማለት ነው፡፡ ያ ሁሉ የሚያሳየው ለበሽታው ትኩረት አለመስጠት ነው፡፡ እኔ ከየትኛውም በሽታ ይልቅ ትውልድ እያሳጣን ነው ብየ ነው የማስበው፡፡

እንደ ተቁዋም ስትመጣ ምቹ አይደለም፡፡ ማለት ፎካል ፐርሰን ነው፡፡ አንዳንድ መስሪያ ቤቶች አሉ ለምሳሌ ትም/ት ቢሮ፣ ሲቪል ሰርቪስ በመደብ ደረጃ መዋቅር ያላቸው ደሞዝ የሚከፈላቸው መኖሩ፡፡ ነገር ግን አደረጃጀቱን ዝቅ ማድረጉ መደቡ ጭምር ነው እየጠፋ የሚሄደው፡፡ እና የስራ ሂደት ቢኖረው፣ አደረጃጀቱ ከታች ድረስ ቢወርድ፣ መዋቅሩ ቢሰፋ ተጠየቂያዊ ስራ ይሰራል፡፡

1. ፍትሓዊ ተደራሽ የሆነ ስራ እንዳንሰራ ተግዳሮት ካሉ ብትጠቅሽልኝ

የመጀመሪያው መንግስት ትኩረት አለማድግ ነው፡፡ ራስን በራስ ለመደገፍ የኤዲስ ፈንድ ማቅዋቁዋምና ጥሩ ተሞክሮዎችን ማስፋት፣ አገር በቀል መንግስታዊ ያልሆኑ ተቁዋማት እንዲኖሩ ማድረግ አስፈላጊ ነው፡፡

ሌላኛው መንግስት ቫይረሱን ቀንሸዋለሁ ስላለ ነው መሰል አጋዥ ድርጅቶች እየጠፉ መጡ፡፡ ሌላኛው ባህላችን ቴክኖሎጂው ጥሩ ነገር ቢኖረውም ጎጂ ነገርም አለው፡፡ ህብረተሰቡ የባህል አብዮት ማምጣት አለበት፡፡ ከልጆች ጋር በግልጽ ጾታዊ ሁኔታዎችን መወያየትና መነጋገር መቻል ያስፈልጋል፡፡

ለምሳሌ ከዚህ ስንወያይ ባልሽ ፊልድ ሲሄድ ኮንዶም ታደርለታለሽ የሚል ሐሳብ ብታነሳ በስመአቭ ወወልድ ወመንፈስ ቅዱስ! ነው የሚሉህ፣ ከሴት ጋር ብትይዢውስ ብትለው እንዲሁ በስመአቭ ወወልድ ወመንፈስ ቅዱስ! ነው የሚሉህ፡፡ በግልጽ የመወያየትና የመነጋገር ችግር አለ፡፡

ፍትሓዊና ተደራሽነት በሚል መሪ ቃል ዘንድሮ አክብረናል፡፡ ነገር ግን አገልግሎቱ ፍትሐዊ ነው ለማለት ይቸግረኛል፡፡ ሰው ራሱን ለማወቅ ተቸግሮ ይመጣል እንጂ ከአገልግሎቱ የራቁ ሰዎችን የምንደረስበት ዘዴ የለንም፡፡

አንዳንድ ጊዜ ኪት አጠረ ሲሉ እሰማለሁ፡፡

ተከታታይት ያለው ስልጠና ስለፍትሓዊ አገልግሎት አሰጣጥም የለም፡፡ እሱም ሌላኛው ችግር ነው፡፡በጥቅሉ ኤች አይ ቪ የተፋዘዘ ስለሆነ ተቀናጅቶ መስራትም ሆነ ተጠያቂነት የሌለበት ስራ እየሖነ ነው፡፡ አሁን አሁን ደግሞ ወቅቱ በራሱ ችግር እየፈጠረብን ነው፤ ቀውስ ወቅት ጊዜ መረጃ መለዋወጥም ሆነ አገልግሎቱ ላይ ትኩረት አድርገን መስራት ተቸግረናል፡፡

1. ፍትሓዊ አገልግሎት ለመስጠት ምን ቢደረግ ጥሩ ነው ትያለሽ

መዋቅራዊ አደረጃጀቱን ማስፋት፣ በቢሮ ደረጃ እራሱን የቻለ ቢሆንና ከሁሉም የማህበረሰብ ክፍል ጋር ተቀናጅቶ መስራት ጥሩ ነው፡፡ ከሐይማኖት አባቶች ጋር፣ እና ከሌሎች ድርጅቶች ጋር መስራት የስፈልጋል፡፡ ሞት ቢቀንስም ስርጭት ግን አልቀነሰም እና ብዙ መስራት ያስፈልጋል፡፡

1. በመጨረሻም የምትናገሪው ነገር ካለሽ

ከላይ እንደዘረዘርሁት ነው፤ የተለየ ነገር የለኝም፡፡

**#6**

1. Please tell me about Your responsibility regarding HIV

I am a media development specialist and HIV focal in the Office of Government Communications. It is working together with instructions from the health office and ministerial duties. Doing this has its own problems. I am working on creating awareness in my office even though the virus is ignored nationwide. Because the way or approach I follow through creating awareness is the same, people boring on it. We help children who have lost their families. We will create awareness through t-shirts, flyers through the activities of printed Media. Customer-oriented work is done by placing flyers at the information desk and placing condoms in some places.

1. Tell me if you think there are any unfinished works

A country is seeming forget HIV. When people forget it, it is necessary to change the doctrine and do continuous work. The truth is this HIV crisis needs its own declaration. I have children. I fear for my children. It is not easy to see the virus-free country. When you see what is happening, you can say that the work being done is nothing. Today is the age of technology, you can't deny children anything, they see everything according to their hearts, there is nothing they don't see that they don't know. And because it's an age where you want to touch everything at an impulsive age, I'm just scared of that. Previously, it was organized at the national level and had its own office, at the regional level it had something called the HIV Secretariat. But now all that has changed, and it is called a directorate in the health office as a minor work process. All that shows is the lack of attention to the disease. I think we are losing a generation more than any other disease. It is not comfortable when you come as to my sector, It means focal person. There are some offices such as the Education Bureau, Civil Service that have a classic structure and are paid salaries. But lowering the organizational level means that the position or office is also disappearing. And if it has a work process, if the organizational structure goes down to the bottom, if the structure expands, it will work based on accountably and responsibly.

1. If there are any challenges to do fair and accessible work, please let me know

The first, government does not give attention. For self-support, it is important to organize an AIDS fund and spread good practices, and make indigenous non-governmental organizations exist. Second, aid organizations are disappearing because the government said it reduced the virus. Another, based on our culture, technology has both good and bad things. Society must bring about a cultural revolution. It is important to be able to openly discuss and discuss sexual situations with children. For example, if you bring up the idea that when your husband goes to the field, you will give him a condom. They say to you, if you marry a woman, what if you say it is the same with the Holy Spirit! They say it is.

There is a problem of open discussion and communication.

This year we celebrated with the theme of equity. But I find it difficult to say that the service is fair. We don't have a way to reach people who are far away from the service, but people come with difficulty to know themselves. Sometimes I hear that there is shortage of resources, like test kit.

There is no consistent training on equitable service delivery. This is another problem. In general, because HIV is spread, there is no coordination or accountability. Now and now the current condition (conflict or political tension) itself is creating problems for us. During the crisis, it is difficult for us to focus on the information exchange and the service.

What should be done to provide fair service?

It is better to expand the structural organization, whether it is autonomous at the office level and working in coordination with all parts of society. I need to work with religious leaders, and with other organizations. Although deaths have decreased, transmission has not and more needs to be done.

Finally, if you have something to say, please…

As I have listed above. I have nothing special.

**#7**

1. ሃላፊነትህና ስለምትሰራው ስራ ብትነግረኝ

ሜኒስትሪሚኒግ አድርገን ነው የምንሰራ፡፡ ፍትኃዊነት ስተባለው ጉዳይ ተደራሽቱ እስከ ዞንና ወረዳ ድረስ እናወርደዋለን፡፡ በተቁዋማችን ደግሞ በየሩብ አመቱ ግዛቤ የማስጨበጫ ስራ እንሰራለን፡፡ እንዲሁም ወላጅ አልባ ህጻናትን እንረዳለን፡፡ ሰራተኛው ከደሞዙ እየቆረጠ እነዚህን ልጆች ያሳድጋል፡፡ ፍትሓዊ ተደራሽ ስንል ለምሳሌ የኛ መስሪያ ቤት ደንበኞች ባለ ሃብቶች ከሩቅ መጥተው ጉዳይ ለማስፈጽም ያድራሉ፣ በዚህን ጊዜ ሌላ ሰው ጋር ግንኙነት ሊያደርጉ ይችላሉ፡፡ ስለዚህ ኮንዶም ያስፈልጋቸዋል፡፡ ነገር ግ ኮንዶም አቅርቦት የለም፡፡ ኮንዶም ከመስሪያ ቤታችን ለሚፈልግ ቢታደል በተዘዋዋሪ በሽታው እየተስፋፋ እንደሆነ መልዕክት ማስተላለፊያ ዘዴም ስለሆነ ሰው ንቁ ይሆናል፡፡ በሽታው በጣም እየተረሳ ያለበት ሁኔታ ላይ ነው፡፡

1. ትኩረት አድርጋችሁ የምትሰሩባቸው የማህበረሰብ ክፍል ካሉ ብትነግረኝ

ትኩረት አድርገን ከምንሰራባቸው መካከል የአበባ ክፍል አለ፤ እናም ሴቶች በብዛት የሚቀጠሩበት ኢንዱስትሪ ስለሆነ በትኩረት እንሰራበታለን፡፡

1. ፍትሓዊ ተደራሽ ለማድረግ እንደ ተግዳሮት የምትጠቅስልኝ ነገር ካለ

ለምሳሌ በመስሪያ ቤታችን 106 ሰራተኞች አሉን እና በደማቸው ቫይረሱ ያለባቸውን ሙሉዎችን መርዳት አልቻልንም፡፡ ደሞዝ አነስተኛ ለሆነ እናግዛለን፡፡ ስለዚህ የፋይናንስ እጥረትም ስላለ ሁሉን መድረስ አንችልም፡፡ ይህንንም የምናደርገው በሰራተኛው መልካም ፈቃድ ከ100 ብር 50 ሳንቲም እየሰጠ ነው እያገዝን ያለነው፡፡

15ቱ ዞኖች ላይ ፍትሓዊ አድርጎ መስራት የማንችልበት ምክንያት ለምሳሌ ጦርነቱ የነበረበት አካባቢ ሴቶች ተደፍረዋል፤ ኮንዶም ስርጭት፣ ግንዛቤ ማስጨበጫ ስራ ያስፈልግ ነበር ግን አልተሰራም፡፡ ሌላው ትኩረት ማነስ፣ የፋይናንስ ችግር፣ ተቀናጅቶ መሰራት ያለመቻል ተግዳሮት ናቸው፡፡ ከጤና ቢሮው ጋር ያለን ግንኙት የላላ ነው፡፡ የትራንስፖረት ችግርም አለ፡፡ ትራንስፖርት አስፈቅዶ ወደ ሚፈለገው አካባቢ ለመድረስ ያዳግተናል፡፡ ሪፖርት መላላክ ላያ ያተኮረ ነው፡፡ ከሁሉም ቦታ ፎካል ስመደብን የሰው ሃይል ችግር የለብንም፡፡

የማህበረሰቡ አመለካከት ችግር አለ፡፡ በየሩብ አመቱ እንኩዋን ግንዛቤ ለመፍጠር ሰራተኛውን ስንሰበስብ ከ105 ሰራተኛ 20 ወይም 30 ሰው ነው ሊገኝ የሚቸል፡፡ ይህ ቫይረስ አልጠፋም እንዴ፣ አሁንም አለ እንዴ እና የመሳሰሉትን ቀልድ መሰል ነገሮችን ይናገሩሃል፡፡ እና በጣም የአመለካከት ችግር አለ፡፡ ኮንዶም በየሽንት ቤቱ ስናስቀምጥ የረሳነውን ልታሳስቡን ነው የጠፋ በሽታ የሚል ሰው አለ፡፡

1. ወደ ፊት ቢሰሩ የምትለው ነገር ካለ

ፎካል ፐርሰን ተብሎ የሚመደበው አካል በጥሩ ሁኔታ ስልጠና የወሰደ ከፍ ያለ አረዳድ ያለው ሰው ቢሆን ጥሩ ነው

ጤና ቢሮ ተከታታይ ግምገማ ማድረግ አለበት፡፡ ይህ አንዱ ችግር ስለሆነ ነው፡፡

1. በመጨረሻ የምትለው ካለህ

በ2030 ይጠፋል ተብሎ ስትራቴጂ ተያዘ እንጂ አሁን ባለው ሁኔታ እስከመቼም አይጠፋ፡፡

**#7**

1. Please tell me about your responsibility and the work you do

We work as a mainstreaming. Regarding the issue of equity, we will bring it down to the zone and district level. In our sector, we do a quarterly based awareness creation campaign. We also help orphans. The worker takes a deduction from his salary and raises these children. When we say equitable access, for example, our office's wealthy clients come from far away to execute administrative issues at region, and at this time they can have sexual contact with another person.

So, they need condoms. But there is no condom supply. It could be fortunately if we distribute condoms at our sector so that people will be alert because it is an indirect way of sending a message that the disease is spreading. The disease is very neglected.

1. Please tell me if there is any part of the community that you are working on with special attention

One of the areas we focus on is the flower industry. And because it is an industry where women are employed in large numbers, we will work on it with focus.

1. If there is anything you can mention as a challenge to make it accessible, please.

For example, we have 106 employees in our office, and we have not been able to help all those who have the virus in their blood. We help those with low wages. So, we can't reach everyone because of lack of funding. We are doing this by giving 50 cents out of 100 Birr with the good will of the employee. The reason why we cannot work fairly in the 15 zones, for example, women were raped in the area where the war took place. Condom distribution, awareness work was needed but not done. Other challenges include lack of attention, financial constraints, and inability to work together. Our relationship with the health office is loose. There is also the problem of transportation. It is difficult for us to reach the desired area by arranging transportation. We entirely focused on reporting. We don't have any manpower problems when we assign focal person from all over the place. There is a problem with society's attitude. Every quarter, when we gather the employees to create awareness, 20 or 30 people out of 105 employees will be present. They will tell you funny things like this virus is not gone, is it still there and so on. And there is quite a problem of attitude. When we put condoms in every toilet, there is a person who says that are you going to push us to HIV despite we believe it is disappeared (የረሳነውን ልታሳስቡን ነው).

1. If there is anything you would like to do in the future

It is good if the focal person is a well-trained person with a high level of understanding. The Health Bureau should conduct a follow-up review. Because this is one of the problems.

1. Finally, if you have something to say

There is a strategy that it will disappear by 2030, but it will never disappear in its current state.

**#8**

1. እባክሽ ስለኤች አይ ቪ ኤዲስ ያለብሽን ኃላፊነትና ዝርዝር ስራዎች ንገሪኝ

ኃላፊነቴ ስራዓተ ጾታና ኤች አይ ቪ ኤዲስ ጉዳይ ባለሙያ ሁኘ ነው የምሰራው፡፡ በሴክተራችን በየወሩ የግንዛቤ ማስጨበጫ ውይይት ማድረግ ነው፣ ከዚያ ባሻገር በኮንደም ስርጭት ዙሪያ እንሰራለን፡፡ እስከ ታች ወረዳ ድረስ መዋቅር አለን፡፡ ኮሌጆችም አሉን፤ ነገር ግን ኮሎጆች ላይ ባለሙያ የለም፡፡ ነገር ግን በሚፈለገው ደረጃ ሁሉንም እኩል ተደራሽ እንሆናለን ማለት አይደለም፡፡

1. ተደራሽ እንዳትሆኑ ምክንያቶቾ ምንድን ናቸው

መደቡ አለመኖርና በቂ የሰው ኃይል አለመመደብ አንዱ ነው፡፡ ምክንያቱም ባለሙያ ካለ በተጠያቂነት እና ኃላፊነት ስሜት ስራዎችን በእቅድ ይከውናል፡፡ ውጤት ተኮር ይሞላለታል፤ ተጠያቂ ይሆናል፡፡ ፎካል ፐርሰን ስታደርግ ግን ከስራ ተደራቢነት ነው የሚሰራ ስለዚህ ከሰራው እሰየው ነው ካልሰራው ደግሞ ተጠያቂነት የለበትም፡፡ ስለዚህ ትክክለኛውን ሰው በትክክለኛው ቦታ አለመመደብ ነው፡፡ ኤች አይ ቪ ኤዲስ አገልግሎትን ጉዳይ ትኩረት ሰጥቶ የማየቱ ጉዳይ አናሳ ነው፡፡ ከማህበረሰቡም ሆነ ከመንግስትም ጉዳየ ሎ ወስዶ የመስራት ሁኔታ ላይ በጣም ትኩረት የለውም፡፡ ትኩረት ተሰጠው ማለት በጀት ይመደባል፣ የተመደበው በጀት የሰራውን ስራ ይገመገማል፤ እና ተጠያቂነትን ያመጣል፡፡ በእርግጥ በመመሪያ ደረጃ መስሪያ ቤቶች 2 ፐርሰነት ይመድባሉ፤ ይህንን ተግባራዊ የሚያደርጉ ግን ጥቂት መስሪያ ቤቶች ናቸው፡፡ ያን 2 ፐርሰነት ቢጠቀመው ባይጠቀመው ተጠያቂነት የለም፡፡ ምክንያቱም መጀመሪያውንም ያ ሁለት ፐርሰነት አመዳደብ ስሌት የሌለው በዘፈቀደ እንዲሁ 2 ፐርሰንት ተባለ እንጂ ግልጽ የሆነ ለምን ስራ ምን ያክል በጀት የሚል በእቅድ የተነደፈ የገንዘብ ፈሰስ አይደረግም፡፡ ስልጠና አንዴ ወስደህ አሰልጥነህ ስትመለስ በአንድ ጊዜ ያልቃል፡፡ ከዚሁ ከመስሪያ ቤት ላይ እንወያይ ስትል ደግሞ ሰራተኛው አይገኝም፡፡ መረጃ በማግኘት በኩል እና ስልጠና አሰጣጥ ስልቱ ደግሞ ያው እንደ ድሮው ነው፡፡ አንዳንድ ጊዜ አሰልጣኞች ሲቸገሩ አያለሁ፡፡ ማህበረሰቡም ሆነ ከመንግስት ትኩረት ማነስ ምክንያት በፊት ኤች አይ ቪ በክልል ደረጃ የራሱ ሴክተር ነበረው፤ እንደገና ሃብኮ ነበር፡፡ አሁን ግን ይህ ሁሉ ተሰረዘ እና በየሴክተሩ አንዳንድ ሰው ተደራቢ አድርጎ እንዲሰራው ተባለ እና ብዙም ትኩረት አልሰጠው አለ፡፡ እና አሁን ግን ለምሳሌ እኔ ጀንደር እና ኤች አይ ቪን ደርቤ ነው የምሰራ፡፡ ጀንደር ብቻውን ብዙ ስራ ነው፡፡ አንድ ባለሙያ የት ድረስ ወርዳ ስራ ትሰራለች፣ ምን ያክል ለውጥ ታመጣለች እሱም በራሱ አንዱ ተግዳሮት ነው፡፡

1. ዘርፈ ብዙ ተጎጂ የማህረሰብ ክፍልን ተጠቃሚ ከማድረግ አንጻር ያለው ምን ይመስላል

ይህ ነገር የለም ብየ ነው የማምነው ምክንያቱም አንደኛው ነገር የሰው ኃይል ይህን ሁሉ ያማከለ ስራ እንዲሰራ አለመመደብ እና የበጀት አመዳደብና አፈጻጸም ችግር አለ፡፡ ከታች ያሉ አገልግሎት አያገኙም የሚባሉትን ለመደገፍ በጀት ያስፈልጋል፡፡ ነገር ግን ሜኒስትሪሚንግ ማለት ሴክተሩ አካቶ ይሰራል ማለት ነው፡፡ ነገር ግን ግራውንድ ላይ ስታየው ምን ታቅዶ ምን ተሰራ የሚል አሰራር ስልት አታይም፡፡ ውጤታማ የሚሆነው ደግሞ ድጋፍና ክትትል ስታደርግ ነው፡፡ እንደኛ ሴክተር 2 ልጆች የምናግዛቸው አሉ፡፡ ከዚያ ባሻገር ግን ለይተን እንደ ተቋም አንሰራም፡፡ እንደዚህ ልዩ ትኩረት የሚሻቸውን በቼክሊስት ለማካተት አስተያየት በምንሰጥበት ጊዜ 2 ፐርሰነት ተመድቡዋል ወይስ አልተመደበም ነው ብዙ ጊዜ የሚፈለገው፡፡ እንደ አጠቃላይ ግን አሁን የሁላችንም ትኩረት ወደ ወቅታዊ ጉዳይ ነው፤ ጦርነቱ ሁሉንም ነገር ትኩረት መሳብና ጦርነት አካባቢ ያሉት ማህበረሰብ ክፍልንም መድረስ አይቻልም፡፡ አንዳንድ ጊዜ ደግሞ ማህረሰቡ በኩልም አገልግሎቱን ብታቀርብላቸውም ፍላጎት አለሞኖርና ጀሮ አለመስጠት ይስተዋላል፡፡ አሁን አሁን ደም ግፊት፣ ስኳርና ካንሰር ይፈራል ከኤች አይ ቪ ይልቅ፡፡ ይህን ቫይረስ አቅልሎ የማየት ነገር ይስተዋላል፡፡ ህመሙ ምንም ማለት እዳልሆነ የማየት ዝንባሌ አለ፡፡

1. ወደ ፊት ቢስተካከል ቀረኝ የየምትይው ነገር ካለ እባክሽ

ኮሌጅ፣ ትም/ት ቤቶችና ዩኒቨርሲቲዎች ላይ በንቃት መስራ አለብን ብየ አስባለሁ፡፡ ሌሎችን ግን ከላይ ነግሬሃለው፡፡

ትልቁ ነገር በፖሊሲም ሆነ በስትቴጂ ነድፎ መስራት የሚቻለው መዋቅሩን ማስተካከል ነው፡፡ ባለቤት ከሌለው ስራው ተሰራ ለማለት አይቻልም፡፡ የጤና ቢሮ ክትትልና ድጋፍ ያደርጉ ነበር አሁን ግን ክፍተት አለ፡፡ የመዋቅር ጉዳይ፣ ክትትል ጉዳይ ይስተካከል፡፡ ባይገርምህ መመሪያ ለማዘጋጀት እየሞከርሁ ነበር እናም ምን አሉኝ ከእኔ በላይ ያሉት ኃላፊዎች አሁን በዚህ ሰዓት ይህ ችግር አስፈላጊ ነው ወይ፣ ድከሙ ያላችሁ ማነው ነው ያሉን፡፡

1. ቀረ የምትይው ነገር ካለሽ

ምንም ቀረ የምለው ነገር የለኝም፡፡

**#8**

Amhara Regional State Work and Training Enterprise Office 25:02
1. Please tell me your responsibilities and tasks about HIV/AIDS

My responsibility is to work as a gender and HIV AIDS specialist. In our sector, we hold awareness creation every month, apart from that we work around condom distribution. We have a structure all the way down to the lower administration (Wereda)circuit. We also have collages. But there is no HIV personnel in colleges. But that doesn't mean we'll be equally accessible to everyone at the desired level.

2. What are your reasons for not being accessible to all?

One of them is the absence of the position (መደቡ አለመኖር) and not enough human resources. Because if there is assigned personal for HIV office, he will accomplish the work with a sense of accountability and responsibility. He will be evaluated for his performance. He will be responsible. But when you make a focal person who works for HIV/AIDS as a secondary job. It is good if I will do it, and if I don't work, there is no accountability. So, there is not putting the right person in the right place. Attention to HIV/AIDS services is rare. It is both from the community and the government that HIV/AIDS do not get attention, none take HIV/AIDS as own concern. If it gets attention, a budget can be allocated, then the allocated budget is evaluated for the work done; And it brings accountability. In fact, they allocate 2 percent, but few offices implement this. There is no accountability whether we use that 2 percent or not. Because in the first place, that two percent allocation, which has no calculation, was called 2 percent arbitrarily, not a clear way of budget allocation for how much work to be done, and not a planned financial investment. Once we give training for the staff, this 2 percent budget will end. The employees are not available when you invite them to discuss on HIV/AIDS if we make training in our workplace. Another the way of getting information and training is the same as before. Sometimes I see trainers struggling. Before the lack of attention of the community and the government, HIV had its own sector at the regional level; It was HAPCO. But now all this has been canceled and someone in every sector has been told to do it as an overlay and not paid much attention to it. And now, for example, I'm working on gender and HIV. Gender alone is a lot of work. How far a single person will go and work up to lower level, how much difference she will brings is a challenge in itself.

3. What does it look like in terms of benefiting a multi-faceted victim group?

I believe that this is not the case because one of the things is that human resources are not assigned to do all this intensive work and there is a problem with budget allocation and implementation. A budget is needed to support the underserved. But mainstreaming means that the sector should be inclusive. But when you see it on the ground, you don't see a strategy a monitoring and supervision of what has been planned and what has been done. It is effective when you provide support and supervision. As to our sector, there are 2 children that we help and grow. Beyond that, however, we do not operate by identifying the disadvantaged as an institution. A special attention in the checklist is given to whether 2 percent is allocated or not is often required when we give feedback. Generally, now, all of our attention is on a current issue. The war attracts everyone's attention and even the section of the society in the war zone cannot be reached. Sometimes, even if you offer the service to them, there is no interest, and it is noticed that they don't give ear to it. Now blood pressure, diabetes and cancer are more feared than HIV. There has been observed that people understatement this virus. There is a tendency to see HIV as nothing.

4. If there is anything you would like to be done in the future, please

I think we should actively work on colleges, schools and universities. But others I have told you above.

The biggest thing that can be done in terms of policy and strategy is to adjust the structure. Without an owner, the work cannot be said to be done. They used to monitor and support the health office, but now there is a gap. Fix structure issue, tracking issue. If you don't mind, I was trying to prepare a guideline and what did the officials above me say, is this problem important at this time, who are you tired on it?

5. If you have something left to be added, please.

I have nothing left to say.

**#9**

1. እባክሽ ስለ ኤች አይ ቪ ጉዳይ ያለብሽን ሃላፊነት

በአማራ ልማት ማህበር የፋሚሊ ፎከስድ ኤች አይ ቪ ፕሪቨንሽን ኬር ኤንድ ትሪትመንት ዩኤስአይዲ ፈንድድ ፕሮጀክት ውስጥ ነው የምሰራው፡፡ ከዚህ በፊት በነበረኝ ልምድም ሲዲሲ በሚደግፈው ፕሮጀክት ኤች አይ ቪ ሜኒስትሪሚንግ ላይም ሰርቻለሁ፡፡ ከሶስት አመት በላይ ሰርቻለሁ፡፡ ሶስት ዋና ስራዎች አሉት፡፡ እየመረመርን ቫይረሱ በደማቸው ያለባቸውን መድሃኒት እንዲጀምሩ ማድረግ፣ ኬር እና ድጋፍ የሚባል አሰራር ልጻናትም ለሁሉም ማበረሰብ ክፍል እና ሶስተኛው የመጀመረያ መከላከል ስራዎች ላይ እሰራለን፡፡ ከመንግስት ተቁዋም ጋር ተቀናጅተን እንሰራለን፡፡ቮለንተርስ አሉን፡፡ ከቀበሌ አደረጃጀቶች ጋር እንሰራለን፡፡

1. ያልሰራናቸው ስዎች አሉ ብለሽ የምታስያቸው አሉ

ኤች አይ ቪ መከላልን አንጻር በሚገባው ልክ እየሰራን ነው ብየ አላምንም፡፡ ምክንያቱም ከማህበረሰቡ ቸልተኛነት አንጻር በሚገባው ልክ እየሰራን አይደለም፡፡ ስለዚህ ተደራሽ ያልሆኑ ማህረሰብ ክፍል አሉን ማለት ነው፡፡

1. የኤች አይ ቪ ኤዲስ አገልግሎት ፍትሓዊ ተደራሽንት የምትነግሪኝ ነገር ካለሽ

ስራዎችን ለሁሉም ለመድረስ ካልቸራል ነገሮች አሉ፤ በግልጽ የማናወራቸው ነገሮች አሉ፡፡ በተለይ ስክሹዋል ኤክስፕሪያንስን በግልጽ ካለማውራታቸው የተነሳ መድረስ ያለብንን ሰዎች እንዳንደርስ ያደረገናል፡፡ ከኖርሙ ጋር ተያይዞ ብዙ ሰዎችን መድረስ አንችልም፡፡

ተጋላጭ የሚባሉ የማህበረሰብ ክፍሎችን ለይተን እንሰራለን፤ለምሳሌ ሴተኛ አዳሪዎች ላይ

1. ፍትሓዊ አገልግሎት ለመስጠት ተደራሽ ለማድረግ የሚያጋጥሙ ተግዳሮት

እስከ አሁን መረጃ ያልደረሳቸው የማህበረሰብ ክፍሎች አሉ፡፡ ፋይናስ ችግር እንደ ግለሰብም ድሃ የሆነ ማህበረሰብ የሚፈልገውን ለመግዛት አይችልም፡፡

አቴንሽን አለመኖር፡፡

ማህበረሰቡ በኩል ያለው ድክመት አንዱ ነው፡፡ ወንዶች ወደ አገልግሎት አለመምጣት፣ የህክምና አገልግሎት ለማግኘት ፍላጎታቸው ያነሰ ነው፣ ማስኩሊኒቲ ይመስለኛል፡፡ ሴቶች የባሎቻው ፍቃድ ከአገኙ በሁዋላ ነው ወደ አግልግሎቱ የሚመጡ፡፡ ባል ካፈቀደ አይመጡልህም፡፡ ሴቶች ወደ ተቁዋም ይመጣሉ፣ በእርግዝና ጊዜ፣ ልጆቻውን ለህክምና ለክትባት ይዘው ይመጣሉ ከወንዶች በተሻለ፡፡

1. ወደ ፊት ፍትሓዊ አገልግሎት ለማምጣት ቢሰሩ የምትያቸው

ኖርሙ ላይ ቢሰሩ፣ ተከታታይ ጥናት በማጥነት ችግሩ ላይ ያከሩ ስራዎች ቢሰሩ፣ መንግስት ትኩረት ሰጥቶት እደ በፊቱ መነጋገሪያ ቢያደርገው

1. ተጨማሪ ሐሳብ ካለሽ

የለኝም

**#9**

1. Please tell me about your responsibility regarding HIV

I work in Amhara Development Association's Family Focused HIV Prevention Care and Treatment USAID funded project. In my previous experience, I also worked on a CDC-supported project called HIV management. I have worked for more than three years.

It has three main functions. While we are performing HIV testing, we enforce them to start taking the medicine, and we have support system for all the children, and the prevention activities. We work together with the government. We have volunteers. We work with local organizations.

1. Please tell me if there is something that you have not done.

I do not believe that we are doing as well as we should in terms of preventing HIV. Because we are not doing as well as we should in terms of the negligence of the society. So, we have an unreachable social class.

1. If you have anything to tell me about equitable provision of HIV AIDS services, please.

There are cultural factors that make services accessible to all; There are things we don't talk about openly. Especially because they don't talk openly about sexual experiences, it keeps us from reaching the people we need to reach. We cannot reach more people with the norm.

We work on identifying vulnerable sections of the society, for example on prostitutes.

1. Please tell me if challenges to provide fair service

There are sections of the community that have not yet received information. Financial problem, as an individual, a poor society cannot afford to buy what it needs. Lack of attention. It is one of the weaknesses on the part of the community. Men not coming to services, less interested in seeking medical care, I think masculinity. Women come to the service only after obtaining the permission of the husband. If the husband do no allows, they will not come to you. Women come to health institution, during pregnancy, they bring their babies for treatment and vaccination better than men.

1. What you suggest to provide equitable service in the future

If they work on the norm, if they do serious work on the problem with a series of studies, if the government pays attention to it and makes it a topic of discussion.

1. If you have more ideas, please

I don't have.

**#10**

1. እባክዎ ስለ ኤች አይ ቪ ጉዳይ ያለብዎን ሓላፊነት ቢነግሩኝ

በኤች አይ ቪ ጉዳይ ለአምስት ዓመት ሰርቻለሁ፡፡ እቅድ ማውጣት፣ ክትትልና ግምገማ መድረግ

1. እባክዎ የምትሰጡዋቸውን አገልግሎት ለማሳለጥ የምትጠቀሙዋቸውን ስልቶች/ዘዴዎች ይገሩኝ

ኤች አይ ቪ ኤዲስ ጉዳዮችን የምሰራቸው ስራዎች ማካተትን በተመለከተ በሁለት መልኩ እንከፍላለን፡፡

አንደኛው ወሳኝ ማካተት ብለን የምንቆጥረው እኔ እንደ አንድ ባለሙያ ለትም/ት ቢሮ የሚሰጥ አጠቃላይ አገልግሎት፣ እና ሰራተኛው ተንቀሳቃሽ ስለሆነ ኮንዶም ማሰራጨት እና ቫይረሱ በደሙ ውስጥ ላለባቸው እርዳታ እንዲደረግ ማስተባበር፡፡

2ኛው ውጫዊ ሜንስትሪሚንግ የምንለው ነው፡፡ ከመምሪያዎች፣ ከት/ት ቤቶች ጋር መስራት ነው፡፡

3ኛ. ያልተሰሩ ስራዎች የምትላቸው አሉ

ከሰራናቸው ይልም ከመርህ ድረጃ እና ከጽንሰ ሐሳብ ደረጃ ከተሰሩት ይልቅ ያልተሰሩት ይልቃሉ፡፡ ለምሳሌ በደማቸው ያለ ልጆች ከአድሎና መገለል ነጻ ሁነው እንደማንኛውም ተማሪ በእኩል እንዲማሩ የሚያስችል የተለየ ስልታዊ አሰራር የለም፡፡ መገለል ደርሶባቸዋል ዋይ፣ ከደረሰባቸውስ ምን ይፈልጋሉ፣ ምን መደረግ አለበት እና ትም/ት ቤቶችና የጤናው ሴክተር ሊንክ መፈጠር ነበረበት፡፡ ምንም እንኩዋን በየቀበሌው ጤና ተቁዋማት/ክሊኒክ/ እና ጤና ኤክስቴንሽን ቢኖሩም የቅብብሎሽ አሰራር አለ ብለን አናምንም፡፡ ከክልል እስከ ወረዳም ሰንሰለቱን ተከትሎ በትክክል ታቅዶ የሚሰራ ስራ የለም፡፡ ማካተት መርህ ሁኖ ተግባር የለም፡፡ አካታችነት በትክክል እየተተገበረ ነው ወይ ብሎ ክትትል ማድረግ ላይም ችግር አለ፡፡

4ኛ. ከችግር አንጻር ዘርፈ ብዙ ናቸው

አንደኛ ከአደረጃጀት አንጻር ስንመለከት እኔ እንደ ትም/ት ቢሮ ሁለት የስራ ዘርፎችን (ስርዓተ ጾታ እና ኤች አይ ቪ ጉዳዮችን) ለመከታተል ስርዓቱ ተዘርግቱዋል፡፡ ስርዓቱ በቢሮ ደረጃ ነው ያለ፡፡ ይህ ስርዓት ትም/ት መምሪያ ላይ የለም፣ ወረዳ ላይ የለም፣ ትም/ት ቤቶች ላይ የለም፡፡ ስለዚህ ቅብብሎሽ የለም፣ ባለቤት የሌላቸው ስራዎች ተብለው ከሚነገሩ ስራዎች ውስጥ ይህ አንዱ ነው፡፡
ሁለተኛው ከአደረጃጀት ባለፈ የዚችን አገር እጣ ፈንታ እየመሩ ያሉት የፖለቲካ አመራሮች ናቸው፡፡ ምናልባት እንደቢሮ በመሪ እቅድ ላይ ተካቶ ይወርዳል፡፡ ማንም ቢጠየቅ እተሰራ ነው ሊልህ ይችላል በየሩብ አመት ሪፖርት ስለሚመጣ፡፡ ይሁን እንጂ የዚህ ውጤታማነት የህጻናትን መብት ከማስከበር፣ ትም/ት ቤቶች ለኤች አይ ቪ አጋላጭ ነጻ ስለመሆናቸው፣ ኤች አይ ቪ ጉዳይን አካተው እየሰሩ ስለመሆናቸው፣ የተሰራው ስራስ ምን ያክል ውጤታማ ነው እና መሰል ስራዎችን አካቶ ከመስራት አንጻር ክፍተት አለ፡፡ ስትራቴጂክ ጥናት ተደርጎ መሰል ችግች እንዲፈቱ የፖለቲካ አመራሩ ወሳኝነት አለው፡፡

በሌላ መልኩ ከፌደራል ጀምሮ እስከ ወረዳ የሚደርስ በበጀት የሚደግፍ አሰራር ያለ አይመስለኝም፡፡ በእርግጥ በስትራጂክ ሴክተሮች በጀት 2 ፐርሰንት ለኤች አይ ቪ ይመደብ ይባላል፡፡ ይመደብ ቢባል 1 ሚሊዮን ወይም 2 ሚሊዮን ብር ቢመደብ ነው፡፡ ይህ ብር ከአስር ሺህ በላይ ትም/ት ቤቶችን ለመድረስ ይቅርና ከየትም/ት ቤቱ 1 ፎካል ፐርሰን አስልጥኖ ስራው በቁዋሚነት እንዲሰራ ለማድረግ ይቸግራል፡፡ ባለፉት ዘመናት ብዙ አጋር ድርጅቶች ስለነበሩ ገንዘብ ድጋፍ ነበር፡፡ አሁን ግን ለውጥ አምጠተናል ብለን እነሱ በወጡበት ጊዜ አሁን ላይ ችግር ሲመጣ ግን የሚደርስ ነገር የለም፡፡ የበጀት ችግር ማነቆ ነው፡፡

አካባቢያዊ ሀብትንም በአግባቡ ከመጠቀም አንጻር ትልቅ ችግር አለ፡፡

እድሜያቸውን መሰረት ያደረገ፣ ወቅቱን ያገናዘበ የማሰልጠና ቱሎች ያስፈልጋሉ፡፡ ምናልባት አሁን እየተጠቀምንባቸው ያሉ ማንዋሎች ከአስር አመት በፊት የተዘጋጁ የአቻ ለአቻ እና የህይወት ክህሎት ማሰልጠኛ ዶክመንቶች ናቸው፡፡ አሁን ላሉት ህጻናት ከዘመኑ ጋር ሲነጻጸር የሚመጥኑ አይደሉም፡፡ እነዚህን አስተካክሎ የሚያወርድ አካል ያስፈልጋል፡፡ ስለዚህ ማሰልጠኛ ማንዋሎች ላይ ችግር አለ፡፡

ሌላው የመረጃ ስርዓቱ ወጥ የሆነ በሃብኮ የተዘጋጀ ቱል አለ፤ ነገር ግን አሁን ሃብኮ የለም፡፡ እሱን እየተጠቀምን ቢሆንም በእሱ የሚሰበሰበው መረጃ ተዓማኒነቱ ከዚህ ግባ የሚባል አይደለም፡፡ ሴክተሩ በሌለበት እና በፈረሰበት ሁኔታ እያለ ግን እሱ ባዘጋጀው ቱል ነው እየሰበሰብን ያለነው፡፡ ተዓማኒ አይደለም፡፡ መረጃን በመሰብሰብ እንደ ክልልም እንደ ፌደራል እንልካለን፡፡ የመጣው መረጃ ትክክል ነው ወይ ብሎ የሚገመግም አካል የለም፡፡ ስለዚህ መረጃው ተዓማኒነት ላይ ጥያቄ አለ፡፡

ኤች አይ ቪ ኤዲስን በተመለከተ በስትራቴጂክ ሴክተሮች እየመሩት ያሉት በሙያው ዘርፍ የሉ ቢሆኑ ወይም ደግሞ አጭር ጊዜም ሆነ ረጅም ጊዜ ስልጠናውን የወሰዱ ቢሆኑ ስራውን ለመስራት ያመቻል፡፡ ለምሳሌ እኔ አንዳንድ ጊዜ ማይለከተኝን እየሰራሁ እንደሆነ ይሰማኛል፡፡ ትምህርት ቤቶች ላይም የሚመደቡ ፎካል ፐርሰኖች ለበሽታው ቅርብ የሆኑ ሙያተኞች ቢሆኑ ጥሩ ነው፡፡ ባለፉት 5 አመታት ለሙያተኛ የሰጠነው ስልጠና የለም፡፡

አሰራሩ ስትራቴጂክ አይደለም፤ የወል ነው፡፡ ከጤናው ዘርፍ ጋር ቅንጅት የለም፡፡

የማህበረሰቡ ባህል ወይም እሴት ፍትሓዊነት ላይ ጫና አለው ትላለህ… እኔም አንዱ የማህረሰብ አካል ስለሆንሁ ቫይረሱ በደማቸው ላይ ከአለባቸው ሰዎች ጋር ግንኙነት እንዲኖረን ይከብዳል፡፡ እሴቱ ተረዳድቶና ተደጋግፎ እንድንኖር እሴታችን አግዞናል፡፡

ሌላው ህግ ይወጣል ግን አተገባበር ላይ ችግር አለ፡፡

በኢኮኖሚ የደከሙ ሰዎችን ማገዝ ሁኔታ አለ ግን ስርዓት አልተዘጋጀላቸውም፤ በግለሰቦች ፍላጎት ላይ የተንጠለጠሉ ናቸው፡፡

ሁለት ጊዜ እና ሶስት ጊዜ ተጎጂ የሆኑ ማህበረሰብ ክፍሎችን ለይቶ የመስራት አሰራር ካለ ብትነግረኝ፡ ቅድም እንዳልሁህ ሶስት ጊዜ እና አራት ጊዜ ተጎጂ የሆኑ የማህረሰብ ክፍልን ለይቶ መስራት አሰራር የለም፡፡ የወል አሰራር ነው ያለ፡፡

5. ወደ ፊት ቢስተካከሉና ቢሰሩ የምትላቸው ካሉህ

ሴክተር አካውንቴብል የሚያደርግ አሰራር መዘርጋት ቢኖር፡፡ አካውንቴቢሊቲ ችግር አለ፡፡ አካታች ስርዓት መፍጠርና መተግበር፡፡ ካልሆነ በአንድ ቃል አከቶ መስራት የሚለውን ስለተጠቀሰ ሳይሆን ተጠያቂነት በሆነ መልኩ መሰራት አለበት፡፡ ስትራቲጂክ ዶክመት ከኢንተርኔት ፈልገን እንጂ በቀላሉ ይሄ ነው የምንለው የለንም፡፡

የምንሰራው ስራ ዲስክሪሚናሪ ነው ማለት ይቻላል፡፡ ለምሳሌ ማየት የተሳነውን ማየት ከሚችሉ ጋር አብሮ አዳብሎ ስልጠና መስተጠት የስልጠና አሰጣጡን ማየት ለሚችሉት ብቻ አድርገህ ለሁሉም እኩል ስልጠና ሰጠሁ ብትል አስደናቂ ነው፡፡ እኔ የሆነ ስልጠና ላይ ስሳተፍ የሚሰጠውን ስልጠና በድምጽ ቅረጥልና ስጠኝ ነበር ያለኝ፤ የምንሰራው ስራ አድሎአዊ እንደነበር አሁን ነው የገባኝ፡፡ ማየት እንደሚችሉት ድርሻው እንዳይቀርበት ብቻ ደፍተር፣ እስክብሪቶ እና ማንዋሉን እንሰጠዋለን እንጂ የእውነት ይጠቀምበታል ወይ ብለን ብንጠይቅ ግን የለም፤ አይሆንም፡፡

**#10**

1. Please tell me about your responsibility regarding HIV

I have worked on HIV for five years. Planning, monitoring and evaluation.

1. Please tell me the strategies/methods you use to improve the service you provide

When it comes to the inclusion of HIV/AIDS in my work, we divide it in two ways.

One of the important inclusions is the comprehensive service that I, as a professional, provide to the education office, and because the staff is mobile, distributing condoms and coordinating assistance for those with the virus in their blood.

The 2nd is what we call external mainstreaming. Working with education sectors and schools.

1. Please tell me if there are unfinished works.

From the level of principle and concept, the ones that are not done are much more than the ones that are done. For example, there is no specific system that allows children with HIV to learn equally like any other student, free from discrimination and isolation. They have been stigmatized, what do they want if it happens to them, what should be done and the link between schools and the health sector should be created. Although there are health centers/clinics/and health extensions in every Kebele, we do not believe that there is a relay system. There is no properly planned work following the chain from region to district. Inclusiveness is not in principle and in practice. There is also a problem with monitoring whether inclusiveness is being implemented properly.

They are multifaceted in terms of problems.

First, from an organizational point of view, the system has been developed to monitor two areas of work (gender and HIV issues) as an education office. The system is at office level only. This system does not exist in the education department, it does not exist in the district, it does not exist in the schools. So, this is one of those jobs that are called no relay, ownerless jobs.

The second is the political leaders who are leading the destiny of this country beyond organization. It will probably be included in the master plan as an office. If anyone asks, they can tell you that it is being done, because the report comes every quarter. However, there is a gap in terms of the effectiveness of this in terms of protecting the rights of children, whether schools are free for HIV exposure, whether they are working with HIV issues, how effective the work is, and doing such work. It is important that the political leadership have significant impact to make a strategic study and solve such problems. Otherwise, I don't think there is a system that supports the budget from the federal level to the district. In fact, it is said that 2 percent of the budget of strategic sectors should be allocated to HIV. If it is to be allocated, it is if 1 million or 2 million birr is allocated. It will be difficult for this money to reach more than ten thousand schools, let alone train 1 focal person from each school to make the work effectively. In the past, there were many partner organizations so there was financial support. But now when they came out saying that we have bring a change, now when there is a problem, nothing happens. The budget problem is a bottleneck.

There is also a big problem in terms of proper use of local resources.

Age-based, seasonal training tools are needed. Perhaps the manuals we're using now are peer-to-peer and life skills training documents from a decade ago. They are not suitable for today's children compared to the current technology-supported period. It is needed to fix these and distribute them. So, there is a problem with training manuals. There is also a tool developed by HAPCO that is consistent with the information system. But now there is no HIV/AIDSPCO. Even if we use it, the reliability of the information it collects is under question. While the sector is non-existent and in a broken state, we are collecting with the tool he has prepared. It is not reliable. We collect information and send it to the state and federal level. There is no body that evaluates whether the information provided is correct. So, there is a question about the reliability of the information.

In the case of HIV/AIDS, those who are leading it in strategic sectors will be able to do the work if they are in the professional field or have received short or long-term training. For example, I sometimes feel like I'm doing things I don't care about. It is good if focal persons assigned to schools are professionals close to the disease. In the last 5 years, we have not given any training to a professional or focal person. The approach is not strategic; It is public. There is no coordination with the health sector.

1. Would you say that the society's culture or values ​​have pressure on equitable service provision?

I am also a part of the society, so it is difficult for us to have a relationship with people who have the virus in their blood. Our values ​​help us to live with each other.

Another law makes, but there is a problem in its implementation.

There is a situation to help people who are economically exhausted, but no system has been prepared for them. They depend on individual needs. If you could tell me if there is a system for identifying groups of society that have been victimized twice or thrice, as I told you earlier, there is no system for identifying groups that have been victimized three times or four times. It is a public system.

1. If you have anything to say to be corrected in the future, please.

If there is a system that makes the sector accountable. There is an accountability problem. Creating and implementing an inclusive system. If not, it should be done in a responsible way, not because it is mentioned by one word. We looked for a strategic document on the internet, but we simply do not have this.

The work we do is almost discriminatory. For example, training the blind with those who can see is amazing if you say that you gave the training only to those able to see and gave equal training to all. When I was participating in some training, a blind man asked me that I record my voice and give it to him. I just realized that the work I were doing was biased. As you can see, I give him a notebook, a pencil and a manual just so that he doesn't miss out on his share, but if we ask if he really uses it, he doesn't. no way.

**#11**

1. ስለ አንቺ ኃላፊነትና ስለምትሰሩት ስራ ንገሪኝ

የኤች አይ ቪ ኤዲስና ስርዓተ-ጾታ በተመለከተ ነው ያለብኝ ኃላፊነት፡፡ ከ1997 ዓ.ም ጀምሮ ነው ከዚህ ብሮ እየተተገበረ ያለው፡፡ እና ከዚያ ጊዜ ጀምሮ ነው ከዚህ ቢሮ እየሰራን ያለነው፡፡ ያው የምንሰራው ከክልሉ ጤና ጥበቃ ቢሮ ጋር ነው አብረን እየሰራን ያለነው፡፡ እነርሱ እርዳታ የሚያስፈልጋቸውን ወላጅ አልባ ልጆች አጣርተው የሚረዳቸው የለም ከተባለ እኛ እንረዳቸዋለን፡፡ እንደ አቅማችን ማለት ነው፡፡ ስንረዳ ግን ከሌሎች ለየት የሚደርገን ነገር አለ፡፡ ለምሳሌ አንተ ልጅ ወልደህ አሳድገኸው አንድ ነገር ቢሆን አብርኸው ትቀጥላለህ፤ ልክ እንደዚህ ሁሉ እነዚህ ልጆች ትምህርታቸውን አስተምረን ወጤት ካልመጣ ሌላ ስልጠና እናሰጣቸዋለን፡፡ በዚያ ስልጠና መሰረት የራሳቸው የሆነ ዘላቂ እድል ፈጥረንላቸው ነው እራሳቸውን የምናስችላቸው፡፡ እስከዛሬ ድረስ አምስት ልጆችን የስራ እድል ፈጥረንላቸዋል፡፡ እራሳቸውን ችለዋል፣ አግብተዋልል ወልደዋል፣ ስራ እየሰሩ ነው፡፡ አሁን ደግሞ አምስት ልጆችን ተቀብለን እያሳደግን ነው፡፡ ከቤታቸው ሁሉ ሂደን እንጠይቃቸዋለን፡፡ ሌላው በመንግስት ደረጃ ሁለት ፐርሰንት ተፈቅዱዋል፡፡ በዚህ ስብሰተባ ትሰበሰብበታለህ፣ ኮንዶም ትዛበታለህ፣ ብሮሸር ታሰራጭበታለህ የሚያስፈልገውን ነገር እናደርግበታለን፡፡ ሰራተኛውን በየወሩ እየሰበሰብን እናወያይ ነበር፡፡ አሁን ግን በተለያየ ምክንያት ተቁዋርጡዋል፡፡ አንደኛ ኮሮና በመጣ ጊዜ ተቁዋረጠ፡፡ ከዚያ በሁዋላ እንደገና የእቅድ አፈጻጸም በሚባልበት ጊዜ ከእቅድ አፈጻጸሙ ጋር አብረን ውይይት እናደርጋለን፡፡ ሰዓት ይሰጠናል ምክንያም ሁሉም ሰራተኛ ይኛል ማለት ነው፡፡ ያን ጊዜ በኤች አይ ቪ ኤዲስ ዙሪያ፣ በሴቶች ዙሪያ ስልጠና እንሰጣለን፡፡ እውነት የተዘጋጀ ስልጠና ነው የምንሰጥ፡፡ አንዳንድ ጊዜ ባለሙያ እንጋብዛለን፡፡ ሁልጊዜ ሲደጋገም አንድ ሰው እንትን እንዳይል ባለሙያ እንጋብዝና ስልጠናውን እናሰጣለን፡፡ ስራችንን እየሰራን ነው ያለነው፡፡ ይህ አሁን የምነግህ በጀት ህጻናት የሚረዱት ሁሉም ሰራተኛ ገንዘብ ያዋጣል፡፡ ሁሉም አባል ይሆናል፤ ስትነግረው እምቢ የሚል የለም፡፡ ልጆችን እያመጣሁ አሳያቸዋለሁ፤ እያደጉ ሲሄዱ ሁሉም ሰራተኛ ደስ ይለዋል፡፡ እንደየችግራቸው መጠን እስከ አንድ ሺህ ብር የምንረዳቸው አሉ፡፡ ለባዓል መዋያ ለገና ለ1 ህጻን 1000 ለ5ቱ 5000 በብር እና ለፋሲካ ደግሞ ለ1 ህጻን 1500 ለ5ቱ 7500 በብር አርዳታ አድርገናለል፡፡

1. ይህ አሰራር በስራችሁ ላለው መዋቅር እስከወረዳ ይደርሳል

እስከ ወረዳ ድረስ የለም፡፡ ሁሉንም ዞኖች ግን እንዲሰሩ እቅድ ልከናል፡፡ ሰርተዋል አልሰሩም ሪፖርት ይልኩልኛል፤ እከታተላለሁ፡፡ ለምሳሌ እኔ በዓመት 4 ጊዜ ውይይት አስደርጋለሁ፡፡ እናም በየዞኑ ያሉትን እጠይቃቸዋለሁ፤ ከዚህም በላይ እንዲሰሩ እነግራቸዋለሁ፡፡ ለደንበኞቻችን ደግሞ በራሪ ወረቀት አስቀምጣለሁ፡፡ በደንበኞች ዙሪያ ከዚያ የዘለለ የሰራሁት ስራ የለም፡፡

1. የኤች አይ ቪ ቫይረስ አገልግሎትን ለሁሉም ተደራሽ ከማድረግ አንጻር ያለሽ አተያይ እንዴት ነው

ለሁሉም ተደራሽ ነው ብየ አላስብም፡፡ ከእኔ ከራሴ ስጀምር በአሁኑ ሰዓት እየሰራን ነው ብየ አላስብም፡፡ ለምንድነው ብለህ ስትጠይቅ… ብዙ ምክንያቶች አሉ፡፡ አንደኛ የበጀት ጉዳይ ነው፤ ደስ ሲላቸው የሚፈቅዱት፣ ደስ ሳይላቸው ደግሞ የማይፈቅዱት ነው፤ መብትሽ አደለም ተብየ ሁሉ አውቃለሁ፡፡ ግን መንግስት ለስራ ማስኬጃ ሁለት ፐርሰንት ተብሎ ተቀምጡዋል፡፡ እኔ ሁልጊዜ በጀት አስይዛለሁ፤ በመጨረሻ ግን ይህ መብትሽ አይደለም የሚባልበት ሁኔታ አለ፡፡ ዞሮ ዞሮ ሆነም አልሆነም ይህ ለሰራተኛ ማወያያ እስከተፈቀደ ድረስ ግዴታ ነው ማወያየት አለብኝ፡፡ አዎ ግን እስካለ ድረስ ነው እንጂ ከሌለ ምንም ማድረግ የሚቻልበት ሁኔታ አደለም፡፡ ምንም ማድረግ አይቻልም አይልም ቅድሚያ የሚሰጠው ስራ ነው፡፡ ህይወት እኮ ነው እየጠፋ ያለ፡፡ አሁን እንዲያውም እየተዘናጋ ሰው በተለያየ ችግር ውስጥ ሁኖ እየተዘናጋ እያለቀ ነው፡፡ እያለቀ ነው እውነቴን ነው የምነግርህ እያለቀ ነው፡፡ ግን በቃ እረስተነዋል፡፡ ከላይ እስከ ታች እረስተነዋል፡፡ ይህ የሁላችንም ጥፋት ነው፡፡

1. ከፋይናንስ በተጨማሪ ፍትሓዊ ተደራሽነትን ለመስራት ሚያስቸግር ካለ ብትነግሪኝ

ሊደርሽፖችም አሉ፡፡ እኔም የግድ መስራት አለብኝ ብየ እሞግታለሁ፡፡ ለምሳሌ ሰሞኑን ስልጠና ሰጥቻለሁ፡፡ ብዙዎች ተዘናግተናል ነው የሚሉት፡፡ እኛ አሁን እየሰራን ያለን ሰራተኞቻችን ወደ ፊልድ ሲወጡ ስለበሽታው እንዳይረሱ ማስታወስና ደንበኞቻችን በበራሪ ወረቀት መረጃ እንዲደርሳቸው እናደርለን፡፡ በሴክተሩ በዋናነት ለስራው ብቻ ትኩረት የማድረግ ሁኔታ አለ፡፡ ለስራ ማስኬጃ ለቢሮ ያልፈቀዱልህ ለሌላው ይፈቅዳሉ ማለት አይቻልም፤ በአብዛኛው በእርግጥ የኔም ጥፋት ሊሆን ይችላል ብየ አስባለሁ፡፡ እኛ የምንገናኘው ከነጋዴዎች ጋር ነው፡፡

ሌላው ሁሉም ይመለከተኛል ብሎ ጀሮ አለመስጠት ነው፡፡ እኔን ጨምሮ አመራሮችም ሁሉም ለኤች አይ ቪ የሚገባውን ያክል ትኩረት እያደረግን አይደለም፡፡ ሁሉም ኃላፊነት ሰጥቶት እየተሰራ አይደለም፡፡ ትኩረት ማነስ አለ፡፡ ጤና ቢሮውም ከእኛ ጋር ጠንካራ ቁርኝት አለው ብየ አላምንም፡፡ የእቅድ አፈጻጸም ግምገማ ላይ ብቻ ሰርተናል ለማለት ነው እንጂ በሚገባው ልክ አይከታተሉም፡፡ ድሮ ሲከታተሉን በጣም ነበር የምንሰራ፤ እውነቴን ነው የምነግርህ፡፡ ከሁሉም በኩል ትኩረት ማነስ ችግር አለ፡፡ ድሮ ይዞት በነበረው አሰራር ጤና ቢሮም ሁላችንም ብንቀጥል ጥሩ ነበር፡፡

ብዙ ጊዜ የምንሰራው ስራ ግን የወል ነው፡፡ ተደራሽ ያልሆኑትንም አብረን እናገኛቸዋን ብየ አምናለሁ፡፡ ነገር ግን ለየት ያለ አሰራር የበለጠ ለበሽታው ተጠቂ የሆኑትን ወይም ለአገልግሎት ተደራሽ አይደሉም ብለን አስበን የተለየ አሰራር አንከተልም፡፡

የባህል ካልቸር ጉዳይ ደግሞ በሴቶች ጉዳይ ያለው ነገር ከዚያ ጋር ተያይዞ ያለው ነገር እነሱንም ያገኛቸዋል፡፡ በእርግጥ ይህ ነገር አሁን አሁን እየቀነሰ ነው፡፡

ሌላው ግን ኮቪድም ጦርነቱም ኤች አይ ቪ ጉዳይን ትኩረት አድርግን እንዳንሰራ ተግዳሮት ሁኖብን ነበር፣ አሁን ድረስ ችግር ነው፡፡ ምክንያቱም በጀቱ በሙሉ ወደ እነዚህ ጉዳይ ነው ፈሰስ የሚደረገው፡፡ ለስራ ማሰኬጃ የተባለው ማንኛውም ነገር ወደ ጦርነቱ ነው የዞረው፡፡

1. ተጨማሪ ተግደሮት የሆነብሽን የምትጠቅሽው ነገር አለ

አንደኛ የአገሪቱ አለመረጋጋት ነው፤ እራሳችን ኃላፊነት ወስደን እንዳንሰራ አድርጎናል፡፡ ሁለተኛ የሚመደበው ያ ሁለት ፐርሰንቱ በጀት ምን ላድርገው ስትል፣ለማወያያም ለምንም የታቀደውን ያክል እንድንሰራ አያደርግም፡፡ የኤች አይ ቪ ጉዳይ እንደ ተጨማሪ እንጂ እንደ ዋና ስራ አለመቆጠር አለ፡፡

1. ፍትሓዊ የኤች አይ ቪ አገልግሎት እንዲኖር ወደ ፊት ቢሰራ ወይም ቢስተካከል የምትይው ካለሽ

ሁላችንም ከላይ እስከ ታች ሁሉም ትኩረት አድርገን እንድንሰራ ነው፡፡ ሰው እያለቀ ነው፡፡ ምክንያቱም ሰው ሰው በጦርነት ብቻ አይደለም እየሞተ ያለው በዚህ በሽታም ሰው እየሞተ ነው፡፡ አንዳንዱ እኮ ሲናደድ፣ ደስ ሲለው እሩጦ ወደ ዝሙት የሚሄድ ሰው አለ፤ መጥፎ ድርጊት የሚፈጽም አለ፡፡ አንድ ወቅት ያጋጠመን ጉዳይ ለማስታወስ ያክል ስልጠና ስንሰጥ ውለን ሌሊት አልጋ ከያዝንበት አንደኛዋ ሴት ስትጮህ ምንድን ነው ብለን ከያለንበት ስንወጣ ስልጠናውን ሲወስድ የነበረ ሰው ነው…፡፡ እና ሰው ብትነግረውም አልሰማ ብሉዋል፡፡ አገራችን ሰላም ያድርጋት እንጂ ስራ የምንሰራ ነው የሚመስለኝ፤ የሚያስቆመን ነገር የለም፤ ይቀጥላል፤ ህይወት እስካለ ድረስ ይቀጥላል፡፡ ሀገራችንን ሰላም ያድርግልን፡፡ በዋነኝነት ስራው ይሰራ ካልን ግን በሙያው በደንብ የሰለጠነ ሰው ተመድቦ ከላይ እስከታች በተጠናከረ እና ቀጣይነት ባለው አሰራር መሰራት አለበት፡፡ ሪፖርት ለመስራት እንኩዋን ተሎ አያልቅልኝም፤ ስራ ልስራ ካልህ የሀያ ዞን ሪፖርት በትክክል ልገምግም ካልህ አትችልም፡፡ እኔ በሴቴችም በኤች አይቪም ሁለቱን መስራት በጣም ከባድ ነው፡፡ ስለዚህ የሰው ኃይል አመዳደብ ላይ ቢስተካከል፤ ወደ ስራው ልግባ ካልህ በትክክል ማስሄድ አይቻልም፡፡ የክልሉ የንግድ ዙሪያ ያለውን የማህረሰብ ክፍል በሴቶችና በኤች አይ ቪ ስራ የምሰራ እኔ ነኝ፡፡ አስበኸዋል በክልሉ ነው የምልህ…በዚህ ዙሪያ ያሉትን ሴት ነጋዴዎች ከላይ እስከታች ድረስ ውጤታማ ናቸው ወይ፣ ለምን ውጤታማ አልሆኑም፣ ለምን ፈቃዳቸውን ሰረዙ ብለህ ልስራ ካልህ ለዚህ ሁሉ ለአማራ ክልል እኔ ነኝ አስበኸዋል…፡፡ ሁሉንም ነው እንግዲህ አካል-ጉዳተኛም አለ፣ ኤች አይ ቪም አለ፡፡ እና በዚህ ሁኔታ ፍትሐዊ አሰራር ስለመዘርጋት ማሰብና መስራት ይቅርና በሐሳብ ደረጃ ስራዎችን ወረቀት ላይ ለማስፈር እንኩዋን ሁሉን መሸፈን አትችልም፡፡ እና መዋቅር ግን ያስፈልገዋል፡፡ ድሃ የሚባሉ ሰዎችን ማገዝ አስፈላጊ ነው ምክንያቱም ድሃ የሆኑ የማህረሰብ ክፍል ወደ አልባሌ ስራ ይሰማራሉ፡፡ ለምሳሌ እዚህ ጋር መናገር ባያስፈልግም ሰሞኑን እንድናሳድገው የተቀበልነው ልጅ ሁለተኛ ክፍል እያለ አሁን ሶስተኛ ክፍል ነው እስኪ ንገሪያት ለእናቴ እየሸረሞጠች እኮ ነው አለኝ ከእናቱ ፊት፤ በጣም ነው የደገነጥሁት እና ያፈርሁት፤ እሱዋም ተቆጣች፤ እና እኔም አይ ተይው ህጻን ስለሆነ ነው ግን ለምን እንዲህ ታደርጊያለሽ ስላት ዝም አለች፡፡ ይች ሴት ግን የገንዘብ ችግር ነው እንደዚህ አይነት ስራ ውስጥ እንድትገባ የሚያደርጋት እና እኛ የምንፈታው ይህንን ነው፡፡አሁን ይች ሴት በሽተኛ ሁናለች፡፡ ስለዚህ የገንዘብ ችግራቸውን ለመቅረፍ በገንዘብ እናግዛለን በምንችለው ልክ፡፡

1. ቀረ የምትይው ነገር ካለ

ቀረ የምለው ነገር የለኝም፡፡ ነገር ግን ሌላው ግን በዚህ መደብ ላይ እያለሁ እኔ እድገት አልተሰራልኝም፤ የአገልግሎት ዘመኔ እየጨመረ በሄደ ቁጥር አንደ ሌላው ሰራተኛ ማደግ አለብኝ ብየ አምናለሁ፤ የበለጠ እና የተሻለ ስራም እንድንሰራ ያደርጋል፡፡

**#11**

1. Tell me about your responsibilities and the work you do

My responsibility is about HIV AIDS and gender. It has been implemented since 1997. And since then, we have been working from this office. We are working together with the regional health office. They screen the orphans who need help and if there is no one to help them, we help them. It means according to our ability. But when we help them, there is something that sets us different from others. For example, if you have a child and raise him, if something happens, you will continue. Like all these children, we teach them and if they don't pass to university level, we give them another training. Based on that training, we have created a sustainable opportunity for them to economically empower themselves. Till date, we have created job opportunities for five children. They are independent, married, have children, and are working. Now we are raising five children. We will go to all their houses and ask them. Two percent is allowed at the state level to undertake meeting, distribute condoms, distribute brochures and we will do whatever is necessary. We used to meet the staff every month and discuss. But now it is interrupted for different reasons. It changed when the first Corona came. After that, we will have a discussion when they asked our plan implementation. Then we will provide training around HIV/AIDS, around women. We provide real training. Sometimes we invite an expert. We always invite an expert and give the training so that no one will say anything when it is repeated. We are doing our job. This budget I'm telling you right now will contribute money to every employee who helps children. Everyone will be a member. When you tell him, no one will say no. I will bring children and show them; Every employee is happy as they grow. Depending on the extent of their problems, we can help them up to one thousand birr. We helped children, like For Christmas, 1000 birr for 1 child, 5000 birr for 5 children, and 1500 birr for 1 child for 5 children, 7500 birr for Easter.

2. Is this procedure is applied to the structure of your work up to the district level?

we have sent a plan for all zones to work. They will send me a report whether it worked or not. I will follow up. For example, I have 4-times meeting per year. And I will ask those in each zone; I tell them to do more than that. I will also keep a flyer for our customers. I have never done more work around clients than that.

3. How do you think about making HIV services accessible to everyone?

I don't think it's accessible to everyone. When I started with myself, I don't think we are currently working. When you ask why... there are many reasons. First is the budget issue; when they like it, they allow it, and when they don't like it, they don't, I have been told that... you know it's not my right to ask budget. But the government has set it as two percent for operation.

I always plan a budget and inform to higher officials; But finally, there is a situation where it is said that this is not your right. After all, whether or not this is allowed for employee discussion, I must do my plan. Yes, but as long as it exists, nothing can be done without it. It does not say that nothing can be done because it is a priority problem. Many lose their life. Now, people are getting distracted and getting distracted in different problems. It's running out, I'm telling you the truth, it's running out. But we just forget about it. We forget it from top to bottom. This should be the fault of all of us.

4. In addition to finance, if there is any difficulty in making equitable service provision, please tell me.

There are also leaders. I would argue that I must work too. For example, I provide training recently. Many say we forget HIV. What we are working on now is to remind our employees not to forget about the disease when they go out to the field, and to send information to our customers through leaflets. In the sector, there is a situation of focusing mainly on the work. It is impossible to say that those who did not allow you to run a meeting in an office, will allow you to pay per diem to someone else and send him to zone or district level to provide training; I think it's probably mostly my fault too. Our customers are traders. Another thing is not to pretend that everyone ignores this virus. All leaders, including me, are not paying as much attention to HIV as it deserves. Not everyone is being held accountable. There is a lack of attention. I do not believe that the health office has a strong connection with us. It is to say that we have only worked on the evaluation of the annual performance of the plan, but they do not follow up properly. We used to work a lot when they followed us. I am telling you the truth. There is a lack of attention on all sides. It would be good if we all continue with the health office as it was in the past. But most of the work we do is public, not tailored. I believe that we can find the inaccessible ones together. However, we do not follow a specific procedure because we think that those who are more susceptible to the disease or are not accessible to services.

The issue of culture and the things that are related to the issue of women also affect them. Of course, this thing is going down now. The other thing is that we were challenged by the Covid and the war to focus on the HIV issue, which is still a problem. Because the entire budget is spent on these issues. Anything that was meant to be operational turned to war.

5. Have you something you can mention that is more challenging equitable HIV services delivery?

First is the instability of the country. It prevented us from taking responsibility ourselves. Second, that two percent of the budget that will be allocated will not make us do more than what was planned. The issue of HIV is seen as an add-on, not a main task.

6. Have you anything you would like to improve in the future to deliver equitable HIV service?

So that we all work together from top to bottom. Many people are infected and died due to this virus. Because people are dying not only in war, but also in this disease. There is a person who runs away and commits unprotected sex when he is angry. There is someone who does bad things. One time we had a case to remember, when we were doing training, when one of the women we were sleeping with screamed what was going on, it was a person who was taking the training. And even if you tell someone, they won't listen. I think we should work to make our country peaceful. There is nothing stopping us. We will continue work on it as long as life persist. May God bring peace to our country.

If we agree to perform the work effectively, a person who is well trained in the profession should be assigned and it should be done in a consolidated and continuous manner from top to bottom. I never run with time to report. If you're going to do a job, you're not going to be able to properly review the twenty Zone report. It is very difficult for me to do both with women and HIV issues. Therefore, if the allocation of human resources is adjusted; If you try to log in to the task, it cannot run properly. I am the one who works with women and HIV in the business sector of the region. I am telling you that you have thought about it only me address the region...if you want to work on the women traders around the region whether they are effective from top to bottom, why they are not effective, why they revoked their licenses, you have thought that I am for the Amhara region for all this...

It's all there, there's the disabled, there's HIV. And in this case, you can't even think about and work on establishing a fair system, ideally you can't cover everything to put jobs on paper. And it needs structure. It is important to help the so-called poor because poor people tend to work in rural areas. For example, although it doesn't need to be said here, the boy we recently accepted to raise was in the second grade and now he is in the third grade. I was so embarrassed and ashamed. And she got angry. And I said no, it's because he's a baby, but why are you doing this to her, she kept silent.

But this woman's financial problem is what makes her go into this kind of work and that is what we are going to solve. Now this woman is sick. So we will help financially to solve their financial problems as much as we can.

7. If there is anything left to see

I have nothing left to say. But the other thing is that while I was in this position, I was not promoted. I believe that as my term of service increases, I need to be promoted to another career; It also makes us do more and better work.

**#12**

1. ስለ አንተ እና ኤች አይቪ ጉዳይ ያለህበትን ሃላፊነት ብትነግረኝ

እድሜየ 42 ሲሆን በኤች አይቪ ጉዳይ ፎካል ፐርሰን አስተባባሪ እና ኮሚቴ አለ የዛ ኮሚቴ አስተባባሪ በመሆን ነው የምሰራው፡፡ የምንሰራው ስራ በዋናነት የመንገድ ስራ ስለሆነ ሰራኞች ተጋላጭ ከሆኑ የማህበረሰብ ክፍሎች አንዱ ስለሆኑ የምንሰራቸው፡፡ የስራ ዘርፎች ጥገና፣ ፕሮጀክት እና ሄድ ኦፊስ አሉ፡፡ በእነዚህ በሶስቱ ማዕከላት ዋናው ኮሚቴ ከዚህ ሁኖ በየደረጃው ደግሞ ኮሚቴ ተቁዋቁሞ ይሰራል፡፡ የምንሰራቸውሰ ስራዎች የግንዛቤ ችግር እኛ አለ ብለን አናስብም፡፡ ግን ያን ግንዛቤ ማስታወስ፣ የማስታወስ ስራ እንሰራለን፤ ከግንዛቤ አንጻር የምንሰራው ስራ ይሄንን ነው፡፡ በሁለትኝነት ድጋፍ ለሚያስፈልጋቸው ሰዎች የተለያዩ አይነት ድጋፎችን እንሰጣለን፡፡ በየወሩ በገንዘብም የምናግዛቸው አሉ፡፡ በተለይ አቅመ ደካሞችን ለይተን ከጤና ተቁዋማት ጋር በመነጋገር እነዚህ ተቁዋማት በሚሰጡን መረጃ መሰረት ቤተሰቦቻቸውን ላጡ በተለይ ህጻናት በየወሩ ድጋፍ እናደርጋለን፡፡ ቢያንስ እስከ 500 ብር፡፡ ትምህርቸውን ሲጀምሩ ለትም/ት የሚያስፈልጉ ቁሳቁሶችን እንገዛለን፡፡ አንዳንዴ ደግሞ ትልልቆች ሁነው መተዳደሪያ የሌላቸውን ቁዋሚ መተዳደሪያ እንዲኖራቸው፡፡ ለምሳሌ ፕሮጀክት ሳይቶች ወረዳዎች አካባቢቤቶችን በመስራት ከተማ አስተዳደሮች እስከፈቀዱ ድረስ እና መነሻ ካፒታል በመስጠት ስራ እንዲጀምሩ የማድረግ ስራዎችን ነው በአብዛኛው እኛ የምንሰራው፡፡

2. በዚህ ጉዳይ ምን ያክል ጊዜ ሰራህ ማለት ነው

እንግዲህ ከ2005 ዓ.ም ጀምሮ ነው፡፡

3. ስለዚህ ተቀናጅቶ የመስራት፣ ክትትልና ሪፖርት የመደራረግ ሁኔታ እንዴት ታየዋለህ

ጤና ቢሮ ጋር ተቀናጅተን ነው የምንሰራው፡፡ በተለይ ግንዛቤ ፈጠራ ላይ የጋራ መድረኮች ሲኖሩ እነሱን በማሳተፍ አፕዴት የሆኑ መረጃዎች እንዲተላለፉ፣ አሁን በምን ደረጃ ነው ያለ የሚለውንም በማሳወቅ በኩል ጤና ቢሮው በትብብር ነው እየሰራ ያለ፡፡ የትኛውም ቦታ በጠራናቸው ሰዓት በትብብር ነው ተባበረን የምንሰራ፡፡ በተጨማሪም ኮንዶም ስርጭት ላይ አንዱ የምንሰራው ነው፡፡ እሱን ስንሰራ ከእነርሱ ነው ሶርሳችን፡፡ ከጉልበት ሰራተኛ እስከላይ ሃላፊዎችን ያማከለ ስራ እንሰራለን፡፡

4. ያልተሰሩ ስራዎች አሉ ብለህ የምታስባቸው ይኖራሉ

ያልተሰሩ ስራዎች ፕሮጀክት ቦታዎች እሩቅ ስለሚሆን ከቫይረሱ ጋር የሚኖሩ ሰዎች መድሃትን በቀላሉ ማግኘት አይችሉም፡፡ በዚህ ምክንያት ሰራተኞቻችንን ቅድሚያ ሰጠን የተሻለ ቦታ ለማዛወር እንገደዳለን፡፡ በአቅራቢያቸው የተሻለ አክሰስ አለበት አካባቢ ሂደው አግልግሎት ማግኘት ስላለባቸው ማለት ነው፡፡

5. የኤች አይ ቪ ኤዲስ አገልግሎት እደ አጠቃላይ በእኩልነት ወይ በፍትሃዊነት ተደራሽ ከመሆን አንጻር እንዴት ትረዳዋለህ

ፍትሃዊነት ብየ አላስብም፡፡ የተሻሉ ከተሞች ላይ የተሻለ ተደራሽነት እና አገልግሎት አለ፡፡ ራቅ ያሉ ቦታዎች ላይ ደግሞ ተጠቃሚነት የለም፡፡ ስለዚህ ለሁሉም ማህረሰብ ፍትሓዊ ተደራሽ ነው ብሎ መናገር አይመስለኝም፡፡

6. ምክንያቶቹ ምን ሊሆኑ ይችላሉ

አንደኛው ችግር የምጠቅሰው በፊት እንደነበረው ትኩረት አሁን ለቫይረሱ ትኩረት መስጠት አለ ብየ አላስብም፡፡በፊት በቂ ትኩረት ይሰጥ ነበር፤ በዚያ ትኩረት ልክ ወደ ታች የመውረድ እድሉ ሰፊ ነበር ከነ ሊሚቴሽኑ ማለቴ ነው፡፡ ከዛ ውጪ ደግሞ ምናልባት በፊት የነበሩ ፈንዶች ሊሆኑ ይችላሉ፡፡ መንግስት በራሱ ብቻውን አይደለም እነዚህን ስራዎች የሚሰራቸው ብዙ ጊዜ፡፡ ስለዚህ እነዚህ ነገሮች ሲቀንሱ መንግስት ደግሞ ለዚህ ብሎ የሚበጅተው በጀት ስለሌለ ከበጀት አቅም እና በቂ ትኩረት አለመስጠት ያለውን ሪሶርስ ካለመመደብ ጋር ይያያዛል ብየ አስባለሁ፡፡

1. ከሰው ሃይል አንጻር፣ ከአመራር አንጻር እንደ ተግደሮት ሊቆጠሩ ይችላሉ

አዎ አንዱ ትኩረት የምልህ እሱን ነው፡፡ አመራር ከላይ ትኩረት ካልተሰጠው፣ በየደረጃ ያለው አመራር ትኩረቱ አነስተኛ ነው፡፡ ያው የኛ አገር ደግሞ ወቅታዊ ነው ሁሉም ነገር፤ ወቅታዊ ነው፡፡ እንጂ እንደተግባር ይዞ ስራ ከመስራት አንጻር ችግር አለ፡፡

1. ፍትሓዊ አሰራርን ስመዘርጋት የምትከተሉት መመሪያ ወይም ፖሊሲ አላችሁ

ግልጽ የሆነ ያወጣነው ፖሊሲ የለንም፡፡ጤና ቢሮ በሚሰጠን አቅጣጫ ካልሆነ በስተቀር በራሳችን ያወጣነው ሲስተም የለንም በሲስተም ደረጃ፡፡

1. ምናልባት ተጨማሪ ስለተግዳሮቶች የምትጠቅሰው ነገር ካለ

እንግዲህ አሁን አንደ ችግር አሁን ያለው አሰራር ከስትራክቸር ጀምሮ አንሱዋል፡፡ ስትራክቸሩ ካነሱ ትኩረቱም አብሮ አንሱዋል ማለት ነው፡፡ ከዚያ አንጻር የተነሳ ወደ ታች የሚሰሩት …እኛ አሁን ከኮንስትራክሽን አንጻር ብዙ ችግር ስሊሚያጋጥም ነው እንጂ ብዙ ቦታ የለም ማለት ይቻላል ስራውና ትኩረቱ፡፡ ስለዚህ መንግስት ከስትራክቸር ጀምሮ ከፋይናንስ ጀምሮ ድጋፍ ስለሌለው ወይም ትኩረት ስለሌለው ይህን ስራ በትኩረት ከታች ድረስ ወርዶ ለመስራት አንዱ ችግር ነው ብየ ነው የማስበው፡፡ ዋናው ሶርስ ከዛ ላይ ነው፡፡ ሌሎቹ ይህ ከተፈታ እየተፈቱ የሚሄዱ ናቸው፡፡

1. ቢስተካከሉ የምትላቸው ወይም ወደፊት የመፍትሔ ሐሳብ የምትላቸው ካሉህ

ወደ ፊት ምናልባት ከበጀት አንጻር እያንዳንዱ ተቁዋም ካለው በጀት 2 ፐርሰንት መመደብ አለበት ይላል፡፡ ግን ይሄ በጣም ሊሆን የማይችል ነገር ነው፡፡ ሊሆን የማይችል ነገር ነው፡፡ አንደኛ በጀቱ በጣም ትልቅ ይሆናል፡፡ ለምሳሌ ለእኛ ሴክተር ከ1 ቢሊዮን በላይ በጀት ይመደባል፡፡ ከዚህ ውስጥ ሁለት ፐርሰንት እንዴት አድርገህ እንደምትበጅት አስበው እና አንዳንዴ የሚወጡ ህጎች ሊተገበሩ የሚችሉ አይነት መሆን አለባቸው፡፡ እኛ አንዱ እያስቸገረን ያለ ፓርት ይሄ ነው፡፡የምትሰሩትን ስራ ተጠቀሙ ያኔ ወጪ እናደርጋለን በሚል ነው የተስማማነው፤ ምንም ማድረግ ስለማይቻል፡፡ 12. መጨረሻ ላይ የምትለው ነገር ካለህ እኩል ያገልግሎቱ ተጠቃሚ ያልሆኑትንም ታሳቢ በማድረግ

ኤች አይ ቪ ላይ ብቻም ሳይሆን ጤና ሲስተሙ ከተማ ላይ ነው፤ ብዙው ነገር፡፡ የከተማው ሰው አማራጭ አለው፡፡ ገጠር ላይ ግን እንደዚህ አይነት አክሰስ የለውም፡፡ አቅም ቢኖረውም አገልግሎት በቀላሉ ለማግኘት የሚቸገሩበት ሁኔታ አለ፡፡ አብዛኛው የኢትዮጵያ ሕዝብ ገጠሬ ሁኖ ሳለ ግን አክሰስ ያለው ደግሞ ከተማ ላይ ነው፡፡ትኩረት ለገጠር ነው መሆን ያለበት ብየ የማስበው፡፡ አምራች ማህበረሰብ ያለበት ሌላውን ሁሉ የሚመግበው ገጠሬው ነው፡፡ ያ ጤናው ካልተጠበቀ ከእንደዚህ አይነት በሽታ መጠበቅ ካልቻለ የከተማውም ሰው መጎዳቱ አይቀርም እና ትኩረቱ የት ጋር ነው መሆን ያለበት የምትለው ነገር ግራ ነው የሚያጋባ፡፡

**#12**

1. Please tell me about you and your responsibility for the HIV issue

I am 42 years old and there is a focal person coordinator and a committee for HIV issues and I am working as the coordinator of that committee. As the work we do is mainly road work, workers are one of the most vulnerable sections of the community. The functional areas are Maintenance, Project and Head Office. In these three centers, the main committee works from here, and at each level, a committee works. We do not think that there is an awareness problem. But remembering that understanding, we do the work of remembering; This is what we do in terms of awareness. Secondly, we offer a range of support to people who need support. We also help them financially every month. We identify those who are particularly weak and talk to the health authorities, and based on the information provided by these authorities, we provide monthly support to especially children who have lost their families. At least up to 500 Birr. When they start school, we buy the materials they need for school. Sometimes they may be adults so that those who do not have a livelihood we support them so that they can have a real livelihood.

For example, project sites, we do the work of making the districts work as long as the city administrations allow them to start working by providing initial capital.

2. How much time have you worked on this matter?

Well, since 2005.

3. How do you see the state of coordination, monitoring and reporting?

We work together with the Health Bureau. Especially when there are joint platforms for creating awareness, the health office is working in cooperation by involving them so that updated information can be transmitted, as well as informing them at what level it is now. Wherever we are called, we work together in cooperation. We also work on condom distribution. Our source is from them when we do it. We work from the laborer to the managers.
4. Do you think somethings are not done? Because the project sites are remote, people living with the virus can't easily access medicine. As a result, we are forced to prioritize our employees and relocate them to a better location. It means that they have to go to an area with better access to get services.

5. How do you understand HIV/AIDS services to be universally or equitably accessible?

I don't think it's fair. There is better accessibility and service in better cities. There is no benefit in remote areas. Therefore, I don't think it is fair to say that it is accessible to all communities.

6. What could be the reasons for HIV/AIDS services inequalities?

One of the problems I will mention is that I don't think that we should pay attention to the virus as much as before. I mean, with that focus, there was a lot of opportunity to go down. Other than that, it may be interruptions of previous funds. The government is not alone in doing these things. Therefore, when these things are reduced, the government does not have the budget to allocate for this, so I think it is related to the budget capacity and not paying enough attention to not allocating the available resources.

7. How do you see from human resources and leadership perspective as a challenge?

Yes, one of the things I want to focus on is this. If leadership is not focused at the top, leadership at every level is less focused. Everything is current in our country; it is concentrated on the current issues. There is a problem in terms of working with it as a practice.

8. Do you have a guideline or policy to follow when establishing and delivering equitable HIV/AIDS services?

We don't have a clear policy. We don't have a system at the system level, except for the direction given by the health office.

9. Maybe if you mention additional challenges of equity?

So now one problem is the current system, starting from the structure.

If you do not put it at the structure level, you do not also give attention. From that point of this view, those who work down... We are not facing many problems in terms of construction, but there is almost no space for HIV and attention. Therefore, since the government does not have support or attention from creating better structure to finance, I think it is one of the problems to do this work from the bottom up. The main source is from there. The others will be resolved if this is resolved.

10. If you have any suggestions for improvement or future solutions, please.

In the future, may be in terms of budget, the Health Bureau said each sector should allocate 2 percent of the available sectors’ budget. But this is highly unlikely. It is impossible. First, the budget will be huge. For example, more than 1 billion budgets will be allocated for our sector. Think about how you're going to customize two percent of that, and sometimes the rules should be made in a way to be practised. This is the part that is bothering us. We agreed that you should use the work that you are doing and then we will do check and balance it later.

Because nothing can be done.

1. If you have something to say at the end, please.

Considering the non-users of equal services, not only on HIV, but also on the health system of the city. A lot of things. The city man has an alternative. But in rural areas, it does not have such access. Despite the potential, there are situations where it is difficult to get services easily. Most of the people of Ethiopia are rural, but the services are concentrated to the cities. I think the focus should be on the rural areas. It is the countryside that feeds everything else where there is a productive society. If he is not healthy, he cannot protect himself from this kind of disease, the city's people are bound to be affected and it is confusing what you say where the focus should be.

**#13**

1. የስራ ድርሻህንና ስለምትሰሩት ዝርዝር ስራዎች ብትነግረኝ

የኤች አይ ቪ ሜኒስትሚንግ ስራ ነው የምሰራው፡፡ የመንግስት መስሪያ ቤቶች ኤች አይ ቪ ኤዲስን የስራ ድርሻቸው አድርገው እንዲሰሩ የማስተባበር ስራ ነው የምንሰራ፡፡ በክልል ደረጃ ወደ አስራ ሁለት የሚሆኑ ዋና ወይም ስትራቴጂክ ሴክተሮች አሉ፣ ሌሎችም አሉ፡፡ አጠቃላይ በክልል ከስድስት ሺህ አንድ መቶ በላይ መስሪያ ቤቶች ሜኒስትሪሚንግ እዲሰሩ ማስተባበር ነው፡፡ እነዚህ ሴክተሮች በሙሉ ዓመታዊ ዕቅድ ያቅዳሉ፣ ዓመታዊ በጀት ይመድባሉ፣ ቢያንስ በወር አንድ ጊዜ ስለ ኤች አይቪ ይወያያሉ ማለት ነው፡፡ የ2030 ግብን ለማሳካት ተጋላጭ የማህበረሰብ ክፍሎችን በተመለከተ ከግሎባል ፈንድ የምናገኘው እርዳታ አለ፡፡ መንግስት በጀት ይበጅታል በየተቋማቱ፡፡ እያንዳንዱ መስሪያ ቤት ሁለት ፐርሰንት ይበጅታል፡፡ ስለዚህ በክልላችን ሲታይ ከ20 ወይም 30 ሚሊያን የሚጠጋ ብር ይሆናል የሁሉም ቢሮዎች ሲታይ፡፡ ስለዚህ ሀብት አለ፡፡ ያለውን ሀብት ተጠቅመህ ለሰራተኛህ ቢያንስ በወር አንድ ጊዜ እንዲወያዩ ይደረጋል፡፡ የሚወያዩበት ማንዋል አላቸው፤ ስልጠናም እናሰጣለን፡፡ እንደ ሁኔታው በየሩብ ዓመት ወይም ግማሽ ዓመት ሪፖርት ያቀርባሉ፡፡ እያንዳንዱ መስሪያ ቤት ጥንካሬና ደካማ ጎን እንገመግማለን፡፡ እንደ አጠቃላይ ጥሩ እንቅስቃሴ አለ፡፡ በተጨማሪ ቫይረሱ በደማቸው ያለባቸውንና ወላጅ አልባ ህጻናትን በገንዘብ ድጋፍ ይደረጋል፡፡ በክልላችን ብቻ ወደ 538 ልጆችን እናሳድለን፤ ጤና ቢሮው በራሱ ወደ 38 ልጆችን እናሳድጋለን፡፡ ሰራተኛው ከደሞዙ 1 ፐርሰንት ያዋጣል፡፡ አሁን አሁን እያስቸገረ የመጣው ነገር ምንድን ነው የትምህርት ተቋማት መቆራረጥ አለ (ወቅታዊ የፖለቲካ አለመረጋጋት እና ጦርነቱ)፡፡ እንደምታየው በወቅታዊ ጉዳይ ምክንያት ትምህርት ይዘጋል፤ ዪኒቨርሲትን ጨምሮ፡፡ ባለፈው ሁለት ዓመት የነበረው እንቅስቃሴ ቀንሷል፡፡ አንዱ ፈተና የሆነብን እርሱ ነው፡፡ ኮሎጆችና ዩኒቨርሲቲዎች ደረጃ የደረሱ ወጣቶች ደግሞ ለበሽታው ተጠቂ ናቸው፡፡ ሌላው ፈተና የሆነው በዩኒቨርሲቲ ውስጥ ሴት ተማሪዎችን ብር ካለው ሰው ጋር የሚያገናኙ ደላሎች መኖራቸው ነው፡፡በክልላችን ደግሞ ብዙ ተፈናቃይ መኖር ሌላው ፈተና ነው፡፡ ሌላው በሴክተሮች የመጣው ፈተና ሰዉ እናውቃለን ባይ ነው፤ ነገር ግን ደግሞ ሲጠነቀቅ አታየውም፡፡ ሴክተር መስሪያቤቶችም ሆነ እኛ የተረዳነው ነገር ቢኖር የሰው ስለዚህ ቫይረስ መስማት ሰልችቶታል፡፡ መስማት የሰለቸው ነው የሚመስል፡፡ እዲሁም ኤች አይ ቪን አቅልሎ የማየት ነገር ይስተዋላል፡፡ እናም በዋናነት በበሽታው ተጠቂ ናቸው ተብለው ሚገመቱ የማበረሰብ ክፍል ላይ አተኩረን እንሰራለን፡፡

1. በማህበራዊ ህይወታቸው ዝቅ ያሉ የማህበረሰብ ክፍልን ከመድረስ አንጻር የምትሰሩት ስራ ካለ ብትነግረኝ

ዋና እና ቁልፍ የሚባሉ የማህረሰብ ክፍሎች አሉ፤ እና እነዚህን የማህረሰብ ክፍሎች በተለይ ሴተኛ አዳሪዎች ላይ እንሰራለን፡፡ በክልላችን ወደ 84 ከተሞች ላይ ሴተኛ አዳሪዎችን እናሰለጥናለን፤ ከእነሱም ምርመራ ያደርጋሉ፡፡ በሽታ የተገኘባቸው ፀረ-ቫይረስ መድኃኒትእንዲጀምሩ ይደረጋል፡፡ እንዲሁም ከተለያየ ኢንዱስቱሪ ላይም ተመሳሳይ ስራዎችን እንሰራልን፡፡ የህይወት ክህሎት፣ የአቻ ለአቻ ስልጠና ደግሞ ተማሪዎች ላይ አዝወትረን እንሰራለን፡፡

1. ተጠቂ የማህበረሰብ ክፍሎችን ለይቶ ከመስራታችሁ በተጨማሪ ለኤች አይ ቪ ቫይረስ አገልግሎት ተደራሽ ያልሆኑ የማህረሰብ ክፍልን ፍትኃዊ በሆነ መልኩ ተደራሽ ከማድረግ አንጻር ስለምትሰሩት ስራ ብትነግረኝ

ዋና ትኩረታችን ለበሽታው ተጠቂ የሆኑ የማህበረሰብ ክፍል ላይ ስልጠና በማሰልጠን ምርመራ እንዲያደርጉ ነው፡፡ እነዚህም ሴተኛ አዳሪዎች፣ የትዳር አጋር የሞቱባቸው፣ ተማሪዎች ላይ በትኩረት ይሰራል፡፡ ዋና ትኩረቱ ሶስቱ 95ን ከማሳካት አንጻር ነው የምንሰራ፡፡

1. ይህን ሁሉ ስትሰሩ ያልተሰሩ ስራዎች የምትላቸው ካሉ ብትነግረኝ

ስለ ኤች አይ ቪ ማውራት ከጀመርን ከ30 ዓመት በላይ ስለሆነው ሰው መስማት ሰልችቶታል፡፡ ሰው እናውቀዋን ነው የሚልህ፡፡ በእርግጥ ጥናቶች እንደሚያሳዩት 96 ፐርሰንት 98 ፐርሰንቱ ስለኤች አይቪ መረጃ አለው ይላል፤ ነገር ግን ጠቅላላ እውቀቱን ብትለካው ባዶ ነው፡፡ ስለዚህ መስማት ነው እንጂ ተግባራዊ አናደርግም፡፡ ተላምደነዋል፡፡ አንዱ ፈተና ይህ ነው፡፡ የምናስተምርበት ሳይንሱ አልተቀየረም፡፡ አንድ ነው፡፡ ኤች አይ ቪ ማለት ይህ ነው፣ ምልክቱ እንደዚህ ነው፣ በዚህ ነው የሚተላለፍ፣ የምትለው ነገር ሳይንሱ አልተቀየረም፡፡ ሰው ግን ይህ ነገር 30 ዓመት አውርተነዋል፡፡ ይህን ነገር ተናግረን ለውጥ አናመጣም ይላል፤ እኛ ደግሞ ከየት እናምጣ፡፡ አዲስ የማወያያ ወይም የማሰልጠኛ ዘዴ አምጡ ነው የሚሉህ፡፡ ለምሳሌ ይህን ማወያያ (በቀኝ እጁ አነስቶ እያሳየኝ) ሁለት ቀን አወያየህበት እንበል ለሶስተኛ ጊዜ ያንኑ ሰው ብትደግመው ምን ይልኃል…እ.. እኛ የተቸገርነው ፈተና ይህ ነው፡፡ እኛ በየጊዜው ማንዋል እንቀይራለን፡፡ ነገር ግን የምንቀይረው ዓመተ-ምህረቱንና አንዳንድ ቁጥሮችን፣ ለምሳሌ ቫይረሱ በደማቸው ያለባቸውን፣ እንጂ የስልጠና አሰጣጡን ሂደት አይደለም፡፡ ዛሬ እንደምታየው ሰው ድህነት ላይ ነው፡፡ እና የኮንዶም ስርጭት ስለቀነሰ እና በራስ አቅም በመግዛት ስለሆነ ሰው በኑሮው ተስፋ የመቁረጥና አጋጣሚውን ታገኘም ያለጥንቃቄ ወሲብ ይፈጽማሉ፡፡ ይህ እኛ በጥናት ያገኘነው ነው፡፡ ሰዎችን ጠይቀን የነገሩን ነገር ነገ በርሃብ ልሞት እንዴት ነው ስለበሽታ መከላከል የማስበው ይላሉ፡፡ ሌላው በመሪዎች ደረጃ መፋዘዝ አለ፡፡ ይህ ደግሞ በወቅታዊ ሁኔታዎች ላይ መጠመድ ያመጣው ጣጣ ነው፡፡ ትልቁ ፈተና ያለው ሰው ላይ ነው፡፡ ሰው መስማት አይፈልግም፤ አቅልሎ ነው የሚያየው፡፡ ስንት ገዳይ በሽታ እያለ ስለዚህ ታወሩናላችሁ ይላል፡፡ ካንሰር፣ የደም ግፊት የመሳሰሉትን ነው ሰው እየፈራ ያለ፡፡

1. በተጨማሪም ተደራሽ ያልሆኑ ነማህረሰብ ክፍልን ገጠር ያሉትን ጨምሮ ፍትኃዊ አገልግሎት እንዲያገኙ ለማድረግ የሚያጋጥማችሁ ተግዳሮት ካለ ብትነግረኝ

ተደራሽ ያልሆኑትን ልንገርህ… ለምሳሌ እኛ ገጠርም ሆነ ከተማ ጤና ኤክስቴንሽን ባለሙያዎች አሉን፡፡እነሱ ከሚሰሩዋቸው የኤክስቴንሽን ፓኬጆች ውስጥ አንዱ ስለ ኤች አይ ቪ ነው፡፡ እንዲሁም ጤና ጣቢያዎችንም እንጠይቃለን እነሱም የሚሰሩትን ስራ ይነግሩናል፡፡ አንዳንድ ጊዜ በጀት እያለም አጠቃቀሙ ላይ ችግር አለ፡፡ የተመደበውን በጀት ከተመደበበት ዓላማ ውጪ መጠቀም… ለምሳሌ በስራ ቦታ ሰዓት መድቦ ከመወያየት ይልቅ ወደ ሌላ ቦታ ሂደው አበል ማወራረድ…ባህር ዳር ያለ ሰራተኛ ባህር ዳር አልሰለጥንም ይላል፤ እና ሌላ ዞን ወስደው እኛን ይጠሩንና ስጠናውን እንሰጣለን፡፡

ሌላው ግን ክትትል እናደርጋለን፡፡ በፊት አርሶ አደር ላይ ይሩ ጥንቃቄ ነበር፤ አሁን ግን ገጠሩ ማህበረሰብ ውሽማ መያዝ፣ ወደ ከተማው መጥቶ ጥንቃቄ የጎደለው ወሲብ መፈጸሙ ትንንሽ የገጠር ከተሞች ላይ ቫይረሱ እየተዛመተ ነው፡፡ ለላው ግንዛቤ ፈጥረን ምርመራ የሚደረግ ጥቂት ሰዎች ላይ ነው፡፡ ምክንያቱም ትልቅ የአቅርቦት ችግር አለ፡፡ አዲስ ስትቴጂ አውጥተን በማስተማሩም በማሰልጠኑም በኩል ያስፈልገናል፡፡

1. ፍትሐዊ የኤች አይ ቪ አገልግሎት ለመስጠት ወደ ፊት መስተካከል አለባቸው የምትላቸውን ነገሮች ብትነግረኝ

የጸጥታውን ችግር መፍታት ያስፈልጋል፡፡ የአጀንዳዎች መደራረብ ላይ ቅደም ተከተል ማውጣት ብንችል፡፡ ከመንግስታዊ ካልሆኑ ድርጅቶች መጥፎ ነገር ያስተማሩብን ነገር አለ፡፡ ስልጠና ሲሰጡ አበል እከፈሉ ነበር፣ ዛሬ እነሱ ሂደዋል፤ ስልጠና እናሰልጥን ስንል አበል ነው የሚጠይቅህ ሰው፡፡ ምክንያቱም ከእነዚያ ድርጅቶች ለምዶ ነው፡፡ በጤናው ዘርፍ ያሉ ሙያተኞችም ለሙያቸው ያለው ትኩረት እየቀነሰ መምጣቱ አንዲ ሊሆን ይችላል፡፡ ገንዘብ እንዴት አገኛለሁ፤ ምን ብሰራ የተሻለ ገቢ አገኛለሁ ነው የሚል፡፡ ሰው አድናለሁ የሚለው ቃል እየቀነሰ ነው፡፡

1. በመጨረሻ የምትለው ነገር ካለህ

ወጣቶች ላይ ተጨማሪ ጥናቶችን ብታጠኑ፡፡ የምታጠኑትን ስራ ለእኛ ሕዝብ በሚጠቅም መልኩ፣ እውነተኛውን ችግር ነቅሶ በማውጣት ችግርን መፍታት፡፡ከእናንተ ይህን እጠብቃለሁ፡፡

**#13**

1. Tell me about your role and the detailed activities.

I work to HIV mainstreaming. We are working to coordinate government offices to make HIV/AIDS their work. There are about a dozen core or strategic sectors at the regional level, and there are others. It is to coordinate more than six thousand and one hundred offices in the region to do the mainstreaming. This means that all these sectors plan an annual plan, allocate an annual budget, and discuss about HIV at least once a month. There is help from the Global Fund for vulnerable communities to achieve the 2030 goal. The government budgets for each institution. Each office allocates two percent. Therefore, in our region, it will be around 20 or 30 million Ethiopian Birr if all the offices are set 2 percent budget. So, there is wealth. Use the resources available to your staff to discuss at least once a month. They have manuals to discuss. We also provide training. They report quarterly or semi-annually. We evaluate each office's strengths and weaknesses. There is good activity in general. In addition, financial support will be given to orphans and children with HIV. In our state alone, we have about 538 children; The health office itself raise about 38 children. The employees contribute 1 percent of his/her salary. Young people who have reached the level of colleges and universities are also susceptible to the disease. Another challenge is that there are dealers in the university who connect female students with men who have money. Another challenge is the presence of many displaced people in our region. Another challenge that came from the sectors is that people think as they know about HIV. But you also don't see them being careful. Both the sector offices and we understand that people are tired of hearing about this virus. It seems they are tired of hearing it. There is also an underestimation of HIV. And we mainly focus on the part of the population that is considered to be affected by the disease.

1. Please tell me if there is any work you do in terms of reaching the lower social class

There are core and key segments of society; And we work on these sub-groups specifically on prostitutes. We train prostitutes in 84 cities in our region; They will also investigate from them. Those diagnosed with the disease will be started on antiviral medication. We also do similar work for different industries. We also provide life skills and peer-to-peer training to students.

1. In addition to identifying vulnerable communities, tell me about the work you how you deliver HIV services fairly.

Our main focus is to train and offer HIV testing to the vulnerable sections of the community. These include prostitutes, those whose spouses have died, and students. The focus is on achieving the three 95.

1. When you do all this, please tell me if there are any tasks that you don't do?

People are tired of hearing about HIV that we start streaming for over 30 years. Someone who tells you that they know it. In fact, surveys show that 96 percent, 98 percent say they have information about HIV; But if you measure comprehensive knowledge, it is empty. So, we hear it, but we don't do it. We are used to it. This is one challenge. The way we teach has not changed. It is one. This is what HIV is, this is the symptom, this is how it is transmitted, what you say, the science has not changed. We've been talking about this for 30 years. People say that we will not make a difference if we say this. Where do we get it from? They tell you to come up with a new method of discussion or training. For example, let's say you moderated this moderator (he raised his right hand and showed me) for two days. If you repeat the same person for the third time, what will he say? We change manuals regularly, but we are changing some numbers, such as HIV prevalence, not the training process. As you can see today, people are in poverty. And because the distribution of condoms has decreased and people are buying them on their own, people get desperate in their lives and have sex without caution. This is what we found in our investigation. When we asked people what they told us, they said, "How am I going to die of hunger tomorrow? I think about disease prevention." Another is confusion (መፋዘዝ) at the leadership level. This is the problem of being occupied with current problems. The biggest problem is on the people. People does not want to hear about HIV; people take it simple. They say that while there are so many deadly diseases, why you talk to us about HIV. People are afraid of cancer, high blood pressure rather than HIV.

1. Please tell me any challenge you face in delivering equitable services for the inaccessible people, including the rural ones?

Let me tell you the ones that are not accessible... For example, we have both rural and urban health extension workers. One of the extensions packages they do is about HIV. We also ask health centers, and they tell us what they do. Sometimes there is a problem with the use of the budget. Using the allocated budget out of the purpose for which it was allocated... For example, go to another place and instead of allocating time and discussing at workplace... some say that an employee at Bahr Dar will not be trained at Bahr Dar because they will not get per diem if training is given at workplace; And they invite us when the give training out of Bahir Dar and we go with them to give training. But the other one we monitor. In the past, farmers were cautious. But now the rural community is being attacked, people coming to the city and having unprotected sex, the virus is spreading in small rural towns. It is only a few people who are happy to be tested for HIV after many have received awareness because there is a huge supply problem. We need a new strategy in teaching and training.

1. Please tell me somethings you think that should be done in the future to provide equitable HIV/AIDS services.

The security issue needs to be addressed. It would better if could sort out the order of agendas. There are bad lessons from non-governmental organizations. They used to pay allowances or per diem when they gave training, today they are gone; When we say we are going to train, it is a person who asks you for an allowance or per diem. Because he is used to those organizations. Professionals in the health sector may also be losing attention to their profession. Professionals are thinking on how do I earn money? Whatever I do, I will get better income. The word "I will save people" is falling.

1. Finally, if you have something to say please.

We need more studies on young generation. I expect this from researcher by finding out the real problem and solving the problem that benefits the people.

**#14**

1. ኤች አይ ጉዳይ በተመለከተ ስለአለብዎ ሃላፊነት ይንገሩኝ

በኔትወርክ ስር የግንዛቤ ማስጨበጫ ስራዎችን፣ የምርመራ ስራዎችን፣ አድሎና መገለል እንዲቆም የግንዛቤ ማስጨበጫ ስራዎችን፣ የምግብ ድጋፍ፣ በኢኮኖሚ እርዳታ ለሚያስፈልጋቸው በማገዝ፣ ስልጠና መስጠትና እራሳቸውን ወደ እሚችሉበት የስራ ዘርፍ በማሰማራ በኩል የእኔ ተሳትፎም የጎላ ነበር ማለት እችላለሁ፡፡

1. እባክዎ ኤች አይ ቪ አገልግሎትን በተሳለጠ መንገድ ለመስጠት የምትጠቀሙበት ዘዴዎች ቢነግሩኝ

መንግስት አካላት ጋር መምከር፣ መነጋገር፣ ችግሮችን ማሳየትና መፍትሄ ማስቀመጥ እና አግልግሎት የማያገኙ ግለሰቦች ካሉ አገልግሎቱን እንዲያገኙ ማድረግ ናቸው፡፡በአንድም ሆነ በሌላ ሁኔታ ማህበራት እርስ በእርስ ድጋፍ ቡድን የሚባል አለ አስርም ሀያም አባላትን በማደራጀት በቡድን በቡድን እንዲወያዩ እና እንዲመካከሩ በማድረግ” ችግር ካለ ችግሩን ያወጣሉ፣ ችግሩ መፍትሔ ሰጪ አካላት ጋር መወያየትና ግንዛቤ የማስጨበጫ ስራዎች ይሰራሉ፡፡

1. የምትሰጡት አገልግሎት እስከ ወረዳ እና ገጠር ምን ያክል ተደራሽ ነው

እውነት ነው የእኛ ተቁዋም ጤና ተቁዋማትና ሆስፒታሎች ኬዝ ማናጅመንት ላይ ፀረ-ኤች አይ ቪ መድሃኒት በሚሰጥት ሁሉ ስናግዝ ነበር እናም በክልሉ 263 ማህበራት እነሱም የእኛ አካል ናቸው፡፡ ስለዚህ አገልግሎቱ ወይም መዋቅሩ እስከ ወረዳ ድረስ የምንደረስበት ሁኔታ አለ ማለት ይቻላል፡፡ ነገር ግን የሐብት ውስንነት ስላለ ሁሉንም ማዳረስና የሚፈለገውን ውጤት ለማምጣት ግን ከባድና አስቸጋሪ ነው፡፡ የሰው ሓይል ቀጥረን በበጀት የምንደግፋቸው ትልልቅ ከተሞች ላይ ወደ 41 የሚሆኑ ከተሞች ላይ እንሰራለን፡፡ የምርመራ ስራውን ደግሞ ሌላ 36 ቦታዎች ላይ እንሰራለን፡፡

1. ውስንነት ቢኖርም ለሰራተኞች ስልጠና መስጠትና ክትትል ማድረግ ያለው ሁኔታ ምን ይመስላል

ከዚህ በፊት አጋር አካላቶች ብዙዎች ስነበሩ ልዩ ልዩ ስልጠናዎች እና ክትትል ይደረግ ነበር፡፡አሁን ግን ከጊዜ ወደ ጊዜ እየቀነሰ ነው፡፡ድጋፍና ክትትሉም በጠነከረ መልኩ እየተሰጠ ነው ማለት አይቻልም፡፡ነገር ግን ለተወሰኑ በጀት የሰጠናቸውን በሪቪው ሚቲንግ ስንሰበሰብ ሪፖርት በማቅረብ እንገመግማለን፡፡

1. ግብረ-ሰናይ ድርጅቶች ሲቆሙ እነእሱ ሱሰሩት የነበረውን እንዴት ነው የምታስቀጥሉት

በእርዳታ ላይ የተንጠለጠሉ ስራዎች ቅድም እንዳልሁት አጋር ድርጅቶች ስራቸውን ሲያቆሙ ይቀዛቀዛል፡፡ ወጣ ገባ የማለት ወይም ቀጣይነት በሆነ መንገድ መሸፈን ቢቸግርም በተወሰነም ቢሆን እንሞክራለን፡፡

1. ያልተሰሩ ስራዎች የሚሉዋቸው ካሉ

ማህበረሰቡ ይሰማል ግን ግንዛቤ ስራው የአስተሳሰብ ለውጥ በማህበረሰቡ ውስጥ አምጥቶ አድሎና መገለልን በሚገባው ልክ ለውጥ አምጥቱዋል ማለት አይቻልም፡፡ ስለዚህ በዚህ ሁኔታ አድሎና መገለልም ይቀጥላል፤ በሽታ ስርጭቱም ይቀጥላል፡፡

1. የኤች አይቪ አገልግሎት ተደራሽት ፍሓዊነት ወይም ኢፍትሓዊነት ምን ይመስላል ቢስረዱኝ

የህክምና አገልግሎት በዋናነት ቫይረሱ በደማቸው ያለባቸው ሰዎች መድሃታቸውን በተገቢው መልኩ እንዲወስዱ ማድረግ ነው፡፡ በተለያየ ምክንያት ቫይረሱ በደማቸው እያለ ከቤታቸው ተደብቀው ያሉ ደግሞ ብዙዎች ናቸው፡፡ይህ ደግሞ ፍትሓዊ ተደራሽነትን ጥያቄ ውስጥ ያስገባዋል፡፡ በጤና ሙያተኞች በኩልም ሙያው የሚፈቅደውን ስነ-ምግባር ተላብሶ አለመስራት ይታያል አልፎ አልፎ፡፡ አገልግሎት በተገቢው መልኩ የማይሰጥ ከሆነ ያ ተገልይ ይቀራል፣ ይሸሻል..እንደዚህ አይነት ነገሮች ይፈጠራሉ፡፡

1. የእርስዎ መስሪያ ቤት ፍትሓዊ ተደራሽነትን ከመተግበር እና ተቁዋማዊ ከማድረግ አንጻር እንዴት ነው

ሰስተኔብሊቲ (ቀጣይነት) ያለው ስራን በተመለከተ፣ ፓርትነርሽፕ፣ ቮለንተሪዝም ጋር የተያያዙ ስራዎችን እሰራለን፡፡ ፈሰስ በተደረገበት አካባቢ በየአራት ወሩ ግምገማ ይካሄዳል፡፡ የአካል ጉዳተኛ ሰዎችን፣ ሴቶችን፣ ወረዳዎች ላይ የሚሰሩ ስራዎችን ለማህበራት እንሰጣለን፡፡ በሽታው በብዛት የሚገኝበትን ነው የሚያተኩር፡፡

1. ፍትሓዊ ተደራሽነትን ለመስራት ተግዳሮት

መሰረታዊ ችግር ፋይናንስ ችግር፣ የሰው አመለካከት ችግር (አገልግሎት ሰጪዎም አገልግሎት ተቀባዩም) ፈተና ነው፡፡ ለምሳሌ በጀት ከመመደብ አንጻር የሚመለከተው የቢሮ ሓላፊ ከራሱ ኪስ አውጥቶ የሚሰጥ ይመስል እንደፈለገ ተጨባጭ ባልሆነ መረጃ መመደብ እና የአገልግሎቱ ተጠቃሚዎች የኔ ናቸው ብሎ አለመውሰድ አንዱ ችግር ነው፡፡

ድጋፍ አድራጊ ግለሰብም ሆነ ድርጅት ችግሩ የኔም ነው ብሎ ከማሰብ ይልቅ እሱን እንደ መጽዋተኛ ሌለው ተመጽዋች አድርጎ የማየት እና እራሱን የመኮፈስ ሁኔታዎች ይስተዋላሉ፡፡

ጦርነት ያለባቸውን አካባቢዎች፣ የተፈናቀለ ማህረሰብን ከመድረስ አንጻር እንዴት ነው የምትሰሩ.. በጣም አስቸጋሪ ሁኔታ ነው፡፡ ቫይረሱ በደማቸው ያሉ ሰዎች ሳይቀር አድራሻቸው ይጠፋህና ቫይረሱ እንዲሰራጭ ይሆናል፡፡ በጦርነት ባለበት አካባቢ እኛም አንሄድም፡፡ ምክንያቱም ጦርነቱ የብሔር ስለሆነ እኛንም ቢያገኙን ስሚገድሉን ጦርነት ቀጠና የሆነ አካባቢ አገልግሎቱን አያገኝም፡፡ የመከላከል ስራ ከመስራት፣ ኮንዶም ስርጭት፣ መድሃኒት አቅርቦት ይቆማል፤ በሽታው በእጅጉ እንደሚሰራጭ ነው የሚሰማን፡፡

የማህበረሰቡ ባህል እሴት እንዴት ነው. ባህል፣ እሴቱ ወጉ ከባድ ነው፡፡ሰው ተመርምሮ ራሱን አውቆ ቫይረሱ በደሙ ሲገኝ እንደ ነውር ይቆጠራል፡፡ ለምሳሌ ባልዮው ተመርምሮ በደሙ ሲገኝ ሚስት አልመረመርም ትላለች፣ ሚሰት አውቃ ባል ተመርመር ሲባል አልመረመርም ይላል፡፡ ይህ እስከ አሁን ድረስ ትልቅ እንቅፋት ነው፡፡

1. ወደ ፊት ቢስተካከል ወይም ቢሰራ የምትሉት ካለ

ከውጭ እርዳታ ብቻ በሚገኝ ችግሩን እንፈታለን የሚል እሳቤ አመራሩ ቢያስወግድ እና ችግሩ የእኛ ስለሆነ ችግሩን እኛ እንፈታዋለን የሚል እሳቤ ውስጥ ብንገባ ጥሩ ነው፡፡ በሁለተኛ ደረጃ በክልላችን ታይቶ በማይታወቅ ሁኔታ ሀሽሽና ጫት አለ፡፡ እኛ ስናድግ ጫት ከአለበት አካባቢ ቢኖር በጣም እርቀን ነው የምንሄደው የነበር፡፡ አሁን ግን የተጋነነ ነው፡፡ ስለዚህ ጫትና ሐሽሽ ባለበት አካባቢ በሽታው ይዛመታል፡፡ ፍትሓዊ አገልግሎት ለመስጠት ተግዳሮት ይሆናል፡፡ የስራ ቅጥርን ለመጨመር የተለያዩ አማራጮችን ማስፋት እና ከኮሌጅ ወይም ዩኒቨርሲቲ የሚመረቁትን ከተቀጣሪነት አስተሳሰብ እንዲወጡ ስራዎች ቢሰሩ ሩ ነው፡፡ ምክንያቱም የሚቀመጠው ሲበዛ ወደ አልባሌ ተግባር ይገባል፡፡ እና የሚፈለገውን አግልግሎት ለመስጠት ለእኛም ሁሉን መድረስ እየከበደን ይመጣል፡፡ ምርምርህ ወደ ፖሊሲ ሰዎች የሚደረስ ከሆነ በጣም ትልቅ ነገር ነው እና አኔም ተስፋ አለኝ፡፡

**#14**

1. Tell me about your responsibilities regarding HIV

I can say that my participation was significant through network awareness activities, HIV testing activities, awareness activities to stop discrimination and stigma, food support, helping those who need economic assistance, training and deploying them to work areas where they can help themselves.

1. Please tell me the methods you use to provide HIV services in an easier way

Advising, talking with government bodies, showing problems and putting solutions and making sure that if there are individuals who are not getting services, they can get the services. In one or the other situation, there is a mutual support group called "Association" which organizes members to discuss and consult as a group. Discussions and awareness raising activities will be done with solution providers.

1. How accessible are the services you provide to the district and rural areas?

It is true that our institution, Health institutions and Hospitals have been helping in the case management of all those who provide anti-HIV drugs and 263 associations in the region are also part of us. Therefore, the service or the structure can be said to reach up to the district. However, due to limited resources, it is difficult and difficult to deliver all of them and achieve the desired results. We work in about 41 cities in the big cities that we hire manpower and support with the budget. We will do the investigation work in another 36 places.

1. Despite the limitations, what is the condition of providing training and monitoring to employees?

In the past, there were many partner organizations, so various trainings and monitoring were carried out. But now it is decreasing from time to time. It cannot be said that the support and monitoring is being given in a strong manner.

1. When humanitarian organizations stop, how do you continue the activities they started earlier?

Aid-Dependent Operations, as I mentioned earlier, when partner organizations stop working, it slows down. Although it is difficult to cover it in a way that is rugged or continuous, we will try at least to some extent.

1. If there are any unfinished works, please.

The community can hear it, but it cannot be said that the awareness work has brought about a change in the way of thinking in the community and brought about a change in discrimination and marginalization as it should. Therefore, in this situation, discrimination and marginalization will continue. The disease continues to spread.

1. What is the fairness or unfairness of access or delivery to HIV services?

Medical care is mainly to ensure that people with the virus in their blood take their medicine properly. For various reasons, there are many people who are hiding from their homes while the virus is in their blood. This puts fair access into question. It is sometimes seen that health professionals do not work according to the ethics allowed by their profession. If service is not provided in a proper manner, that individual will remain, run away…. such things will happen.

1. How is your office in terms of implementing and promoting equitable HIV/AIDS services?

Regarding the work with sustainability, we do work related to partnership, volunteerism. A review is conducted every four months in the area where the investment has been made. We donate to associations working on disabled people, women, districts. It focuses on where the disease is most prevalent.

1. Please tell me if you face challenge of making equitable HIV/AIDS services.

The basic problem is financial problem, human attitude problem (both your service provider and service receiver) is a challenge. For example, in terms of allocating a budget, one of the problems is that the head of the office who is concerned think that he is spending money out of his own pocket, allocating with unrealistic information and not assuming that the users of the service belong to him. Instead of thinking that the problem is mine, whether the supporting individual or the organization sees him as a beggar without a beggar (እሱን እንደ መጽዋተኛ ሌለው ተመጽዋች አድርጎ የማየት እና እራሱን የመኮፈስ ሁኔታዎች ይስተዋላሉ), situations are observed.

1. How do you work in terms of reaching war-torn areas and displaced communities?

It's a very difficult situation. Even people who have the virus in their blood will lose their address and the virus will spread. We will not go to a war zone. Because the war is ethnic based, even they may kill you if they get us, they will kill us, and an area that is a war zone will not get the service. Preventive work, condom distribution, medicine supply will be stopped; We feel that the disease will spread widely.

1. How do you see the value of, the culture of the community in provision of equitable HIV/AIDS services?

Culture, values ​​and tradition are challenging. When a person is tested positive to HIV, it is considered shameful. For example, when the husband is tested positive, the wife says she does not to be tested for HIV. This has been a major obstacle until now.

1. If there is anything you would like to say, please.

It would be good if the management gets rid of the idea that we can only solve the problem with help from outside and that we can solve the problem because it is our problem. Secondly, there is Hashish and khat which is unprecedented in our region. When we were growing up, if there was an area where there was khat, we would go very far away. But now it is highly prevalent. Therefore, the disease is spread in the area where there is khat and Hashish. It will be a challenge to provide equitable service. Expanding the variety of options to increase employability and working to get college or university graduates out of the employability mindset. Because the more unemployed or not have work, the more they engaged in unsafe sexual practice.

**#15**

1. ስለ እራስሽ እና በኤች አይ ቪ ጉዳይ የምትሰሩት ስራ ንሪኝ

በዋናነት በኤች አይ ቪ ጉዳይ የግንዛቤ ፈጠራ ስራዎችን እንዲሰሩ ነው የምናደርገው፤ ማለትም በትምህርት ቤቶች፣ በማህበረሰቡ፣ እና በዩኒቨርሲቱው ሰራተኞች እንዲሁም ተማሪዎች ላይ እንሰራለን፡፡ ዝርዝር ስራዎችን ለመጥቀስ ያክል ስልጠናዎችን ማስተባበርና መስጠት፣ ወጣቱ መጥፎ ልማዳዊ ድርጊትን በተመለከተ (አደንዛዥ ዕፅ፣ ቁማር፣ ጫት) በስልጠና፣ በማወያየት፣ በግሩፕ ካውንስል ወይም በኢንዲቪዱዋል ካውንስል ሊሆን ይችላል በዚህ ዙሪያ እንሰራለን፡፡ ፀረ-ሱስና ፀረ-ኤች አይቪ ክበባትን አቁዋቁመን እንደግፋለን፣ እናጠናክራለን፡፡ ተማሪዎችም እርስ በእርሳቸው እንዲወያዩ፣ እንዲመካከሩ፣ በችግሮቻቸው ዙሪያ እንዲወያዩ እናበረታተለን፡፡ ለምሳሌ የአቻ ግፊት ከፍተኛ ነው የኒቨርሲቲ ላይ፡፡የአቻ ግፊት ወደ መጥፎ ሁኔታ እንዳይራቸው ፒር ቱ ፒር ስልጠና እናካሂዳለን፡፡ በየግቢው ቢያንስ 40 ተማሪ እናሰለጥንና እነሱ ቀሪዉን ተማሪ እንዲደርሱት እናደርለን፡፡ለምሳሌ ከሰለጠኑት 40 ዎች አንዱ ተማሪ 10 ተማሪ ሊይዝ ይችላል፡፡ ላይፍ ስኪል ስልጠናም እንሰጣለን፡፡ ሲስተር ቱ ሲስተር ኤች አይ ቪ ቫይረስን ከመከላከል አንጻር ሴት ተማሪዎች ላይ ይህን እናደርለን፡፡ከመከላከል አንጻር በአብዛኛው አጋላጭ የሆኑ ጉዳዮች ላይ ነው የምንሰራ፡፡ ምሳሌ፣ ብሮሸር በአደንዛዥ እጽ ዙሪያ እናሰራጫለን፡፡ ከመቆጣጠር አንጻር በአብዛኛው ቫይረሱ በደማቸው ያሉ ሰዎችን ለማግኘት በየግቢው ምርመራ እናካሂድ ነበር፡፡ ነገር ግን በዚህ ዓመት የምርመራ ኪት አጣን እና ማስቀጠል አልቻልንም፡፡ ሌላው ግን ኮንዶም እናሰራጫለን፤ የኮንዶም ማስቀመጫ ሳትጥኖች አሉን፡፡ ኮንዶም ከተለያዩ አጋር አካላት ነው የምመጣው፡፡

ሌላው ከመምህራን፣ አስተዳደር ሰራተኛው እና ተማሪዎች በደማቸው ያለባቸው ተደራጅተው ሀብት እንዲያፈሩ ድጋፍ እናደርጋለን፡፡ በስና ልቡና ለማጠንከርም የተለያዩ ስልጠናዎችን እናዘጋጃለን፡፡ ለምሳሌ፣ ስለምግብ፣ በሽታውን ስመቆጣጠር፡፡ እንዲሁም የህግ ከለላ አገልግሎትም እንሰጣቸዋለን፡፡ ማህበረሰቡ ላይም ወላጆቻቸውን ያጡ ልጆችን በገንዘብ እናግዛለን፡፡

1. ሌላውን ማህረሰብ ተደራሽ ከማድረግ አንጻርስ ምን ስራ ትሰራላችሁ ከላይ ከጠቀሳችሁት ተጨማሪ

አሁን ማንሳት ካስፈለገ ከ2013 ጀምሮ ከኮረና መምጣት ጀምሮ ስራዎች እየተቀዛቀዙ ነው፡፡ 2002 ዓ.ም ላይ ታስክ-ፎርስ አቁዋቁመን ነበር፡፡ ከሁሉም የማህረሰብ ክፍሎች፡፡ ቀጥሎ በጦርነቱ ምክንያት ደግሞ አመራሩንም ማግኘት አልተቻለም፤ ሁላችንንም የጦርነቱ ክስተት፣ አለመረጋጋት፣ በጀት እጥረት፣ በሚገባ እንዳንሰራ ሁነናል፡፡

1. መተዳደሪያ ደንብ አላችሁ፣ በእቅዳችሁስ ያልተሰሩ የሚባሉ ስራዎች ካሉ

ያልተሰሩ ስራዎች አሉ፡፡ እንደሚታወቀው ጤና ነክ ጉዳዮች መንግስታዊ ባልሆኑ ድርጅቶች በብዛት በገንዘብ ይደገፋል፡፡ በተለይ ብዙ የኤች አይ ቪ ስራዎች መንግስታዊ ባልሆኑ ድርጅቶች ላይ የተንጠላጠለ ነው ማለት ይቻላል፡፡ ለምሳሌ ሲስታ ብትል እራሱን የቻለ ማንዋል አለው፤ ላይፍ እስኪል ስትል እራሱን የቻለ ማኑዋል አለው፤ፒር ኢዱኬሽን ብትል እራሱን የቻለ ማንዋል አለው፤ ይህ ሁሉ ጅማሬ የተጀመረ በኤንጅዎች ነው፡፡ ኤንጅዎች ገብተውም ውጭ ሁነው ይሰሩ ነበረ፡፡ እነዚህን ለማስቀጠል በጀት መንግስት መበጀት መቻል አለበት፡፡ አሁን በዩኒቨርሲቲው ነው ዝም ብሎ በመልካም እንትን ነው እንጂ ለኤች አይቪ ኤዲስ መከላከያና መቆጣጠሪያ ተብሎ የተቀመጠ ከላይ የወረደ በጀት የለም፡፡ ይህንን እያንዳንዱን ለማስቀጠል በጀት መኖር አለበት፡፡ ይህ ብቻ አይደለም ደሞዝ የሚከፈለው ሰራተኛም ሆነ ተማሪው የለመደው ነገር አለ፤ኤንጅኦ ሲያሰለጥን እንደምታውቀው ብር እየሰጠ ነው የሚያሰለጥን አሁን ተማሪ ሰልጥን ስትለው ብር አለው ወይ፣ አበል አለው ወይ ይልሃል፡፡ ስታፍ ቶታሊ ሰልጥን ስለው አበል አለው ወይ ይልሃል፡፡ እና አሁን እንደነገርሁህ ፒር ኢዱኬሽን ዘንዘልማ ሜዲስንና ይባቭ ካሰለጥን በሁዋላ ቢዝነስና ኢኮኖሚክስ ኮሌጅ አበል ከሌለው አንሰለጥንም አሉን፡፡ ይህ ብቻ አይደለም፤ አድንዛዥ ዕፅ ተጠቂዎችን እንሰጥ ነበር ስልጠና ከኤፍኤማካ ኢትዮጵያ መድሃኒትና ምግብ ቁጥጥር ባለስልጣን አብረው ሁነን ይሰጣቸዋል ለሸሀይ መቶ በር፡፡ አሁን ዩኒቨርሲቲው ለሸሀይ ቡና እንኩዋን በጀት ስለሌለው ያለአበል አንሰለጥንም አሉ፡፡ ሰራዎችን ለማስኬድ ብቻ ሳይሆን ሰልጣኙ ክፍያ… ይሄ ደግሞ አገር የትም ብትደርስ የማያዋጣ ነገር ነው፡፡ ለሰልጣኝ እራሱ ሊጠቀም እንዴት ብለህ ነው አበል እየከፈልህ የምታሰለጥነው፡፡ እና እነዚህ ተግዳሮቶች አሉ፡፡

1. ምናልባት ክፍያ የሚጠይቁ ስራ የምሰራበት ሰዓት ነው ብለው ይሆን ማለትም ስልጠናው ቦታ ሲቀመጡ ማግኘት ያለባቸውን ገንዘብ እያጡ

ኖ… እንደሱ አይደለም፡፡ ሰራተኛው እኮ በስራ ሰዓት ቢሮ መጥቶ ቲንሽ ሰዓት ስልጠና ውሰድ ስትለው ቢሮ መቀመጡን ይወዳል ያለ አበል ከሚሰለጥን፡፡ ለምሳሌ ኤች አይ ቪ ኤዲስ ሜንስትሪሚንግ ስልጠና ተብሎ ሆቴል ላይ ምግብ ሁሉ ይዘን ስታፉ ለስልጠና ይመጣሉ ስንል አልመጡም፤ የግድ አበል እንዲኖረው ሌላ አገር መውሰድ አለብህ፡፡ እና አሁን ሌላ ቦታ ለመስጠት እየተሞከረ ነው፡፡ምን ታደርጋለህ፡፡ ትልቁ ተግዳት እንዲያውም ሰልጣኙ ነው፡፡ እገዛ የሚያስፈልገው አካል አግዘኸውም ብር እየፈለገ ስለሆነ ይህ..ይህ ደግሞ ከራሱ የመጣም አይደለም፤ እነዚያ ያስለመዱት ነው፡፡

1. አሁን የእነሱ (እነዚያ የተባሉት ኤን ጅኦች) ተግባራ ምን ላይ ነው

አሁን የሉም…አንድም የሉም! እከሌ የምትለው የለም፡፡ ፌደራል ራሱ ሃብኮ የለም ፈርሱዋል፡፡፡

1. ምንድን ነው ምክንያቱ

ከት/ት ሚኒስቴርም… ድሮ እቅድ ከት/ት ሚኒስቴር ይመጣ ነበር፤ አሁን ግን የለም፡፡ ማንም ከበላይ አካል ምን እየሰራችሁ ነው የሚል የለም፤ ሪፖርት የሚጠይቅም የለም፡፡ ኤዲስን የተመለከተ የአማራ ክልል ዩኒቨርሲቲዎች ፎረም ነበረን፡፡ ከ2012 ዓ.ም ጀምሮ ግን የለም፡፡ እንደ አዲስ ለማነሳሳት በየዩኒቨርሲቲው ስንደውል ኦፊሱ የለም ኢፊሱ ታጥፉዋል፡፡ ሃብኮ የራሱን ስራ ወርዶ ማየት አለበት፡፡ አሁን ሶስት አመቴ ነው፤ ማንም የጠየቀኝ የለም፡፡

1. ስለዚህ በአንቺ አተያይ ይህ የሆነ ለምንድን ነው ከምን አንጻር ይመስልሻል ትኩረት ያልሰጡት

በሽታው ቀንሱዋል እንዳይባል ብዙ ሺህ ሰው ከቫይረሱ ጋር አለ፡፡ አሁን ደግሞ እያሳለፍን ያለበት ሁኔታ ለበሽታው መስፋፋት የሚያበረታታ ነው… እኔ አስታውሳለሁ ዘመዶቻችንን ያጣን መቼ ነው… ልጅ ሆኘ ኤች አይ ቪን የሰማነው 1983 ዓ.ም ጀምሮ ነበር፡፡ ከ86 እስከ 93 ዓ.ም ብዙ ሰዎችን ነበር ያጣነው፡፡ ያ የሆነበት ምክንያት ጦርነት ስለነበር ነው፡፡ አሁንስ ደግሞ…አሁንስ መነኩሴ ሳትቀር…ምን የተደረገበት ጊዜ ነው ምን… ምን የተደረገበት ጊዜ ነው…እና አሁን ቫይረሱ ይቀንሳል (ጥያቄ ምልክት)… ኢቭን ኢፍ… ምርምር መሰራት አልነበረበትም… ከ2013 ወዲህ እንደ ክልል ስናየው አሰቃቂ ነው የሚሆን፡፡ ቀንሱዋል የሚል አመለካከት የለኝም፡፡ ነገር ግን የስራ ባህላችን ጠያቂና ተጠያቂነት ስለሌለው በየ ኦፊሱ እናት መስሪያ ቤቱ ነግሌክት አድርጎታል፡፡ እናት መስሪያ ቤቱ አንድን ነገር ካላገዘ ካልጠየቀ፣ ካላገዘ፣ ካልተካከለ ማን ለማን ብሎ ይሰራል ሁሉም የሚጠየቅበትን ነው የሚሰራ..የት ደረሰ የሚል አካል ያስፈልጋል፡፡ እናት መስሪ ቤቱ ጀስት ተኝቱዋል ነው የምለው፡፡

1. የኤች አይ ቪ አገልግሎቱን ስትሰጡ ተጠቃሚ ያልሆኑ ማህረሰብ ክፍሎችን ለምሳሌ ድሃ፣ ያልተማረ፣ ገጠር በሚኖሩ የማህበረሰብ ክፍሎችን ለመድረስ የምትከተሉት የአሰራር ስልት አለ

እኩል ተደራሽ ታደርላችሁ ላልኸኝ እንደ ዩኒበርሲቲ እኩል ተደራሽ እያደረግን ነው ብየ አምናለሁ፡፡ ለምሳሌ ወላጅ የሌለውን ልጅ ግቢ ምግብ እዲበላ እየተደረገ ነው፡፡ ምርምርም እየሰራን ነው፡፡ ነገር ግን ጡርነቱ የነበረበትን ቦታዎች ቢጠየቁ ጤና ቢሮውም ተደራሽ ማድረግ አይችልም፡፡

1. ፍትሓዊነትን ያማከለ አሰራር አላችሁን ለምሳሌ ተጎጂ ናቸው የሚባሉ የማህበረሰብ ክፍልን ስትራቲጂከና ታክቲካል አካሄድ እሳቤው ካለ ብትነግሪኝ

ኢኩቲን እንደኛ ኦፊስ የምናየው ሴትና ወንድ፣ ከከተማ አንጻር ከገጠር አንጻር፣ አካል ጉዳተኞች ነው የምናየው፡፡ ከሴትና ወንድ አንጻር ሴቶች ላይ ነው የምንሰራ፡፡ ለአካል ጉዳተኞች እራሱን የቻለ ስልጠና አለን፡፡ ለምሳሌ ቅደም ያልጠቀስሁልህ ቡና ጠጡ መርሃ ግብር አለን እና እዚያ ጋር ስልጠና፣ ብሮሸር መስጠት፣ መወያየት እንዲሁም በይበልጥ ሴቶችን ለማግኘት በየሴቶች ዶርም ቡና ጠጡ መርሃ ግብር ይዘጋጃል፡፡ እና እደ እኛ ኦፊስ የተማረ ያልተማረ ብለን አናየውም ምክንያቱም የተማረ ስለሆነ፡፡ ምናልባት ግን ጤና ሙያተኛ ስለሆንሁና በተለያየ የአገሪቱ ክፍል ስለሰራሁ ኢኩቲን በተመለከተ አስተያየት መስጠት እችላለሁ፡፡ ማበረሰቡ ውስጥም ችግር አለ፡፡ እና አንቀሳቃሽ ያስፈልገዋል፡፡ ኮንቲኒውስ ክትትልና ኢቫልዌሽን ያስፈልገዋል፡፡

1. ተጨማሪ ተግዳሮት ካለ

እያንዳንዱ ተዉዋም ኦፊስ አዘጋጅቶ የሰው ሃይል መድቦ ኮንቲኒዩስ ኢቫልዌሽን ያስፈልጋል፡፡ የት ደረሰ መባል አለበት፡፡

1. ፍትሐዊ አሰራርን ተቁዋማዊ ስለማድረግ የሚሉት ነገር ካለ

ኦፊስ ከፍተን አስቀምጠናል፣ በየግቢው ሳይቀር አለን፡፡ አንዳንድ ግቢዎች ሴቶች ወጣቶችና ኤች አይ ቪ ብለው ቀላቅለዋቸዋል፡፡ እንደ አጠቃላይ ግን ለበሽታው እየተሰጠ ያለው ትኩረት አጅግ አናሳ ከመሆኑ የተነሳ እንደ በፊቱ ገጠር ማህረሰቡንም ግንዘቤ ፈጠራ ስራዎች እየተሰሩ እንዳልሆነ አምናለሁ፡፡ ለምሳሌ ድሮ በየቤተክርስቲያን ሁሉ ይህን ቫይረስ ስለመከላከል ትምህርት ይሰጥ ነበር፡፡ አሁን ግን ይሄ የለም፡፡ ይሄ ነገር አንቀሳቃሽ ያስፈልገዋል፡፡ ኢቫልዌሽን ያስፈልገዋል፤ ኮንቲኒውስ የሆነ ፎሎውአፕ ያስፈልገዋል፡፡ እና እንደ አጠቃላይ እንደ አገር ፍትሓዊ ተደራሽነት የለም፡፡

1. አሁንም ለተጨማሪ መረጃ ያክል ለምን ይመስልዎታል የኤች አይቪ ኤዲስ አገልግሎቶች እኩል ተደራሽ ያልሆኑ

እንደ እኔ አሁን አንደኛ ኤች አይቪን ለመከላከልና ለመቆጣጠር የሆነ አካል ብቻ አይደለም፡፡ በየተቅዋማቱ ኦፊስ መኖር አለበት፡፡ እያንዳንዱ ተቁዋም ራሱን የቻለ ኦፊስ መኖር አለበት፡፡ ኢፊሱ ብቻ በቂ አደለም፡፡ በጀት ብሬክ መቀመጥ መቻል አለበት፡፡ ከላይ እስከ ታች ድረስ ማለት ነው፡፡ ሪስፖንሲብል ሰው መኖር አለበት፡፡ ሪስፖንሲብል ሰው ከተቀመጠ በሁዋላ ደግሞ ፎሎፕ የግድ መኖር አለበት፡፡ ኮንቲኒዩስ የሆነ ፎሎአፕ መኖር አለበት፡፡ ኢቫልዌት መደረግ አለበት፡፡ የትገባ የት ደረሰ መባል መቻል አለበት፡፡ ይህን ነው ልል የምችለው፡፡

ሌላው ብዙ ኦፊሶች ኤች አይቪንና የሴቶች ጉዳይን ሚክስ አድረገውታል፡፡ ሚክስ ስለተደረገ በተናጠል አቅዶ ለመስራት ይቸገራሉ፡፡ በአመት አንድ ወይም ሁለት ስራ እንኩዋን አይሰሩም፡፡ ጀንደር በራሱ በዙ ጉዳይ ያለበት ነው፣ ኤች አይቪም እራሱን የቻለ ብዙ ስራ ያለበት ነው፡፡ እንዴት ነው ሁለቱን አንድ ላይ አድገህ ልታስኬደው የምትችል

1. በሁለት ወይም በሶስት ሁኔታዎች ተጎጁ የሆኑ ማህረሰብ ክፍልን የሚያካትት አሰራር አላችሁ (ለምሳሌ ድሃ፣ ያልተማረ፣ ገጠር የሚኖር)

የእኛ አገር እንደምታውቀው ነው፡፡ ብዙ ስራዎች በግለሰቦች ችሮታ እና ደግነት ነው ስራዎች የሚሰሩት፡፡ ግልጽ የሆነ እቅድ፣ ስትራቲጂ ዶክመነት ተነድፎ አስገዳጅ በሆነ መንገድ ሚያሰራ ፖሊሲ የለንም፡፡ ለምሳሌ በእኛ ኢፊስ ስመጣለህ በእራሳችን ጥረት ፕሮጀክት እንጽፋለን፣ መንግስታዊ ያልሆኑ ድርጅቶችን እናናግራለን፡፡ ኢኮኖሚካሊ ድሃ የሆኑ ሴት ተማሪዎችን ኮሚቴ አዋቅረን እንለያለን፤ ለምሳሌ በዚህ አመት ከጦርነት አካባቢ የመጡ ሴት ተማሪዎችን ነበር ትኩረት አድርገን ስንሰራ የነበር ምክንያቱም ኢኮኖሚካሊ የተጎዱ ስላሉ፡፡ ለእንዚህ የገንዘብ ድጋፍ እናደርጋለን ምክንያቱም በገንዘብ እጥረት ምክንያት ያልሆነ ነገር ውስጥ (ተክፎሎዋቸው ሴተኛ አዳሪ ውስጥ እንዳይገቡ) እንዳያደርጉ፡፡ እንደ አጠቃላይ ግን ፍትሃዊ ተደራሽነት የለም፡፡

1. ቀረ የምትይው ነገር ካለ

ኤች አይቪን ከመከላል አንጻር የሁሉም አካል ሃላፊነት ነው፡፡ ከቤተሰብ እስከ ሐይማኖት አባቶች እንዲሁም ሁሉም ተቁዋም ሃላፊነት ነው፡፡

**#15**

1. Tell me about yourself and your work on HIV

We will make them do awareness creation activities mainly on the issue of HIV; That is, we work with schools, communities, and university staff as well as students. Coordinating and providing trainings, to mention detailed tasks, regarding the youth's bad habits (drugs, gambling, khat) through training, mediation, group council or individual council, we work around this. We will activate, support and strengthen anti-addiction and anti-HIV circles. We also encourage students to discuss with each other, discuss their problems. For example, peer pressure is high at university. We conduct peer-to-peer training so that peer pressure does not lead them into a bad situation. We will train at least 40 students in each campus and let them reach the rest of the students. We also provide life skills training. Sister to Sister, we do this for female students in terms of HIV prevention. For example, we distribute brochures about drugs. In terms of control, we were conducting HIV tests in every campus to find people with the virus in their blood. But this year we lack the diagnostic kit and could not continue. Another is that we distribute condoms. We have condom storage boxes. Condoms come from different partners.

We will support the teachers, administrative staff and students to organize and generate income. We also prepare various trainings for psychological strengthen. For example, about food, when I control the disease. We also provide them with legal protection services. We also financially support children who have lost their parents in the community.

1. In addition to what you mentioned above, what work do you do in terms of making the other community accessible?

If you need to pick it up now, since 2013 E.C, since the arrival of Corona, operations have been slowing down. We formed a task force in 2002 E.C from all community members. Next, due to the war, it was not possible to contact the leadership. We have all been affected by the war, the instability, the lack of budget, and not doing well.

1. If there are tasks that are not done according to your plan, please.

There is unfinished business. As you know, health related issues are mostly funded by non-governmental organizations. It can be said that much of HIV work in particular is concentrated in non-governmental organizations. For example, ‘Sista’ has a self-contained manual; If you say ‘Life skills’, it has an independent manual; if you say ‘Peer Education’, it has an independent manual; It all started with NGOs. NGOs were work with us. The government should be able to allocate a budget to continue these. Now it is at the university, it is just a good idea, but there is no top-down budget set aside for HIV-AIDS prevention and control. There must be a budget to sustain each of these services started by NGOs. Not only this, but there is also something that both the salaried employee and the student need to receive. As you know, when an NGO trains, the person who trains is giving money. Staffs totally are not volunteer to take training in the absence of per diem. And as I told you now, after we gave peer to peer education at Zenzelma, Medicine, and Yibaba Campus, students from Business and Economics campus refused taking training without per diem. Not only that; We used to give training to addicted drug addicts and gave them training per diem from FAMACA Ethiopia Drug and Food Control Authority. They said that now the university does not have a budget even for Tea and Coffee, so we cannot train without per diem. Not only to run the routine activities, but also to pay the trainees... this is something that is not important in any country. How can you train the trainee while they asks you per diem? And there are these challenges.

1. Is this due to that they are losing the money that they should be getting when the training takes place?

No… not like that. The employee comes to the office during working hours and when you tell him to take training at few hours, he likes sitting in the office rather than training without a per diem. For example, when we brought all the food to a hotel called HIV/AIDS menstreaming training, they did not come when we said they would come for training. You must take another city or zone to have them per diem. And now we are trying to give training another place. What do you do? The biggest challenge is actually the trainee. You helped the person who needed help because he was looking for money… this did not come from himself; That's what they (NGOs) taught him.

1. Now what is their (the so-called NGOs) task?

Now they don't exist...none! There is no one to say. The federal government HAPCO itself is no longer there. They have been dissolved.

1. What is the reason?

From the Ministry of Education... Plans used to come from the Ministry of Education; But now it doesn't exist. No one from the tope level says what are you doing; No one is asking for a report. We had an Amhara Region Universities Forum on AIDS. But since 2012, it has not existed. When we call every university to initiate a new one, the office is not there, the office has been folded or dissolved. HAPCPO should look at his own work. I work for three years. No one asked me.

1. So, from your point of view, why does this happen, and from what point of view do you think they did not pay attention to it?

There are many thousands of people with the virus, not to mention that the disease has decreased. The situation we are going through now encourages the spread of the disease... I remember when we lost our relatives... We heard about HIV as a child in 1983. We lost many people from 86 to 93 E.C. That was because it was a war. And now... even now, without a monk left... what time has it been done... what time has it been done... and now the virus will decrease?... even if... research should not have been done?... since 2013 E.C, when we see it as a region, it will be horrible.

I don't think they have reduced it. But because our work habit is unaccountable, the mother office has neglected it. If the mother office doesn't help something, if it doesn't ask, if it doesn't help, if it doesn't fix, who works for whom, everyone does what is asked. I say that the mother office is just sleeping.

1. When providing HIV services, is there a strategy you follow to reach non-beneficiary groups such as poor, uneducated, rural communities?

I believe that we are making it equally accessible as a university. For example, a child without parents is being made to eat food in the University. We are also doing HIV testing. However, if you ask how conflict zone get services, the health office will not also be able to access it.

1. Do you have a system that focuses on equitable services provision, for example, if there is a strategic and tactical approach to the society that is said to be a victim, please tell me?

We see equity as our office, women and men, urban and rural, disabled people. We work on women from the point of view of men and women. We have independent training for people with disabilities. For example, we have a coffee drink program, which I didn't mention to you, and there is a coffee drink program organized in each women's dorm to train, give out brochures, discuss, and more importantly meet women. And in our office, we don't see equity-based on education status because we believe all are well educated. But maybe because I am a health professional and have worked in different parts of the country, I can give an opinion about equity. There is also a problem with population. And it needs a stimulant that makes them actively engaged. Continuous monitoring and evaluation is required.

1. If there is an additional challenge you want to mention, please.

Each sector should set up an office and allocate human resources and conduct continuous evaluation. They should ask where our works looks like.

1. If there is anything to say about making the equity process more formal and institutionalized, please.

We have opened offices; we even have them in every campus. Some campuses have mixed and confused women with youth and HIV. In general, however, the attention being given to the disease is very little, so I believe that creative works are not being done in the rural society as before. For example, in the past, every church taught about preventing this virus. But now this is not the case. This item needs initiator, Needs evaluation. It requires continuous follow-up. And there is no fair access as a whole as a country.

1. Just for additional information, why do you think HIV/AIDS services are not equally accessible?

As to me, it's not just only the responsibility of a single office to HIV prevention and control activities. There should be an office in each district. Every institution should have an independent office. Only Health Bureau is not enough. Budget should be able to put brake. Budget should be allocated. That means from top to bottom. There must be someone responsible. After the Responsible Person is assigned, there must be a continues follow-up. There should be continuous follow-up. Must be evaluated. It should be possible to tell where it entered. That's all I can say.

Another is that many offices comined HIV and women's issues. Because it is mixed, it is difficult to plan and work separately. They don't do one or two jobs a year. Gender is an issue on its own, and HIV is an issue of its own. How can you implement the two together?

1. Have you a system that consider two or three disadvantaged groups (eg poor, uneducated, rural)?

As you know in our country, many activities are done by the generosity and kindness of individuals. We do not have a clear plan, a strategy document, and a working policy. For example, when you come to our office, we write a project on our own efforts, and we talk to non-governmental organizations. We establish a committee to identify economically poor female students; For example, this year we focused on female students from war zones because they are economically disadvantaged. We fund this so that they don't end up in something that isn't due to lack of money (paying them to stay out of prostitution). As a whole, there is no fair access.

14. If there is anything left to see, please.

It is everyone's responsibility to prevent HIV. It is the responsibility of everyone from families to religious leaders.

**#16**

1. የኤች አይ ቪ ኤዲስ አገልግሎት ላይ ያለብሽን ኃላፊነት ብትነግሪኝ

በኤች አይ ቪ ፕሮግራም ላይ ፀረ-ኤች አይ ቪ ህክምና ክትትል ባለሙያ ነኝ፡፡ ስለዚህ ያው ሰዎች በትክክል መድኃኒታቸውን እያገኙ ነው ወይ የሚለውን ነገር በመረጃ እንሰበስባለን እንከታተላለን፡፡ ሂደንም በአካል ክሊያንትም ጋር የምናይበት ሁኔታ አለ፡፡

ኤ አር ትን መሰረት አድርጋችሁ ነው ማለት ነውን.. አዎ፡፡

ክትትል በምታደርጉበት ጊዜ ለየት ያለ የምትከተሉት የአሰራር ስልት አላችሁ

ክትትል ስልህ ተመርምረው ቫይረሱ ከተገኘባቸው ሰዎች በቀጥታ ወይም ቢያንስ በአንድ ሳምንት ውስጥ መጀመር አለባቸው እና መድሃኒት እንዲጀምር ማድረግ፤ ከጀመረ በሁዋላ ደግሞ የመድኃኒት ጋር ቁርኝት ማለት መድኃኒቱን በትክክል መውሰድ የታዘዘለትን መድኃኒት በትክክል መውሰድ እንዲችል ማስቻል ነው፡፡ ግብዓቶቹም ያው እኛ ኮሚኒኬት የምናደርጋቸው እኛ ጋር የሚሰሩ ከግብዓት ዳይሬክቶሬት አለው የቢሮ ሌሎችም እንደ መድኃኒት ስርጭት የሚሰሩ ልክ እንደ ኤብሳ አለ ከእነሱም ጋር ኮሚኒኬት እናደርጋለን፡፡ መድኃኒቶች እንዲኖሩ ማስቻል፣ እና ሰዎች ትክክለኛውን ህክምና እንዲያገኙ ማድረግ፣ ከዚያም በፋሲሊቲ ሆስፒታል ወይም ጤና ጣቢያ ደረጃ በዚያ ላይ በዚህ ላይ የሚሰሩ ጤና ሙያተኞች ላይ ክትትልና ድጋፍ ማድረግ ለምሳሌ ስልጠና መስጠት፣ ሮፖርቶችን በየወሩ መሰብሰብ፣ ዲ ኤች ኤስ መረጃንም እያየን ያሉትን ክተቶች በመሙላት እንሰራለን፡፡ ኤ አር ቲ ስለህ ከምርመራ ይጀምራል፤ አንድ ሰው ተመርምሮ በሽታው ከተገኘበት መድኃኒት እንዲጀምር ይደረጋል፡፡ህክምናው ውስጥ ከገባ በሁዋላም ደግሞ መድሃኒቱ ጋር ያለው ቁርኝት ጥሩ መሆን መቻል አለበት፡፡በትክክል በታዘዘለት መጠን እና ፍሪኮንሲዩን ጨምሮ በቀን አንድ ጊዜ ነው ወይስ ሁለት ጊዜ የሚለውንም ጨምሮ በትክክል እንዲወስዱ ማስቻል እና ይህን ከወሰዱ በሁዋላ መድኃኒት በትክክለኝነት ስለመስራቱ ቫይራል ሎድ መጠን መቀነሱን ሁሉ እንከታተላለን፡፡ ምክንያቱም የመጨረሻ ዓላማው ቫይራል ሰፕረሽን ማምጣት ነው፡፡ እንግዲህ አንድ ሰው ቫይረሱ ካለበት መድኃኒቱን እየተከታተለ ይወስዳል፡፡ ቫይራል ሰፕረሽን ካለ ጤናማ ሁኖ መኖር ይችላል ማለት ነው፡፡ ኢቭን ቫይረሱን ወደ ሌላ ሰው የማስተላፍ እድሉ የቀነሰ ነው የሚሆን፡፡

1. የእናንተ ስራ ከቫይረሱ ምርመራ ማድረግ ነው የሚጀምር እና በሚፈለገው ደረጃ ሁሉም ሰው እኩል ምርመራ ያደርጋል ብለሽ ታስቢያለሽ

ምርመራን አስመልክቶ ትኩረት (ታርጌት) አድርገን ነው የምንሰራ፡፡ ተልዕኮ እና አላማ የምንላቸው ነገሮች አሉ፡፡ ሶስቱ 95ቶችን ማሳካት፣ የኤች አይቪ ቫይረስ ሁኔታ እንደ አገር ከአንድ ፐርሰንት በታች ነው፡፡ የእኛ ክልል ግን ከአንድ ፐርሰንት በላይ ነው፡፡ እንደ አገር ከአንድ ፐርሰንት በታች ስለሆነ ወረርሽኝ ነው የሚያስብል ደረጃ ላይ አይደለም ስለተባለ ታርጌት አድርገን ነው የምንሰራ፡፡ ቫይረሱ በደማቸው ሊገኝ ይችላል የተባሉ የማህበረሰብ ክፍል ላይ ትኩረት የመስጠት ሁኔታ አለ፡፡ ለምሳሌ ኪይ ፖፑሌሽን የሚባሉት ሴተኛ አዳሪዎች፣ የረጅም እርቀት አሽከሪካሪዎች፣ ተጋላጭ የሆኑ ሴትና ወንድ ወጣቶች ላይ ትኩረት ይደረጋል፡፡ ሌሎቹ ደግሞ ኢንጀክቴብል ድራግ የሚወስዱ በእርግጥ እነዚህ እኛ አካባቢ ብዙም ባይሆኑም ትኩረት ማድረግ ያስፈልጋል፡፡ እነዚህ ከባሎቻቸው ጋር የተፋቱ፣ ፈተው የሚያገቡ ሰዎች መመርር ስላለባቸው ትኩረት ይደረጋል፡፡ ከእነዚህ በተጨማሪ ደግሞ ወደ ጤና ተቁዋም ታመው የሚመጡትን፣ ምልክት የሚታይባቸውን ለምሳሌ ቲቢ፣ የአባላዘር በሽታ ያለባቸውን ሰዎች ለኤች አይ ቪ ይመረመራሉ፡፡ ህጻናትም ይመረመራሉ፡፡ ኤች አይ ቪ ሊሆን ይችላል የሚያስብል ምልክት ካላቸው ይመረመራሉ ማለት ነው፡፡

1. ስለዚህ እነዚህ የጠቀስሽልኝ የማህረሰብ ክፍል ዋና ትኩረት አቅጣጫ ቢሆኑም እነዚህ የጠቀስሻቸው ሁሉም እኩል የአገልግሎቱ ተጠቃሚ ይሆናሉ

አሁን ዋናው ነገር ምንድን ነው መሰለህ ዲማንድ ክሬሽን ላይ ነው፤ ያውቃሉ ወይ መመርመር እንዳለባቸው የሚለው ነው፡፡ ካወቁ እንደሚመረመሩ ማስቻል በጤና ተቁዋም ከሄዱ አይመለሱም ይመረመራሉ ማለት ነው፡፡ ነገር ግን ወደ ጤና ተቁዋም እንዲሄዱ በማህበረሰቡ የሚሰሩ አጋር አካላት አሉ፡፡ ወደ ጤና ተቁዋም እንዴት ይደርሳሉ የሚለው ያው የግንዛቤ ፈጠራ መስራት ያስፈልጋልና በማህበረሰቡ ውስጥ የሚሰሩ አጋር አካላት ይሰራሉ፡፡ ሌሎችም ከእኛ ጋር የሚሰሩ ዘርፈ ብዙ ምላሽ የሚባል አካል አለ ይህም ዋና ተግባሩ እነዚህ መረጃዎች እንዲደርሱ የማድረግ እና የመከላከል ስራ ላይ የሚያተኩር ነው፡፡ ሌላው ቫይረሱ በደማቸው ያለባቸው ሰዎች ማህበራት አሉ እነሱም መረጃዎችን ለህብረተሰቡ ያደርሳሉ፡፡ ምናልባት የአማራ ማስሚዲያም አንዳንድ ፕሮግራሞች አሉን፤ የቴሌቪዥን ፕሮግራሞች ሮብ ሮብ አሉን፡፡ በዚህ መልኩ መረጃዎች ለማህበረሰቡ መረጃ ይደርሳል ማህበረሰቡም ይመረመራል ማለት ነው፡፡

1. ይህ ሁሉ ተደርጎ ሁሉም የአገልግሎቱ ተጠቃሚ ይሆናል ወይ

ሁሉም ላይሆን ይችላል፡፡

1. ምክንያቱ ምንድን ሊሆን ይችላል

እራሳቸው የአገልግሎቱ ተጠቃሚዎች ፍርሃት ያድርብናል አንመረመርም ይላሉ፡፡ ቫይረሱ ተገኝቶባቸውም መድኃኒት አንጀምርም ማህረሰቡ ምን ይለናል የሚሉም አሉ፡፡

1. ሁለት ጊዜ እና ሶስት ጊዜ ተጎጂ የሆኑ የማህበረሰብ ክፍሎች አሉ፡፡ ለምሳሌ ገጠር የምትኖር፣ ድሃ፣ ያልተማረች ሴት እንደ ምሳሌ እንውሰድና እንዴት ነው አገልግሎቱን የምታደርሱዋቸው

አገልግለቱ ከተማ ላይ ነው የሚያተኩር ምክንያቱም ቫይረሱ ከተማ ላይ ስለሚበዛ ነው፡፡ ስለዚህ ይበልጥ ውጤታማ ለመሆን ከተማ ላይ መስራት ያስፈልጋል፡፡ ምክንያቱም ብዙ የሚገኝበት ላይ ብንሰራ የመካለከሉም ጉዳይ በዛው ልክ ጥሩ ይሆናል፡፡ የገጠር ሰዎች ከተማ ላይ አድረው ሲሄዱ ነው በሽታውን ይዘውት የሚሄዱ ስለዚህ ከተማ ላይ ነው ትኩረት አድርገን የምንሰራ፡፡ ጥናቶችም እንደሚያሳዩት የኤች አይቪ ቫይረስ መጠን ለምሳሌ በአማራ ክልል 1.2 ፐርሰንት ነው፤ ከተሞች ላይ ባህርዳር፣ ደሴ ከተሞች ከተሞች ላይ ሲጠና ግን 4.1 እና ከዚያ በላይ ነው፡፡ይህ የሚያሳየው ከተማ ላይ ከፍተኛ ስርጭት አለ፡፡ የሰውም ማለት እንቅስቃሴ አለ፡፡ ከዚህ ጋር ተያይዞ የኤች አይ ቪ አገልግሎት የሚደረገው ከተማ ላይ ነው፡፡

1. አገልግሎቱን ሰፋ አድርችሁ የማትሰሩበት ምክንያት ምንድን ነው

ገጠር ላይም እኮ ይሰራል፤ በሁሉም ጤና ተቁዋማት ይሰራል፡፡ ትኩረት የሚደረግ ከተማ ላይ ነው ለማለት ነው፡፡

1. አገልግሎቱን ከመጠቀም አንጻር ሁሉም እኩል አይጠቀምም

ሁሉንም ከመድረስ አንጻር የሰለጠነ የሰው ኃይል ችግር አለ፡፡ ኤች አይ ቪ በራሱ ስልጠና ይፈልጋል፡፡ ነገር ግን የሰራተኞች ዝውውር ስለሚደረግ ኤች አይ ቪ ላይ ሲሰሩ የነበሩ ወደ ሌላ ሲዛወሩ ችግር ያጋጥመናል፡፡ ሙያተኞች ስራ ይለቃሉ፡፡ እና አንዳንድ ጊዜ ደግሞ ኮሚትመንት አለመኖር፤ ስልጠናውን ወስደው በአግባቡ ኃላፊነት አለመወጣት ታያለህ፡፡

1. የተፈናቀሉ ሰዎችን ከመድረስ፣ ጦርነት ሰዓት የተቸገራችሁበት ሁኔታ ምን ትያለሽ

ጦርነቱ አስቸጋሪ ሁኔታ ፈጥሮብን ነበር፡፡ መመርመር አልተቻለም፣ መድሃኒት ተደራሽ ማድረግ አልተቻለም ነበር፡፡

1. እንደ አጠቃላይ ግን ለሁሉም ተደራሽ ከማድግ አንጻር የሚያጋጥማችሁ ተግዳሮት አለ

አዎ ተግዳሮት አለ፡፡ ለምሳሌ ግብዓት በምትፈልገው ልክ አይመጣም፤ይቆራረጣል፡፡ መብትልገው ልክ አይመጣም፡፡ ለምሳሌ የመመርመሪያ ኪት ሲያልቅ አለመምጣት፡፡ ኤ አር ቲ የማቆራረጥ እና በልክ ከፌደራል አለመምጣት፡፡ ግብዓት በጣም ይቆራረጣል፡፡ አዲስ አሰራር ሲቀየር ወዲያውኑ ወደ አዲስ አለመግባት እና የመሸጋገር ችግር ሲያጋጥም ነባሩንም አዲሱንም አለመስራት ይከሰታል፡፡ ለምሳሌ አዲስ የኤች አይ ቪ መመርመሪያ አልጎሪዝም ዘንድሮ ተቀይሩዋል፡፡ እኛ ታርገት አድርገን እንስራ እንጂ አማራ ክልል ከአገሪቱ ከአንድ ሶስኛ በላይ የቫይረስ መጠን በቁጥር ቢኖርም ይህን ያማከለ ፍትሐዊ የሐብት ስርጭት የለም፡፡ እንደ አገር መከፋፈል ሲደረግ የእኛ ከፍ ነው ማለት ያለበት ምክንያም ብዙ ቁጥር ያለ ከዚህ ክልል ነው፡፡ ጫናው ከፍተኛ ሁኖ ያንን መሸፈን የማይችል መሰጠት የለበትም፡፡ ይህንን ደግሞ ሊያስካክለው የአመራር ብቃት ነው፤ሌላ አይደለም፡፡ በርደኑ የት ነው ያለ፤ ብዙ ነገር ለማን ነው የሚያስልግ ሚለውን በትክክል ሌይቶ ማሰራጨት ያስፈልጋል፡፡

ተደራሽ ያልሆኑ ለመድረስ ተሸከርካሪ እጥረት ያጋጥመናል፡፡

ሌላው ሁሉ ሙሉ ነው ማለት አይቻልም፡፡ ስልክ ከራስህ ነው የምትደውል፣ በኢንተርኔት ስብሰባ አለ ግን ሁሉን መከታተል አቻልም ከኢንተርኔት ኢንፍራስትራክቸር አንጻር፡፡

ከተወሰኑ መስሪያ ቤቶች ጋር ተቀናጅቶ መስራት ለ፤ ነገር ግን ሚገባውን ያክል አይደለም ውስን ነው፡፡ ፍትሓዊነትን ተቁዋማዊ የማድረግ ጉዳይ ትኩረት የማድረግ ሳይሆን በብዛት ለቫይረሲ ተጠቂ የሆኑትን የማህበረሰብ ክፍል ላይ ነው የምናተኩር፡፡

1. ወደ ፊት ቢስተካከል የምትይው ነገር ካለ

ምናልባት ከተቁዋም እስከ ላይ ድረስ ትኩረት መስጠት ያስፈልጋል፡፡ ብዙ ሰዎ በወቅታዊ ጉዳይ የመወሰድ እና የመፋዘዝ ጉዳይ ይስተዋላል እና ከዚህ በላይ መስራት ያስፈልጋል፡፡ የቁሳቁስ መቆራረጥ ባይኖር ጥሩ ነው፡፡ ሌላው ማህበረሰቡ ለቫይረሱ ትኩረት አልሰጠው እያለ ነው፤ ሌሎች ናቸው እንጂ ኤች አይ ቪ ደግሞ ምንድን ነው የሚባል ሁኔታ ላይ ደርሰናል፡፡ እና ትኩረት ያስፈልጋል፡፡ ኤች አይቪን አገልግሎት ከሌሎች ጋር አቀናጅቶ የመስራት ልምድ ቢዳበር፡፡ ወጣቶች ላይ አዝወትሮ መስራት ያስፈልጋል፡፡ እና መከላከል ላይ ማተኮር ያስፈልጋል፡፡ ከላይ እስከ ታች ያለው አመራር ትኩረት ሊሰጠው ይገባል፡፡ በተለይ የእኛ ክልል ብዙ ነገሮች አልፈዋል፤ ብዙ ነገሮችም አሉ እና ትኩረት አድርገን መስራት አለብን የሚል አስተያየት አለኝ፡፡

**#16**

1. Tell me about your responsibilities in the HIV AIDS service

I am an anti-HIV treatment monitoring specialist at the HIV program. So, we collect data and track whether the same people are actually getting their medication. There is also a situation where we can go and see the client in person.

1. Do you mean based on ART?

Yes.

1. Please tell me if you have a unique method of monitoring?

I mean follow-up, those who tested positive for HIV should begin immediately or at least one week after testing positive for the virus and start medication; Once started, medication adherence means taking the medication correctly to enable the patient to take the prescribed medication correctly. The resources are the same, we communicate with them, there is a directorate of resources that works with us, there are others who work like drug distribution, like EBSA, and we communicate with them. Enabling medicines to be available, and making sure that people get the right treatment, then monitoring and supporting the health professionals working on this at the facility hospital or health center level, such as providing training, collecting reports every month, and filling in the gaps that we are seeing with DHS data. ART begins with an examination of you; If a person is diagnosed and diagnosed with the disease, he will start medication and have good adherence with it. We monitor viral load reductions to ensure that they are taking the correct dosage and frequency, including once or twice a day, and that the medication is working properly after taking it. Because the ultimate goal is to bring about viral suppression. So, if a person has the virus, he will take the medicine while monitoring. If there is viral suppression, it means he can live healthy. Even the chance of transmitting the virus to another person is reduced.

1. You think that your job starts with testing the virus and do you think that everyone are being tested equally at the required level?

We work as a target regarding HIV testing. There are things we call mission and purpose. Achieving three 95s, HIV prevalence is less than one percent as a country. But our range is more than one percent. As a country, it is less than one percent, so we are working as a target because it is not at a level that can be considered an epidemic. There is a trend of paying attention to the section of the society who tested positive for the virus. For example, attention will be paid to key populations, prostitutes, long-distance drivers, and vulnerable young men and women. The others are those who take injectable drugs although these are not in our focus, but attention should be paid to them. Attention will be given to those who are divorced and remarried with their husbands. In addition to these, those who come to the health center sick, with symptoms such as TB, sexually transmitted diseases, are tested for HIV. Children are also examined. If they have symptoms that suggest they may have HIV, they will be tested.

1. Therefore, although these parts of the community are the main focus, do you think all of them are equally or fairly benefited from the service?

Now, you know… the main thing we are doing is demand creation. It is whether they know, or they should be tested for HIV. Allowing that they will be tested if they have a means to go to health institution that they will not leave untested for HIV, they will be tested and they will not return. But there are partners working in the community. It is necessary to create awareness about how to reach a health facilities, and partners working in the community will work. There are others who are working with us called Multisector Response, whose primary function is on HIV prevention activities. Another thing is that there are associations of people who have the virus in their blood, and they provide information to the society. Maybe Amhara mass media has some programs too; We have every Wednesday television program. In this way, information will reach the community and the community will be tested for HIV.

6.Do you think with all these activities, all parts of the community get services fairly?

Maybe not all.

5. What could be the reason?

The users of the service themselves say that we afraid and we cannot be tested for HIV. There are those who say that if the virus is detected, we will not start treatment because what will the society say to us.

1. There are sections of society that are twice and thrice victimized. For example, let's take a rural, poor, uneducated woman as an example, and how do you deliver the service to them?

The service is focused on cities because the virus is more prevalent in cities. Therefore, it is necessary to work on the city to be more effective. Because if we work on where there is more, HIV prevention will be just as good. It is when rural people go to the city that they get infection from those reside in the city, so we are focusing on the city. According to studies, the rate of HIV virus, for example, in the Amhara region is 1.2 percent. In urban areas, Bahardar, Dessie it is 4.1 and above. This shows that there is a high prevalence in urban areas. And there is many human activities in cities. Along with this, HIV services are provided in the city.

1. What is the reason for not expanding the service?

It also works in rural areas. It works in all health conditions despite the focus is on the city.

In terms of using the service, not everyone uses it equally. There is a problem of skilled manpower in terms of reaching all. HIV requires training on its own. But because of the transfer of staff, we face problems when those who were working on HIV are transferred to another. Sometimes, professional’s turnover from their profession is another challenge. And sometimes lack of commitment; You will see that they have received the training but are not carrying out their responsibilities properly.

1. What do you think of the difficult situation during war and reaching displaced population? The war had created a difficult situation for us. No HIV testing, no access to medicine.
2. Just to summarize, is there a challenge that you face in providing equitable services?

Yes, there is a challenge. For example, input doesn't come when you want it, it gets interrupted. It does not come with the proper quantity per your need. For example, not getting when the diagnostic kit runs out. Interrupted provision of ART and which is not per your need from federal. Input is not continuously supplied and is not equitably distributed. For example, a new HIV testing algorithm was changed this year. We should work as a target, but even though the Amhara region has more than one-third of the country's virus rate, there is no fair distribution of resources. When it is divided as a country, ours should be said to be higher because there are a large number of people from this region. It should not be given if the pressure is high and cannot cover that. To achieve this, it is the ability of leadership, nothing else. Where is the burden? A lot of things need to be properly distributed.

When a new system emerges, there is a problem of not entering and transitioning to a new system.

Another we face a lack of vehicles to reach the inaccessible individuals. It cannot be said that everything else is complete. Even I call from my phone using my own credit, there is an internet meeting, but you can't monitor everything due to the internet infrastructure. There is coordinating with specific sectors; But it is not as much as it should be. We are not focusing on the institutionalizing equity, but focusing on the section of society that is mostly affected by the virus.

1. If there is anything you would like to suggest a solution in the future, please.

Perhaps it is necessary to pay attention from the bottom to the top. A lot of people get caught up by current issues and it needs to be done more. It would be nice if there were no supply interruptions. Another is saying that the society did not pay attention to the virus. We have reached a situation where people ignore HIV but fear other chronic diseases. And attention is needed. It is necessary to work in collaboration with other sectors. It is necessary to work regularly on young people. And there is a need to focus on prevention. Top-to-bottom leadership needs attention. Especially our region has gone through many things. There are many things and I think we need to focus and work on them.

# #17

1. በኤች አይ ቪ ጉዳይ ያለሽን ኃላፊነት ብትነግሪኝ

ኤች አይ ቪ ኤዲስ ፎካል ፐርሰን ለአንድ አመት ያክል ነው፡፡ እንደምታየው ሰው ትኩረት የለውም ነው፡፡ በጣም መዘናጋት አለ፡፡ ኤች አይ ቪ ከዚህ በፊት እንደምውቀው ሳይሆን መልኩን ቀይሮ እስከ ታች ክፍል እየተዛመተ ነው፡፡ ስለዚህ ሰራተኛው በሽታውን እንዳይረሳ ብሮሸር መበተንና ስልጠና እንዲሰጥ ማስተባበር፣ በደማቸው ያለባቸውን እንዲረዱ ማስተባበር፣ እናትና አባታቸውን በቫይረሱ ያጡ ልጆችን ያሳድጋል፡፡ ሰራተኞች ጡረታ ከወጡ አንረዳቸውም፡፡ እኛም የምረዳቸው ነበሩ ጡረታ ስለወጡ አሁን አንረዳም፡፡

በዞንና ወረዳ ድረስ በየመነሃሪያው ስለ ኤች አይ ቪ ግንዛቤ ለመፍጠር እንሰራለን፡፡ ማህበራት አሉ፡፡ በየመነሃሪያው ይሰራሉ፡፡ ረጅም እርቀት አሽከርካሪዎች ላይ እንሰራለን፤ ለምሳሌ ወረታ ደረቅ ወደብ ላይ ስራ እየሰራን ነው፡፡ በየተሸከርካሪዎች ስለ ኤች አይ ቪ መከላከልና መቆጣጠርን ያተኮወረ እንለጥፋለን፡፡

1. ይህን አገልግሎት ስትሰጡ አካታች እንዲሆን ምታደርጉት ነገር አለ

አካታችነት የሚባል ነገር አለ፡፡ እሱን ለመስራት እንሞክራለን፡፡

እኩል ተደራሽ ነው ማለት አይቻልም፡፡ ብዙ መስራት ይጠበቅብናል፡፡

ተደራሽ ያልሆነ ማህበረሰብን ለይቶ የማግኘትና የመስራት ሁኔታ እንዴት ነው፡፡ አካቶ ትግበራ አካል ጉዳተኛ፣ ቫይረሱ በደማቸው ያለ፣ እረጅም እርቀት ተሸከርካሪን አተኩረን እንሰራለን፡፡

1. ፍትሃዊ ወይም አካታች የሆነ ስራ ስትሰሩ የሚያጋጥማችሁ ተግዳሮት ብትነግሪኝ

የፋይናንስ ችግር ነው፡፡ ፕሮጀክት ቀርጸን ለአንዳንድ መስሪያ ቤቶች አስገብተን ምላሽ አላገኘንም፡፡ 2 ፐርሰንት የሚባለው በቂ አይደለም፡፡

የማህበረሰቡ እሴት እና አመለካከት ሌላው ተግዳሮት ነው፡፡ ለምሳሌ ስለ ኤች አይቪ ስቲከር ልትለጥፍ ስትወጣ ስራ አጣሽ እንዴ አንቺ ልጅ ልትባል ትችላለህ፡፡ ማለት ድሮ የነበረ በሽታ ስነበረ አሁን አንቺ እዚህ ጋር ምን አለሽ ልትባል ትችላለህ፡፡ ማህረሰቡ ስለ ኤች አይቪ ጉዳይ ብዙም ጀሮ አይሰጥህም፡፡

1. ተጨማሪ ልትነግሪኝ የምትፈልጊው ነገር ካለ

በጀት ቢስተካከል፣ ማህበረሰቡ ላይ የግንዛቤና የአመለካከት ለውጥ እንዲመጣ ቀጣይነት ያለው ስራ ቢሰራ እላለሁ፡፡

**#17**

1. Tell me about your responsibility in the HIV issue

I work as HIV/AIDS Focal Person for one year. As you can see, the people are not paying attention. There is so much distraction. HIV is not like I knew it before; it has changed its form and is spreading to the lower class. Therefore, so that the worker does not forget about the disease, I distribute brochures and coordinate to provide training, coordinate to help those with the disease, raise children who have lost their mother and father to the virus. We don't help employees with HIV/AIDS if they retire. We used to help them too, but now we don't because they have retired. We work to create awareness about HIV in every zones and districts. There are associations. They work in every department. We work on long distance drives; For example, we are working on Woreta Dry Port. We will be posting articles on HIV prevention and control on a weekly basis.

1. When you provide this service, is there something you can do to make it inclusive?

There is such a thing as inclusiveness. We will try to work it out. It cannot be said that it is equally accessible. We have a lot to do.

1. How to identify and work with an underserved community?

Inclusive service… we focus on disabled, virus-infected, long-distance vehicles.

1. Tell me about a challenge you face when doing fair or inclusive work please.

It's a financial problem. We designed a project and submitted it to some offices but did not get a response. 2 percent is not enough.

Community values ​​and attitudes are another challenge. For example, when you go out to post stickers about HIV, they said ‘are you out of a job’. I mean… they believe HIV is an old and forgotten disease so that they told me "What's wrong with you here?, what are you doing here?" Society does not give you ear to hear about HIV.

1. If there is anything else, you want to tell me please.

If the budget is adjusted, I would say that continuous work should be done to bring about a change of awareness and attitude in the society.

12th responsible in the employer and employee affairs authority (28:36 minutes) Abraham

1. If you tell me about your responsibility regarding HIV

I am an expert on HIV and gender issues. Therefore, I have been in this category for more than five years.

**#18**

1. እባክህ ስለ ኤች አይ ቪ ጉዳይ ያለብህን ኃላፊነት እና ስለምትሰሩት ስራ በዝርዝር ንገረኝ

እኔ በጤና ቢሮ ኤች አይ ቪን መከላከልና መቆጣጠር ኬዝ ቲም አስተባባሪ ነኝ፡፡ በዋናነት ኤች አይ ቪ እና የአባላዘር በሽታዎች መከላከልና መቆጣጠርበ ኦፊሰር ነኝ፡፡

እንደ አገር የመከላከልና የህክምና አገልግሎት በሚል ተከፍለዋል፡፡ እኛ በዋናነት ህክምናው ላይ ነው ትኩረት የምናደርገው፡፡ ማለትም አንድ ሰው ቫይረሱ በደሙ ከተገኘበት ጊዜ ጀምሮ አገልግሎት እንዲያገኝ፣ ግብዓት ለጤና ተቋማት እናደርሳለን፡፡ ክፍተቱን እያየን እንሞላለን፡፡

1. ይህን ስራ ስትሰሩ የምትከተሉት የአሰራር ስልት/ዘዴዎች ካለ ብትነግረኝ

የአሰራር ስልቱ ኤች አይ ቪ በጂኦግራፊ፣ በተወሰኑ አካባቢዎች በብዛት ይከሰታል፤ አንዳንድ አካባቢዎች ደግሞ አነስተኛ ጫና አለው፡፡ ስለዚህ ይህንን ያማከለ የአገልግሎት ለመስጠት የተቻለንን እናደርጋለን፡፡ በዋናነት አገልግሎቱን ተደራሽ ለማድረግ እንሰራለን፡፡ ሁሉም ወረዳዎች የኤች አይ ቪ አገልግሎች እንዲኖራቸው እናደርጋለን፡፡ ከዚህ በተጨማሪ ለሁሉም ተደራሽ እንዲሆን እንሰራለን፡፡ በየአካባቢው ወጪ ቆጣቢ በሆነ መልኩ ተደራሽ እንዲሆን እንሰራለን፡፡ ስለዚህ በክልላችን ወደ 1200 የሚሆኑ የመንግስት፣ የግል እና ፕራይቬት ጤና ተቋማት አሉ፡፡ በእነዚህ ሁሉ አገልግሎቱ እንዲኖር ይደረጋል፡፡ በአጠቃላይ ከ1200 በላይ ምርመራውን የሚሰጡ እና ከ400 በላይ ፀረ-ኤች አይ ቪ መድኃኒት የሚሰጡ አሉ፡፡

1. ያልተሰሩ ስራዎች አሉ ብለህ የምታስባቸው ካሉ ብትነግረኝ

ከሚሰሩ ዋና ተግባራት በሶስት ኢላማዎች የተከፈለ ነው፡፡ ማለትም ሶስቱ 95 የሚባሉት ናቸው፡፡ በእያንዳንዱ ኢላማ ላይ ክፍተት አለ፡፡ ህብረተሰቡም እኩል በአግባቡ ተጠቃሚ ያልሆነበት ሁኔታ አለ፡፡ በጤና ተቋማት ላይ የሚሰሩ ስራዎችንም ተግባራዊ ከማድረግ አንጻር የሰው ኃይል ችግር እና ሌሎች ስራዎችም ጫና መኖር በታቀደው ልክ እንዳይሰራ ይሆናል፡፡ ሌላው አንዳንድ የማበረሰብ ክፍል አድሎና መገለልን በመፍራት የአገልግሎቱ ተጠቃሚ አለመሆን እና እድሉ ቢፈጠርላቸውም ፈቃደኛ አለመሆን ነው፡፡ ይህንን ለመቅረፍም ተገልጋዩ እራሱን በእራሱ ወይም በባለሙያ ታግዞ እንዲመረምር ማድረግ ነው፡፡ አሁን ግን እራሱን በእራሱ እንዲመርምርና በፈለገው ቦታ ሂዶ ህክምና እንዲከታተል ይመከራል፡፡ ይህ እንዲሆን ግን ግብዓት መሳካት ያስፈልጋል፡፡ ሌላው ጥንቃቄ የጎደለው ግብረ-ስጋ ግንኙነት በሚጋለጥበት ጊዜ ከ19 ዓመት በታች ያሉት ልጆቹን እንዲያስመረምር ከማድረግ አንጻር ትልቅ ክፍተት አለብን፤ ባለሙያውን ቆጥሮ መስራት ላይ ችግር አለ፡፡ እንዲሁም በተገልጋዩ በኩል ደግሞ ኃላፊነት ወስዶ ተጠቃሚ መሆን ያለባቸው ሰዎች አምጥቶ አለማስመርመር ሁኔታ ነው፡፡ አንዳንድ ተጠቂ ናቸው የሚባሉ የማህበረሰብ ክፍል ለምሳሌ ሴተኛ አዳሪ፣ እረጅም እርቀት አሽከርካሪዎች፣ ለሱስ ተጋላጭ የማህረሰብ ክፍል፣ ቦሎቻቸው የሞተባቸው ሰዎች መካከል ወደ ጤና ተቋም የማይመጡበት ሁኔታ አለ እና ወደ እነርሱ ሂደን አገልግሎቱን ከማድረስ አንጻር ክፍተት አለብን፡፡ በምርመራው በኩል ክፍተት እንዳለ ሁሉ ሁለተኛው 95ን ከማሳከት አንጻርም አልቻልንም፡፡ ይህም በተለያየ ምክንያት መድሃኒት የማይጀምሩ አሉ፡፡ በተመሳሳይ ሶስተኛው 95ንም ማሳካት አልቻልንም፡፡ ከግብዓት አንጻር፣ ባለሙያው ተረጋግቶ አለመስራት፣ መልቀቅና ወደ ሌላ ቦታ መዛወር ችግር ይፈጥራል፡፡

1. ፍትኃዊ አገልግሎት አሰጣጥን በተመለከተ የምትለኝ ነገር ካለ፣ ለምሳሌ ያልተማሩ፣ ዝቅተኛ ገቢ ያላቸው፣ ገጠር የሚኖሩ እኩል ተደራሽ እንዳልሆኑ ይስተዋልል ስለዚህ ጉዳይ ብትነግረኝ

ፍትኃዊነትን በተመለከተ በጾታ እናያለን፣ በቦታ፣ በእድሜ፣ በፖፑሌሽን ካቲጎሪ እናያለን፡፡ እና ሴቶች፣ ህጻናት አገልግሎት ተጠቃሚ እንዳልሆኑ ያሳያል፡፡ በተጨማሪም ከተማ አካባቢ ያሉ ሴቶች አገልግሎት እንደማያገኙ እንረዳለን፤ ይህንንም ቫይረሱ በብዛት ያለበትን አካባቢ ከግምት በማስገባት ነው፤ ለምሳሌ ከተማ ቫይረሱ ይበዛል፡፡ በተለይ ሴተኛ አዳሪዎች ካሉበት አካባቢ ለእነሱ ምቹ የሆኑ ሙያተኛ በማዘጋጀት እና በእኛ ክልል ወደ 32 የሚሆኑ ሁሉንም አገልግሎቶች ያሉበት ጤና ተቋም ለሴተኛ አዳሪዎች ምቹ በሆነ መልኩ አገልግሎት የሚሰጡ አሉ፡፡ በአሁን ሰዓት ከ600 በላይ ሴተኛ አዳሪዎች የአገልግሎቱ ተጠቃሚ ናቸው፡፡ በ32 ክሊኒክ አምስት አባላት ያሉበት ፒር ግሩፕ አለ፤ እነዚህ ወደ ሴተኛ አዳሪዎቹ እየሄዱ የማምጣት ስራ ይሰራሉ፡፡ ሌላው ከማህበረሰቡ በኩል ባል ወይም ሚስት ቫይረሱ ቢገኝባቸው ለአንዳቸው አለመናገር እና በሽታውን ደብቆ መያዝ አገልግሎቱን በሚገባ ልክ መስጠት አያስችልም፡፡

1. ፍትሐዊ አገልግሎት ለመስጠት ተጨማሪ ተግዳሮት የምትነግረኝ ካለ

የግብዓት መቆራረጥ አለ፡፡ የመመርመሪያ ኪት፣ የቫይራል ሎድ ሪኤጀንት፣ ሲዲ4 ካረቲሌጅ እነዚህ በተከታታይ እጥረት የሚከሰትበት ሁኔታ አለ፤ እንደ ችግር እነዚህ የማይቀሩ ናቸው፡፡ ከፖለቲካ ክንፍ በኩል ዓለምአቀፋዊ እና አገራዊ እቅዶች እንዲሰሩ ምንም ችግር የለበትም፡፡ ነገር ግን ይሄ ይሄ ስራ ምን ደረሰ፣ ምን ደረጃ ላይ ነው ብሎ ክትትል የማድረግ ሁኔታ ቲንሽ ችግር አለ፡፡ ከዚህ በፊት የኤች አይ ቪ ምክር ቤት የሚባል ነገር ነበር አሁን ደግሞ ሃብኮ የሚባለው ድርጅትም ፈርሷል፡፡ ስለዚህ ወደ ጤና ተቋም ቢወርድም እሱን ኦን ለማድረግ ጥረት ላይ ነው ያለነው፡፡ በየቀበሌው ምክርቤት ተቋቁሞ የኤች አይቪን ጉዳይ በደንብ የሚገመግም ነበር እርሱ አሁን የለም፡፡ በዚህ ምክንያት በሁሉም ዘርፍ ይደረግ የነበረው እንቅስቃሴ ቲንሽ ክፍተት አለ፡፡ በዚህም የተነሳ አፈጻጸሙ ምን ይመስላል የሚለውን ማወቅም አንችልም፡፡ እሱ ላይ ክፍተት አለ፡፡ ሌላው የፋይናንስ ሁኔታ ነው፡፡ ሲዲሲ ነው በዋናነት በጀት የሚመድብ፡፡ እንደ አጠቃላይ በቂ በጀት አለ ማለት አይቻልም፡፡ አሁን ደግሞ የአገሪቶ ሰሞነኛ ሁኔታ በተለይ ባለፈው ሁለት ዓመት ክልሉ በጦርነት የተጎዳ ነው፡፡ ወደ ስምንት ዘጠኝ የሚሆኑ ዞኖች ቀጥታ በጦርነቱ ተጎጂ የነበሩ ሲሆኑ ሌላውም በተዘዋዋሪ ተጎጂ ነው፡፡ ስለዚህ በዚያ ቦታ ያሉት ጤና ተቋማት መዘረፍ፣ መፍረስ፣ ሙያተኞች መገደልና መበተን አገልግሎቱን መስጠት አልቻልንም ነበር፡፡ አጠቃላይ የኤች አይ ቪ ፕሮግራም የፈረሰበት ሁኔታ ነበር፡፡ ኮምፒተሮች መዘረፍ፣ ቁሳቆሶች መፍረስና መዘረፍ መረጃውና ሲስተሙ መስራት እንዳይችል ተደርጓል፡፡ ብዙ ሰዎች መድኃኒት አቁመው ነበር፡፡

1. ሁለትና ሶስት ጊዜ ተጎጂ የሆኑ የማህበረሰብ ክፍልን ተደራሽ ከማድረግ አንጻር የምትለኝ ነገር ካለ (በእሳቤ፣ በፖሊሲ ደረጃ ካለ)

ዘርፈ ብዙ ችግር ያለባቸው ሰዎች እኩል ተጠቃሚ ናቸው ማለት አይቻልም፡፡ እነዚህ ሰዎች ወደ አገልግሎት እንዴት ይምጡ እሚለው ላይ መስራት ያስፈልጋል፡፡ ጤና ኤክስቴንሽን መጠቀም እና በቅርብ እርቀት አገልግሎቱ አለ ስለዚህ እንዴት ይምጡ እሚለው ላይ በማህበረሰቡ በኩል የእውቀት ክፍተት አለ፡፡ ዘርፈ ብዙ ችግር አለባቸው የሚባሉ በዋናነት ባለቤቶቻቸው የሞቱባቸው ሰዎች ናቸው፡፡ እነሱ ላይ እንሰራለን ምክንያቱም በቫይረሱ ተጠቂ ናቸው፡፡ ሴተኛ አዳሪዎችንም ይጨምራል፡፡

1. ወደ ፊት ቢስተካከሉና ቢሰሩ የምትላቸው ነገሮች ካሉህ

ኤች አይ ቪ በደማቸው ኑሮ መድኃኒት አንጀምርም የሚሉ አሉ፡፡ ይህ ጉዳይ በምን አይነት መልኩ መፈታት አለበት የሚለው የፖሊሲም ጉዳይ ነው፡፡ባሎች ፖሲቲቭ ሁነው ለሚስቶቻቸው የማይገሩ እና የሚስትና ልጆቻቸውን መመርመር የማይፈቅዱ ሰዎች ስላሉ በፖሊሲ ደረጃ አቅጣጫ ሊቀመጥት የሚገባ መሆን አለበት፤ ምክንያቱም እንደ መበት ነው የሚያዩት፡፡ ግብዓት ያለመቆራረጥ መቅረብ አለበት፡፡ ኮንዶም ስርጭት በፕሮግራም የማይመጣበት ሁኔታ ነው ያለ እና እሱ ጉዳይ ቢስተካከል፡፡ እንደ አገር ይህ ስለቆመ መስተካከል አለበት፡፡ በተለይ ጦርነት ያለበት አካባቢዎች ኮንዶም ስርጭት የግድ ያስፈልጋል፡፡ ወታደሮች በብዛት የሚንቀሳቀሱበት ነው፤ ለማህበረሰቡ ተከታታይ የበሽታውን መከላከል ግንዛቤ ቢሰራ፡፡ በማህበረሰቡ በኩል ደግሞ ኤች አይ ቪ አሁን የለም የሚል ዝንባሌ ስላለ እርሱ ላይ የግንዛቤ ፈጠራ እንደ አዲስ ቢነቃቃ ጥሩ ነው፡፡ በዋናነት አዳዲስ አሰራሮች ሲመጡ በተሎ አውርዶ ለሙያተኛው እውቅና በመስጠት ስራ መስራት ላይ የበጀት ችግር ስላለ እርሱ ጉዳይ መስተካከል አለበት፡፡

**#18**

1. Please tell me in detail about your responsibilities and activities related to HIV.

I am the HIV Prevention and Control Case Team Coordinator at the Health Bureau. I am mainly an HIV and STD prevention and control officer.

Services are divided into prevention and medical services. We mainly focus on the treatment. That is, we provide resources to health facilities so that a person can get services from the time the virus is found in his blood. We fill the gap as we see it.

2. Please tell me if there are any working strategies/methods you follow while doing this work

HIV is more prevalent geographically, in certain areas; Some areas have less pressure. So, we do our best to provide this focused service. We work to make the service accessible to each and all districts. We will make all districts have HIV services. In addition, we work to make it accessible to all. We work to make it accessible in a cost-effective manner in every area. Therefore, there are about 1200 public, private and private health institutions in our region. Through all of these, the service will be made available. In total, there are more than 1200 that offer the test and more than 400 that offer anti-HIV drugs.

3. Please tell me if there are any tasks that you think have not been done

It is divided into three main objectives. That is, the three 95. There is a gap on each target. There is a situation where the society is not benefiting equally. From the point of view of implementation of work in health facilities, there is a problem of manpower and workloads. Another contributing factor community are not using the service due to fear of being discriminated against and being unwilling even when given the opportunity. To overcome this, the clients will test himself or herself for HIV with the help of a professional or independently. But now, it is recommended that they test themself and go and seek treatment wherever they want. But for this to happen, resources are needed. Another gap is in getting children under the age of 19 to be screened when they exposed to unprotected sex. There is a problem with allocating professionals. Also, on the user's side, it is a situation where the people who should take responsibility and be the users bring it and do not be tested for HIV. There is a situation among some vulnerable sections of society, such as prostitutes, long-distance drivers, drug-users groups, and widows, who do not come to health facilities, and we have a gap in terms of going to them and delivering the services. As there is a gap on the HIV testing, we were unable to finish the second 95. There are those who do not start medication for various reasons. Similarly, we could not achieve the third 95. From a resource problems, professional instability, turnover, and relocation can be problematic.

4. If you have something to tell me about equitable service delivery, please, for example, it will be noticed that uneducated, low-income, rural people are not equally accessible.

When it comes to equity, we see it by gender, by location, by age, by population category. And it shows that women, children are not benefiting from services. We also understand that women in urban areas do not receive services; This is done considering the area where the virus is most prevalent. For example, the virus spreads in cities. Especially in the area where there are prostitutes, by preparing professionals who are convenient for them, and there are about 32 health facilities in our region that provide services to prostitutes in a convenient way. Currently, more than 600 prostitutes are using the service. At 32 clinics there is a peer group of five members for this group of population; These go to the prostitutes and do bring them to health institutions. On the other hand, if a husband or wife is diagnosed with the virus from the community, not telling one of them and keeping the disease hidden, so that we cannot provide the service properly.

5. If there is an additional challenge to provide equitable service, please tell me

There is a resource shortage. Diagnostic kits, viral load reagents, CD4 cartilage, these are chronically in short supply; As a problem these are inevitable. There is no problem in making global and national plans from the political wing. But there is a small problem of monitoring what has happened to this work, what is its status. Before there was something called the HIV Council and now the organization called HABCO has also been dissolved. So, we are trying to turn it on, even if it goes down to the health facility. Every kebele council was established to evaluate the issue of HIV which is no longer there. As a result, there is a small gap in the activities that were done in all sectors. As a result, we cannot know what the performance will be like. There is a gap in it. Another is the financial situation. It is the CDC that primarily allocates the budget. It cannot be said that there is sufficient budget as a whole. And now, the current issues in the country, especially in the last two years, the region has been affected by war. About eight to nine zones were directly affected by the war and another was indirectly affected. Therefore, the health facilities in that place were looted, destroyed, and professionals were killed and scattered, so we could not provide the services. It was a situation where the entire HIV program had collapsed.

6. If there is anything you can tell me in terms of making access to the twice or thrice disadvantaged community, please

People with multiple problems cannot be said to benefit equally. It is necessary to work on how to bring these people to service. There is a knowledge gap on the part of the community about how to use Health Extension and how to access the service at a distance. Those who are said to have multiple problems are mainly those whose husbands have died. We work on them because they are infected. It also includes prostitutes.

7. If you have somethings to say that should be done in the future, please

There are those who say that we will not start treatment for HIV in their blood. How this issue should be resolved is a matter of policy. There are people who do not tell their wives to be positive and do not allow their children to be tested, so it should be put in the direction of policy. Because they see it as a right. Supplies must be provided without interruption. There is a situation where condom distribution is stopped, and I would say this should be solved. As a country this has stopped and needs to be fixed. Condom distribution is especially necessary in war-torn areas. It is where soldiers are most active for sexual practice. I would say awareness of continuous disease prevention should be available to the community. On the part of the society, there is a tendency that HIV does not exist anymore, so it would be good if the creation of awareness about it is invigorated as anew agenda. When new procedures are introduced, there is a budget problem in doing activities, like providing training to the professional that should be managed properly.

**#19**

1. ስለ ኤች አይቪ ጉዳይ የስራ ድርሻህ

አሁን ያለሁበት የአማራ ክልል ኤች አይ ቪ በደማቸው ያለባቸው ማህበራት ጥምረት ይባላል፡፡ በክልሉም ሆነ በሀገር አቀፍ ደረጃ ኤች አይ ቢን ከመከላከልና መቆጣጠር አኩዋያ እንዲሁም የህክምና አገልግሎትን በተመለከተ የራሱን አስተዋጽኦ እያደረገ ይገኛል፡፡ በዚህ ውስጥ የኔ ድርሻ ፕሮግራም ዳይሬክተር ነኝ፡፡ የስራ ድርሻዎች አመታዊ እቅዶችን ማውጣት፣ ዳይሬክት የማድረግ፣ ኦርጋናይዝ የማድረግ ድርሻ ነው ያለኝ፡፡ ስራዎቻችን በአብዛኛው እንደ አገር ያለውን እቅድ በክልል ደረጃ ደግሞ ከስተማይዝድ ተደርጎ የሚሰራውን በተለይ ደግሞ ዩኤስ ኤድ ያስቀመጣቸውን እነዚህ አምቢሺየስ የሚባሉትን ሶስቱ ዘጠና አምስቶች ለማሳካት እና በ2030 ኤዲስን ስጋት ለማጥፋት የመከላከል ስራዎች ይሰራሉ፡፡ ለምሳሌ ምርመራ በስፋት ይሰራል፡፡ ከዚያ ተጨማሪ ደግሞ ደንበኞቻችን ኦን ዘ ኮንቲነም ኦፍ ኬር ወይንም ደግሞ በህክምና ውስጥ እንዲቆዩ ከማድረግ እቅዶቻን በዚያ የተዋቀሩ ናቸው፡፡ እንደ ጥምረት ወደ ሶስት የሚጠጉ ፕሮጀክቶች አሉት ወደ 221 የሚሆኑ ሰራተኞች አሉ፡፡ እና እነዚህ ሰራተኞች አቀናጅተን እየሰራን ነው ያለን፡፡

1. እነዚህን አግልግሎት በምትሰጡበት ጊዜ የምትከተሉት ስትራቴጂ ምን ይመስላል

የእኛ የመጀመሪያው እንደ ጥምረት ጅፓ ፕሪንሲፕል የሚባል አለ፡፡ ጅፓ ፕሪንሲፕል ማለት ግረተር ኢንቮለቭምተ ኦፍ ፒፑል ዊዝ ኤች አይቪ ኤዲስ ማለት ነው፡፡ ምን ማለት ነው በአማርኛው ማንኛውም ኤች አይ ቪ ፕሮግራም ፕላኒንግ፣ ኢምፕሊመንቴሽን ሞኒተሪንግ፣ ኢቫልዌሽ ጀምሮ የራሳቸው ኤች አይቪ በደማቸው ያሉ ሰዎች ኢንቮልቭመንት እንዲኖር ይፈለጋል፡፡

1. ያልተሰሩ ስራዎች አሉ ብለህ የምታስብ ከሆነ ብታብራራልኝ

ስትራቴጅክ እቅዱ የሚያስቀምጣቸው ወደ ስድስት የሚጠጉ ስትራቴጅክ ጎሎች አሉት፡፡ ግን ከዚህ በፊት የነበረው የሪሶርስ ምደባ የለም አሁን ጊዜ ምናልባት ፕሪቫለንሱ ቀነሰ ተብሎ ይሆናል፡፡ በሌላ ተጉዋዳኝ ግን እንደ አማራ ክልል ስናይ የአማራ ክልል ኤች አይ ቪ መጠን ከናሽናል ፕሪቫለንስ ሬት ይበልጣል፡፡ እንደዛም ሁኖ ግን ፕሪቨንሽን ፓኬጆች በአብዛኛው እየተሰሩ አይደለም፡፡ በእርግጥ እኛ ፕሪቨንሽን እንሰራለን፡፡ ነገር ግን ታርጌትድ ሁነን ነው፡፡ ለምሳሌ ኪይ ኤንድ ፕራዮር ፖፑሌሽን የሚባሉ አሉ ለምሳሌ ድራይቨር፣ ኢንጀክተብል የሚጠቀሙ፣ ወጣት ሴቶች፣ መለዮ ለባሾች፣ ሴተኛ አዳሪዎች ለእነሱ ብቻ ነው እየተሰራ ያለው፡፡ ያም ለቴስቲንግ ዲማንድ እንዲያደርግ፣ ኢንዴክስ ኬዞችንም ስንፈልግ ተጋላጭ የሆኑትን ሰዎች የመፈለግ እንጂ ጀኔራል ፖፑሌሽን ተብሎ በአገር አቀፍ ደረጃ ቀድሞ በነበረው ደረጃ የህትመትም የሚዲያም የተለያዩ ፕሪቨንሽን ስራዎች እየተሰሩ አይደለም፡፡ እና ከህዝብ አንዳንድ ስንሰማ በጥናት ያልተደገፈ ቢሆንም ከኤች አይ ቪ ይልቅ ክሮኒክ በሽታዎችን የመፍራት ስኩዋር፣ ደም ግፊት እንጂ ኤች አይ ቪ በቃ መድሃኒት እየዋጡ መኖር ይቻላል የሚለውን ግንዛቤ ስለያዘ ቀለል አድርጎ ማየት አለ፡፡ እና ትምህርት ያስፈልጋል አሁንም፡፡ እንደዛም ሁኖ ምርመራው ላይም ስንመጣ ደግሞ ሰው ሁሉ የራሱን ስታተስ ለማወቅ እየተመረመረ አይደለም፡፡ያን ለማድረግም ብዙ በጀት ይፈልጋል፤ ብዙ የካውንስሊንግ ስራዎችን ይፈልጋል እና ስልጠናዎች የመረጃ ፓኬጆች እነዚህ መድረስ በሚገባን ልክ እየደረስን ነው ብየ አላስብም፡፡

1. የኤች አይ ቪ አገልግሎት ፍትሃዊ ተደራሽትን እንዴት ነው

የኤች አይ ቪ ፕሮግራሞች በመንግስት ደረጃ እንኩዋን እየተሰራ ያለው በፊት አንደ ቢሮ ነበር፤ መንግስት እውቅና ሰጦት ኤች አይ ቪ ሴክሬታሪ ተብሎ በክልልም በዞንም በአንድ ቢሮ ነበር ሲሰራ የነበረው፡፡ አሁን ግን የሪሶርስ ተግዳሮትም ሲመጣ የመንግስት ትኩረትም ሲያንስ አሁን አንድ ፕሮሰስ ሁኑዋል ጤና ቢሮ ውስጥ፤ ማለት አንድ የስራ ሂደት ማለት ነው፡፡ ሁኖ… በተወሰኑ ሰዎች በተወሰነ በጀት (ዊዝ ሊሚትድ ፐርሶኔል፣ ዊዝ ሊሚትድ በጀት) ነው እየተሰራ ያለው፡፡ ኢንተግሬትድ ተደርጎ እንኩዋን ያን ያክል ትኩረት ተሰጥቶት እየተሰራ አይደለም፡፡ እና በአለው የበጀት እጥረት ምክንያት አሁን አሁን አለም አቀፍ አሰራሮች የመጡት ታርጌትድ የሆነ ፎከስ ሁነህ እንድትሰራ ነው፡፡ ይህ ደግሞ ምን ያሳያል ፍትሃዊ ያልሆነ የተወሰነ ሴግመንት ኦፍ ዘ ፖፑሌሽን ላይ እንድታተኩር የሚያደርግ ነው፡፡ እና እሱ እሱ ላይ ክፍተት አለ ብየ አስባለሁ፡፡

1. ምናልባት ፍትሓዊ እንዳይኑ ከሚያደርጉ ምክንያቶች ልትጠቅስልኝ ትችላለህ ይሆን..

እንግዲህ ትኩረት ማነስ በማህረሰቡም በመንግስትም ችግር አለ፡፡ ማህበረሰቡ ፍላጎት የለውም፡፡ ሪሶርስ አቅርቦትም ችግር አለ፡፡ ፍላጎት ቢኖርም ደግሞ ለምሳሌ፡ ሰዎች ሲጋቡ ቅድመ ምርመራ ያደርጋሉ ነገር ግን ኪት የለም፡፡ ዋናው መሰረታዊው ሪሶርስ ነው፡፡ በአንድ በኩል ያን ለማድረግ የሚፈል ሰዎች እያገኙ አይደሉም፡፡ አንዳንዶች ደግሞ ወቅታዊ የሆነ የትምህርትና ግንዛቤያቸው እየዳበረ ባለመምጣቱ ዝም ብለው ይጋባሉ፡፡ ስለዚህ ዋና ምክንያት ቅድም እንዳልሁህ የሰው ሃይል ማነስ፣ የበጀት ችግር እና በተለይ ደግሞ የአዌርነስ ጉዳዮች ፍትሓዊ እንዳይሆን አድርገውታል፡፡

1. በፖሊሲና ስትራቴጂ ደረጃ ከገቢ ምንጭ፣ ትም/ት ደረጃ፣ ከተማና ገጠር ምክንያት የሚታዩ ኢፍትሓዊነትን ለመቅረፍ የምትሰሩዋቸው ስራዎች ካሉ ብታብራራልኝ

አካታችነትን እናበረታታለን፡፡ አራት ዋና መርሆች አሉን አካታችነትን እና ጾታዊ እኩልነትን ጨምሮ ነገር ግን በዚያ ደረጃ ወደታች ሲወርድ በአፈጻጸም ደረጃ እየተፈጸመ አይደለም፡፡ በተለይ እንደ እኛ ሴክተር ተደራሽ የምናደርገው መንግስታዊ ባልሆኑ ድርጅቶች እገዛ ስለሆነ የዶነርን ፍላጎት መሰረት ያደረገ ነው፡፡ ስለዚህ ይህ ራሱ ፍትሓዊነትን እንዳይመጣ ያደርጋል፡፡ በመንግስት ደረጃ ምናልባት የፖሊሲን ሁኔታ የማይኖራቸው ሊኖሩ ይችላሉ፡፡ ነገር ግን ያሉትን መመሪያዎችና ፖሊሲዎች ደግሞ ዘይ አር ኖት ኢምፕልመንትድ ኢፌክቲቪሊ፡፡ እናም ደግሞ የተጠናከር ታይለርድ የሆነ ለምሳሌ ሴተኛ አዳዎች፣ ለሴቶች፣ ተጋላጭ ለሆኑ የማህረሰብ ክፍል ላይ ጠንካራ ስራ እየተሰራ አይመስለኝም፡፡

1. ይህ ክፍተት እንዲፈጠር ከሚያደርጉ ነገሮች ምን ሊሆኑ ይችላሉ

የኮሚትመንት ችግር አንዱ ሊሆን ይችላል፡፡ ነገር ግን ደግሞ የህብረተሰቡም ነግሊጀንስ አለ፡፡ ለህክምና አገልግሎት ፍላጎት አለመኖር፡፡

1. በእናንተ በኩል የምትሰጡት አገልግሎት እስከ ወረዳና ገጠር ድረስ እንዲደርስ ታደርጋላችሁ

አዎ አሉን፡፡ ለምሳሌ በግሎባል ፈንድ የሚደገፍ ኬር ኤንድ ሰፖርት ፕሮጀክት በ41 ወረዳ ከ41 ኤች አይቪ ኤዲስ አሶሴሽን ጋር ይሰራሉ፡፡ ኪይ ኤንድ ፕራዮሪቲ ፖፑሌሽ የሚባለው ደግሞ በ36 ከተሞች ላይ ይሰራል፡፡ በእነዚህ ሰላሳ ስድስት ከተሞች ሁለት አይነት አገልግሎቶች አሉ፡፡ ኪይ ኤንድ ፕራዮሪቲ ፖፑሌሽ የሚባሉት ለምሳሌ ሴተኛ አዳሪዎች ነቸው፡፡ ስለዚህ ለእነርሱ አስፈልጋዊን አገልግሎት እንሰጣለን ማለት ነው፡፡

1. ስለዚህ አግለግሎቱ ማዕከል የሚያደርገው ከተማን ነው ማለት ነው

ወረዳ እና ከተማ አስተዳደሮችን ነው ማዕከል የሚያደርገው፡፡ በበጀት እጥረት ምክንያት ሆት ሰፖት የሚባሉ አካባቢዎች ላይ ብቻ ያተኮረ ነው፤ ወደ ገጠሩ ማህበረሰብ ተደራሽ አደለንም፡፡

1. ክትትል የማድረግ ሁኔታ እንዴት ነው ስራዎች በትክክል ስለመሰራታቸው እንዴት ታውቃላችሁ

እንደነገርሁህ ከ200 በላይ ሰራተኛ አለን፡፡ ዋናው መስሪያ ቤት (ሄድ ኦፊስ) ላይ ያለን ወደ 22 የምንሆን ሰራተኛ ነው ያለን፡፡ ሌላው ግን አውት ሪች ሰራተኛ አሉን፡፡ የድጋፍ ክትትል፣ ዶክመንት ሪቪው ማድረግ እና የመሳሰሉት ስራዎችን እንሰራለ፡፡

1. ፍትሓዊ አገልግሎት አሰጣጡን እስኪ በዝርዝር ንገረኝ

በተወሰኑ አካበቢዎች ላይ ብቻ ተወስነን እንደመስራታችን ፍትሓዊ ነው ብየ አላምንም፡፡

1. ሁለት ጊዜ እና ሶስት ጊዜ ተጎጂ የሆኑ የማህበረሰብ ክፍልን ከግምት አስገብቶ አገልግሎቱን ተደራሽ በማድረግ በኩል ምን እየሰራችሁ ነው

እንደዚህ አይነት ግንዛቢ የለንም፡፡ እንደ አጠቃላይ ችግሩ የሚስተዋልበትን አካባቢ ለምሳሌ የተመረጡ ከተሞች ላይ ለጠቅላላው ማህበረሰብ አገልግሎት እንሰጣለን እንጂ በዚህ አንግል ታይቶ የሚታወቅ አይመሰለኝም፡፡

1. ተጨማሪ ፍትሓዊ አገልግሎት ለመስጠት ተግዳሮቶች ካሉ በዝርዝር ብትነግረኝ

አንተም እንዳየኸው መንግስት ዝም ብሎ መልቲ ስክቶራል ሪስፖንስ ያስፈልጋል አለ እንጂ የሚመለከታቸው አካላት ሁሉም በሚገባው ልክ እየሰሩ አይደለም፡፡ለአንድ መስሪያ ቤት ብቻ ግዴታ አድርጎ የመመልከት ሁኔታ አለ፡፡ እና ሁሉም ኮሚትድ አደሉም፡፡ ለምሳሌ እያንዳንዱ መስሪያ ቤት 2 ፐርሰንት እንዲመድብና በየመስሪያ ቤቱ ውይይት እንዲካሄድ መመሪያ ወጥቱዋል፡፡ ነገር ግን ያንን ስንቶቹ ይተገብሩታል ቢባል አንድ እንኩዋን አታገኝም፡፡ ነገር ግን ምንድን ነው የሚያደርጉት… በጀቱ እንዳይመለስ በበጀት መዝጊያ አመት ላይ ተሰባስበው ወደ ሌላ ቦታ ሂደው ስብሰባ አካሂደው አበል ነው የሚያወራርዱት፡፡ ስለዚህ ከላይ እስከ ታች የትኩረት ማነስ እንዳለ፣ ፖሊሲዎቹና ስትራቴጂዎቹ ቢኖሩም የማስፈጸም አቅማችን ውስን እንደሆነ እና እነዚህ እነዚህ ነገሮች አሉ፡፡ ዋናው ግን የትኩረትና የበጀት ማነስ ጉዳዮች ናቸው፡፡

1. ጦርነት አካባቢ ላይ እንዴት ተደራሽ ታደርጋላችሁ

ጦርነት በሚካሄድባቸው አካባቢዎች አንዳንድ ስራዎችን ለመስራት እየሞከርን ነው፡፡ በጣም እሩቅም ስለሆነ፣ ያለን በጀትም ቲንሽ ስለሆነ ፍላጎት ቢኖርም በሚገባው ልክ መድረስ ያዳግታል፡፡

1. የማህበረሰቡ ባህል እና ሴት ስራችሁ ላይ ተግዳሮት ይሆናል

የኤች አይቪ ፕሮግራም አንዱ ተግዳሮት ስቲግማ እና ዲስክሪሚኔሽን ነው፡፡ ለምሳሌ፡ የመንግስት ባለስልጣን፣ ባለሃብቶች ወይም በብዙ ሰዎች የሚታወቁ ሰዎች ቫይረሱ በደማቸው ሲኖር አካባቢያቸው ከሚገኝ ተቁዋማት አይደለም አገልግሎት የሚያገኙ፤ እሩቅ ሂደው ከማይወቁበት ነው አገልግሎት የሚያገኙት፡፡ለምሳሌ ባርዳር ያለ ሰው ደብረታቦር ሂዶ አገልግሎት ያገኛል፡፡ እና የእኛ አካባቢ ባህልና ወግ ጠበቅ ያለ ስለሆነ በዚህ አግባብ ነጻ ሁኖ አገልግሎቱን ለሁሉም ተደራሽ ለማድረግ የሚቸግርበት ሁኔታ ይስተዋላል፡፡ ከሐይማኖት አንጻር ጠበል የሚሄዱ ሰዎች አልፎ አልፎ መድሃኒታቸውን የሚያቁዋርጡ መሆናቸው ሌላው ተግዳሮት ነው፡፡ በእስልምናም ዱዓ አስደርጋለው በማለት መድሃኒት መተው፡፡

1. ወደ ፊት ለሁሉም አገልግሎቲን ተደራሽ ለማድረግ ምን ቢሆን ይሻላል

አንደ አጠቃላይ አቴንሽን በመንግስት በኩል ኖሮት፣ በመንግስትም በጀት በአለም አቀፍም ሐብት ባሉት መንገዶች በእርዳታ ብቻ ተወስነን የተወሰኑ ማህረሰብ ክፍል ብቻ ከመስራት ለሁሉም ተደራሽ የሆነ የማህበረሰብ አገልግሎት ብንሰጥ፡ አንደኛ ፍላጎታችን ነዉ፡፡ ከፖሊሲ እና ከዳይሬክሽ በተጨማሪ መንግስት ትኩረት ቢሰጠው ጥሩ ነው፡፡ ቀጣይነት ያለው አዌርነስ ስራዎችም መሰራት አለባቸው በቀጣይነት፡፡ ብዙዎች ፕሮግራሞች ሲጀመሩ ለምሳሌ ኤች አይ ቪ ኤዲስ መዲሃኒት ሲመጣ የእድሜ ማራዘሚያ የሚል ስያሜ ሲሰጠው ማህበረሰቡ ከዚህ በሁዋላ አልሞትም ወደ ማለት ደረጃ ደረሰ፡፡ እና ከኤች አይ ቪ ይልቅ ደም ግፊት ስኩዋር እና የመሳሰሉትን በሽታዎች ነው የሚፈሩት እና እሱን እንደ ችግር አለማየት ስላለ የአዌርነስ ክሬሽን ስራ ቴለርድ የሆነ ስራ መሰራት አለበት፡፡

1. መጨረሻ የምትለው ነገር ካለህ

ምንም የምጨምረው የለኝም፡፡

**#19**

1. Please tell me your role in this position.

The Amhara region where I am now is called the Coalition of Associations, which people living with HIV established. It is contributing to the prevention and control of HIV at the regional and national level as well as medical services. My part in this is that I am the Program Director. Roles I have the role of making annual plans, directing, organizing. Most of our work is carried out as a national plan, which is implemented at the regional level, and in particular, to achieve the ambitious three-ninety-five goals set by the UNAIDS and to eliminate the threat of AIDS by 2030. For example, HIV testing is widely used.

In addition, plans are structured there to keep our clients on the continuum of care or in treatment. As a Network, it has about three projects with about 221 employees. And we have these workers working together.

1. What are the strategies you follow when providing these services?

Our first one is called the GIPA Principle as a Network. The GIPA Principle stands for Greater Involvement of People with HIV/AIDS. What it means is that in any HIV program planning, implementation, monitoring, and evaluation, the involvement of people with HIV is required.

1. If you think there are activities left undone, please explain to me.

The strategic plan has about six strategic goals. But there is no resource allocation as it used to be. On the other hand, if we look at the Amhara region, the HIV rate of the Amhara region is higher than the national prevalence rate. However, prevention packages are mostly not being implemented. Of course we do prevention. But we are targeted. For example, there are so-called key and priority populations, such as drivers, injecting drug users, young women, those who wear uniforms (መለዮ ለባሾች), and prostitutes. In order to demand for testing, we are looking for index cases, we are looking for the vulnerable people, not the general population. And when we hear from some of the public, even if it is not supported by research, people fear other chronic diseases, like diabetes, hypertension rather than HIV because there is an understanding that it is possible to live long years while taking medicine. And education is still needed. However, when it comes to the HIV testing, not everyone is being tested to know their status. It requires a lot of counseling and training and information packages. And trainings, information packages, I don't think we're getting to where we should be.

1. How is equitable access to HIV/AIDS services like?

Previously there was a single office where HIV programs were being implemented at the government level. The government recognized it as HIV Secretary, and it was working in the same office in the region and zone. But now, when the resource challenge comes and the government's attention is less, now a process exists in the health office as a minor department; It means as a single workflow. ... it is being done by certain people with a limited budget (with limited personnel, with limited budget). It is not being integrated and given that much attention. And because of the lack of budget, now international programs have come to be a targeted services which focus only on specific population.

1. Can you mention some of the reasons why HIV/AIDS services are not equitable?

So, lack of attention is a problem in society and the government. The community is not interested. Resource availability is also a problem. Even if there is a need, for example, when people get married, they do a preliminary examination, but there is no kit. It is the basic resource. On the one hand, they are not finding people who want to do that. Some get married because their education and understanding is not up to date. Therefore, the main reason, as I said earlier, is the lack of human resources, budget problems and especially awareness issues that make it unequitable.

1. At the level of policy and strategy, please explain to me if there are any actions that you are doing to overcome the injustice seen due to source of income, education level, residence.

We encourage inclusiveness. We have four core principles, including inclusiveness and gender equality, but when it comes down to that level, it's not practiced at it is written at strategy level. Especially as our sector is works with the help of non-governmental organizations, it is based on the needs of donors. So, this itself prevents equitable service provision. At the government level, there will probably be no policy situation. But the existing guidelines and policies are not implemented effectively. And I don't think strong work is being done on targeted, for example, sex workers, for women, for vulnerable sections of society.

1. What are the things that can cause this gap to be created?

A commitment problem may be one. But there is also the negligence of society. Lack of need for medical services.

1. Do you make the services that you provide reach to the districts and rural areas?

Yes, we have. For example, they work with 41 HIV-AIDS associations in 41 districts of the Global Fund-supported Care and Sport project. Key and Priority Population also operates in 36 cities. There are two types of services in these thirty-six cities. Key and priority populations are, for example, prostitutes. So, we provide them with the services they need.

1. Is, therefore, the service is centred on the city?

It makes the district and city administrations central. Due to lack of budget, it is only focused on hotspot areas; We are not accessible to the rural community.

1. How do you know if the work is done properly?

As I told you, we have more than 200 employees. We have about 22 employees at the head office. Another thing is that we have an outreach worker. We do support monitoring, document review, and more.

1. Tell me in detail about the equitable service

I don't think it's fair to say that we are limited to certain areas.

1. What are you doing to make the service equitable by considering the twice and thrice victims of society?

We have no such understanding. I don't think it has been seen from this angle, but we provide services to the entire community in areas where the general problem is noticed, for example in selected cities.

1. If there are other challenges to provide equitable services, please tell me in detail.

As you can see, the government simply said that a multi-sectoral response is needed, but the relevant bodies are not all working as they should. And not all are committed. For example, instructions were issued for each office to allocate 2 percent and to hold a discussion in each office. But how many of them will do that…?, you won't get one. But what do they do... They get together in the closing year of the budget and go to another place to hold a meeting and argue about the per diem. So, there is a lack of attention from top to bottom, despite the policies and strategies, our implementation capacity is limited and so on. But the main issues are lack of attention and budget.

14. How do you provide equitable services during the war zone?

We are trying to do some work in war zones. Because it's too far, and our budget is small, it's difficult to reach the right place even if there is demand.

1. Does society's culture and value’s challenging to deliver equitable HIV/AIDS services?

One of the challenges of the HIV program is stigma and discrimination. For example, government officials, investors, or people known by many people who have the virus in their blood, do not receive services from their local hospitals. They go far and get service from unknown places. For example, a person from Bahir Dar goes to Debre Tabor and gets service. And since our area has a strict culture and tradition, it is difficult to make the service accessible to everyone in this manner. Another challenge is that people who go to religious practice sometimes change their medicine. For example, in Orthodox Christian during using Holey water and in Islam, giving up taking medicine by saying that I will make Dua.

1. What would be better to make the service accessible to all in the future?

Give general attention from the government. Rather than limited by the means of the government budget and international resources only, I would say providing community services that are accessible to all by our efforts. Apart from policy and directives, it would be good if the government should pay attention. Continuous awareness activities should also be done continuously. When many programs were started, for example, when the HIV/AIDS drug came out and it was called life extension, society reached the stage of saying that I will not die after this. And instead of HIV, they are afraid of high blood pressure, Diabetes and other diseases, and because they don't see it as a problem, awareness creation work should be done in a tailored way.

17. If you have something to say at the end, please.

I have nothing to add.

**#20**

1. ኃላፊነት እና ስለምትሰሩት፡ ሰራተኛው እራሱንና ቤተሰቡን ከኤች አይቪ እንዲጠብቅ ግንዛቤ መስጠት፣ በራሪ ወረቀት ማዘጋጀትና መበተን፣ ኮንዶም ስርጭት

በተሰዘዋወሪ ጉዳይ አድሎና መገለል አለ፤ ቫይረሱ በደሙ ያለበትን ሰዎች እድገት እንዳያገኙ ይደረጋሉ፡፡ ቅሬታዎች ይቀርባሉ፡፡ ህብረተሰቡም ሆነ አመራሩ በወቅታዊ ሁኔታዎች ስራ ላይ ይጠለፋል እና ኤች አይ ቪ ይረሳል፡፡ ለምሳሌ፡ በኮቪድ ጊዜ ስኮቪድ፣ አሁን ደግሞ ጦርነቱ ይህ ሁሉ መሰናክል ነው፡፡ ሌላው ሰው በአወቀው ልክ እየተለወጠ አይደለም፡፡ ስለዚህ አሁንም የባህሪ ለውጥ እያመጣ አደለም ማለት ነው፡፡

1. ያልተሰሩ ስራዎች ካሉ ብትነግሪኝ

ሰራተኛው ከወር ደሞዙ በፈቃደንነት እንጂ ስርዓት ተዘርግቶለት አይደለም የሚሰራ፡፡ ሌላው በጣም መቀዛቀዝ አለ፡፡

በእኛ በኩል ተደራሽ ያልሆነ የማህረሰብ ክፍል የትኛው ነው ብለን የምንሰራበት አሰራር የለም፡፡ የወል ነው፡፡

1. ፍትሐዊ ተደራሽ ለማደረግ የሚቸግራችሁ ነገር ምንድን ነው

አንደኛ ከጤና ቢሮው ጋር ተቀናጅቶ የመስራቱ ነገር በጣም የተቀዛቀዘ ነው፡፡ ምክንያቱም በየወቅቱ ስለኤች አይ ቪ አዳዲስ ነገሮች ላይደርሱኝ ይችላሉ፡፡ እኔ ጋር ያለው ዶክመንት መመሪያ ነው፤ አንድ ግለሰብ ነኝ፡፡ ተጨማሪ ሰው ያስፈልግሃል፣ ተጨማሪ በጀት ያስፈልግሃል፡፡ ወደ ታች ወርዶ መሰራት ያለባቸው ሁኔታዎችን ለይቶ አለማውጣቱ የኔም ችግር ሁኖ ግን አንደኛ ከጤና ቢሮም ጋር፣ የሚዲያ ሽፋን ከአለመኖርም የተነሳ ሪፖርት መላላክና መመላለስ እንጂ መሬት የነካ ስራ እየሰራን አይደለም፡፡ ወጪ ሳያስወጣ የሚሰሩ ስራዎች እንዳሉ ሁነው ትኩረት አለማድረጉ፣ በጀት ማነስ እና አለመቀናጀት ችግሮች ናቸው፡፡ የሰው ሃይልም እጥረት አለ፡፡ የስርዓተ-ጾታና የኤች አይቪ ጉዳይ አንድ ሰው ነው፤ ስለዚህ ሁለቱን ትልልቅ ጉዳዮች ይቅርና አንደኛውን ብቻ በአንድ ሰው ለመስራት ቀጥ ያለ ዳገት እንደመውጣት ይደክማል፡፡

ፍትሓዊ ተደራሽነት በሚገባው ልክ ተቁዋማዊ ነው ብየ አላምንም፡፡

ማህበረሰቡ በኩል ሌላው ችግር ስለ ኤች አይ ቪ መከላከል ስታወራው እኛ አንድ ለአንድ ነን፣ እንተማመናለን አያስፈልገንም ይሉሃል፤ ነገር ግን ከሁለት አንዳቸው ወደ ሌላ የሚሄዱበት ሁኔታ አለ፡፡

የመልካም አስተዳደር ችግር አንዱ ነው፡፡

1. ወደ ፊት ቢሰራ የምትይው ነገር ካለ

ሁሉም የማህረሰብ ክፍል ላይ መሰራት አለበት፡፡ የተማረውም ያልተማረውም ከልጆች እስከ ታላላቆች ነገሩ ቢራገብ ጥሩ ነው፡፡ መድሃኒት መውሰድ እንደ ትልቅ ነገር በመቁጠር በሽታው አቅልሎ እንዳያዩት ቢደረግ፡፡

1. መጨረሻ ላይ የምትይው ነገር ካለ

ከምርመራ አገልግሎት ጀምሮ ለሁሉም ቢሰጥ፤ ብቻህን ከሆንህ አንመረምርህም የሚባል ነገር አለ እሱ ነገር ቢስተካከል፡፡ አንድ ሰው ብቻውን ሂዶ መመርመር ቢፈልግ የፍቅር ጉዋደና ይዘህ ና ይባላሉ፡፡ ይህ ሰው በመጥጥ ተገፋፍቶ ስፈራ ራሱን ለማወቅ ሲሄድ ሂደህ ፍቅረኛህን ወይም ሚስትህን ይዘህ ና ይባላል፡፡ ምናልባት ራሳቸውን መመርመር እንዲችሉ መመርመሪያ መሳሪያ በየመድሃኒት ቤት ቢቀመጥ ገዝተው መጠቀም የሚችሉ ራሳቸውን ማወቅ ይችሉ ይሆናል፡፡

**#20**

1. Please tell me your responsibilities and what you do.

Providing awareness to the employees to protect themself and their families from HIV, preparing and distributing leaflets, distributing condoms.

There is discrimination and stigmatization in the society and in sectors. People who have the virus in their blood will not be promoted in their career. Complaints came from employees who are living with HIV. Both society and the leadership get caught up in the current situation and HIV is forgotten. For example: during covid, and now the war is all these hindrances. Another thing, individuals are not changing as they know. So, it is still not bringing about behavioral change.

1. Tell me if there are any undone activities.

The employees pay from their monthly salary voluntarily, but not based on a structured system.

We do not have a system to determine which part of the population is inaccessible or accessible. Our activity is public.

1. What is difficult for you to provide equitable services?

First, the coordination with the health office is cooled down. Because I may not receive updated information about HIV. The document I have with me is a guide; I am a single person; you need more people; you need more budget. It is my problem not to identify the gap that needs to be worked on. With the Health Bureau, we only give and take reporting only. Due to the lack of media coverage, we are not doing any ground-breaking work. Lack of attention, lack of budget, and lack of coordination are problems that can be done without spending money. There is also a shortage of manpower. There is only one person for gender and HIV directorate. So, it's as tiring as climbing a steep hill to do just one of them, how can only me effectively work for these two big issues.

I don't think equitable HIV services is institutionalized.

Another problem on the part of the community is that when you talk about HIV prevention, they say that we are one-on-one, we trust each, we don't need your advice; But there is a situation when one of the couples go to another opposite sex.

Another is the problems of good governance.

1. If there is something left you want to suggest being improved in the future, please.

It is good if all the educated and the uneducated, from children to adults take HIV as an agenda, take it as a big health problem and do not see it as a simple problem.

1. If you want to say anything at the end, please.

I would say it is better if services including HIV testing are given to all individuals. There is a problem that clients are told that if you are alone, we will not examine you. If a person wants to be tested alone, they are told to bring his parents or girl/boyfriend. When a person who may be pushed emotionally committed unsafe sex and want to take HIV test alone, but from health institution side, it is said to go and take his lover or wife. Maybe if every drug store has a self-diagnosing device that they can buy and use, they will be able to self-diagnose themselves.

**#21**

1. በኤች አይ ቪ ጉዳይ ያለብህን ኃላፊነት እና የስራ ድርሻ

ተጠሪነቴ ለቢሮው ኃላፊ ሁኘ ኃላፊነቴ የኤች አይ ቪ ኤዲስና ስርዓተ-ጾታ ጉዳይ ባለሙያ ሁኘ እያገለገልሁ ነው፡፡ በዝርዝር የምሰራው ስራ ኤች አይ ቪ ጉዳይ ከጤና ቢሮው ጋር በመነጋገር ነው የምሰራው፡፡ በመስሪያ ቤቱም ውጤት ተኮር በመስሪያ ቤቱ ጋር እናቅዳለን፡፡ ሰራተኛውን ማወያየት፣ ማንዋል፣ ብሮሸር ማዘጋጀት እና መበተን፣ ኮንዶም ማሰራጨት አለ፡፡ ሰልጠና እንሰጣለን፡፡ ሁለት ልጆችን በወር 700 ብር እንሰጣለን፡፡ ሰራተኞችን ወረታ ወስደን በቀን 700 ብር ከፍለን አስልጥነናቸዋል፡፡ ዓለም አቀፍ ኤዲስ ቀን እናከብራለን፡፡

1. ፍትሐዊ አገልግሎት ለመስጠት ምን ታደርጋላችሁ

ፍትሐዊነትና ተደራሽነት ቁልፍ ነገር ነው፡፡ በተለይ አሁን ግን ጦርነቱ ምክንያት መንግስትንም ህብረተሰቡም ተዘናግቷል፡፡ ፍትኃዊና ተደራሽ አገልግሎት ለመስጠት መመርመሪያ ኪት ያስፈልጋል፣ በሰፊው የግንዛቤ ፈጠራ መስራት ያስፈልጋል ልክ እንደ ድሮው፣ በእኛ ጊዜ እኮ ይሰራ የነበረው ስራ፡፡ አሁን እኮ ስራ የለም፡፡ አሁን ስራ የለም፡፡ ተዘናግቷል ሁሉም በእራሱ ችግር፡፡ ለምሳሌ ከእኔ ብጀምር 30 ዓመት አገልግየ ደሞዜ የጀማሪ ሙያተኛ አያክልም፡፡ ማስተርስ ዲግሪ ይዠ ዛሬ መኖር ተቸግሬአለሁ፡፡ ስለዚህ ድህነቱ ነው የበለጠ እያስቸገረው ያለ፡፡ ኢኮኖሚው የተጨናነቀ ነው፡፡ ሰው አሁን የእለት ፍጆታው ላይ እንጂ ስለበሽታው ትኩረት የሚሰጠው የለም፡፡ በፊት ሃብኮ በነበረበት ጊዜ በአገሪቱ ጠቅላላ የማህበረሰብ ንቅናቄ እንፈጥር ነበር፡፡ መዘናጋትና የኢኮኖሚ ችግር ቁልፍ ተግዳሮቶች ናቸው፡፡ በፊት የነበረው የውጭ አገር ድርጅቶች እርዳታ አሁን የለም፡፡ ህብረተሰቡ አሁን ያለው ስኩዋር፣ ደም ግፊት፣ ካንሰር እንጂ ኤች አይ ቪ የለም ወደ ማለት ደርሷል፡፡

1. ተጨማሪ ተግዳሮት ካለ ብትጠቅስልኝ

ትልቁ ችግር ኢኮኖሚው ነው፡፡ እሱ ላይ ቢሰራ ትልቅ መፍሔ ነው፡፡ በእርግጥ 2 ፐርሰንት ከመስሪ ቤቱ ይመደባል፡፡ ኤዲስ ፈንድ ደግሞ እንደፈቃዳቸው ሰራተኞች ገንዘብ በወር ይከፍላሉ፡፡ ይህ በቫይረሱ ወላጆቻቸውን ያጡ ልጆች ይረዳሉ፡፡ ነገር ግን እንደ አጠቃላይ ሕብረተሰቡ ዩኑሮ ውድነትና ጫናው ስላለበት ና ተሰብሰብና ላሰልጥንህ ብትለው አይሰማህም፡፡ ሌላው ያለውን ስራዎች ሁሉ እኔ ላይ ነው የወደቀ፤ እኔ ብቻ ነኝ ይህን ሁሉ የምሰራ፡፡ ከጤና ቢሮ የተሰጠው አቅጣጫ ወደ ፊት ጸረ-ኤች አይ ቪ መድኃኒት ነጻ ባይሆንስ ብለን ማሰብ እንዳለብን ይነገረናል፤ ይህ ጠንክረን እንድንሰራ ነው፡፡ ሌላው ጤና ቢሮ እንደ በፊቱ አድምተው አይሰሩም፡፡ እንደገና ስትራቴጂ አይቀይሩም፤ ተመሳሳይ ነገር ነው፡፡ ድሮም ከሀ እስከ ፐ ነው አሁንም ኩ እስከ ፐ ነው፡፡ አሁን ቴክኖሎጂው ረቂቅ ነው፤ መረጃ በሰከንዶች ነው የሚዘዋወረው፡፡ ዘመኑን የሚመጥን አሰራር መዘርጋት ያስፈልጋል፡፡ የአገር አለመረጋጋት፣ የግብዓት እጥረቶች፣ ቅስቀሳ አለማድረግ፣ በጀት እጥረት፣ እረጂ ድርጅቶች መቀዛቀዝ፣ ጦርነቱ አደናቃፊ ነው፡፡ ለሰራተኞች እድገት አይሰራልንም፡፡ ይህ ደግሞ ተነቃቅተን ስራ እንዳንሰራ ያደርጋል፡፡ ሌላው ግን የኑሮ ውድነቱ ከባድ ነው፡፡ እውነቴን ነው የምልህ ዛሬ እኔ ቁርስ አልበላሁም፡፡ ጤና ቢሮው ፕሮጀክት ቀርጸው ትልልቅ ድርጅቶች ጋር መስራት አለባቸው፡፡

1. ወደ ፊት ይስተካከልና ይሰራ የምትለው ነገር ካለ

አንድም ሰው በኤች አይ ቪ ኤዲስ እንዳያዝ የሚለውን ስትራቴጂ ለማሳካት ጠንክሮ መስራት ያስፈልጋል፡፡

1. ተጨማሪ ሐሳብ የምትነግረኝ ካለ ብትነግረኝ

የለኝም አመሰግናለሁ፡፡

**#21**

1. Please tell me your responsibility and role in the HIV issue

I am responsible for the head of the office, and I am serving as an expert on HIV AIDS and gender issues. My detailed work is dealing with the HIV issue with the health office. We plan with the office based on results. There is staff briefing, manual, brochure preparation and distribution, condom distribution. We provide training. We give two children 700 Birr per month. We hired workers and trained them by paying 700 Birr per day. We celebrate World AIDS Day every year.

1. What do you do to provide equitable services?

Equity and access are key. Especially now, because of the war, both the government and the society are distracted. In order to provide fair and accessible services, diagnostic kits are needed, it is necessary to work on awareness creation widely, just like the work that was done in previous eras. Now there is no work. There is no work now. All distracted by his own problems. For example, if I start with 30 years of service, my salary will not exceed that of a beginner professional. Master's degree I dream of living today. So, it is my poverty that is bothering me more. The economy is tight. People now focus on their daily consumption and not on the disease. Before when there was HAPCO, we used to create a whole community movement in the country. Negligence (መዘናጋት) and economic hardship are key challenges now. The help from foreign organizations is no longer there. The current society has come to say that there is no such HIV, fearless to HIV, but cancer and high blood pressure.

1. If there is any additional challenge, please let me know.

The biggest problem is the economy. It's a big solution if it works on economy. Of course, 2 percent is allocated from each sector. Employees also pay monthly for AIDS fund as they wish. This will help children who have lost their parents due to the virus. But like the whole society, because of its high living cost and economic pressure, they will not listen you if you ask them to gather and train them. All the work that the other has fallen on me. I am the only one doing all this. The direction from the Health Office tells us that we should consider whether anti-HIV drugs are not free in the future; This is for us to work hard. Another problem is health office is not working hardly as pervious time. They don't change strategy again; It's the same thing. It used to be A to Z and now it is again A to Z. Now the technology is emerging. Data is transferred in seconds. It is necessary to develop a system that fits the modern world. The country's instability, lack of resources, lack of mobilization, lack of budget, stagnation of charitable organizations, the war are all our challenges. Sometimes demotivators pull back from doing better. For example, it doesn't work for us carrier development. And this keeps us from being active. For mor than many years, I am on the same career position. Another is the high cost of living. I tell you the truth, I did not eat breakfast today. Another thing the health bureau should design a project and work with large organizations.

4. If there is anything you want to say on solutions in the future, please

Hard work is needed to achieve the strategy of preventing no one from contracting HIV/AIDS.

5. If you have any additional thoughts, please let me know

I don't have more, thanks.

**#22**

1. ኤች አይ ቢ አዲሲን በተመለከተ ያንተ ሓላፊነት

ዜና መስራት፣ አዌርነስ ክሬሽን መንግስታዊ እና መንግስታዊ ካሆኑ ድርጅቶች ጋር ለባለፉት ዘመናት እንሰራ ነበ፡፡ ከቅርብ አመታት በፊት ግን ጤና ቢሮ ከአማራ ሚዲያ ኮርፖሬሽን ጋር በመተባበር ትኩረት የሚባል መርሃ ግብር ሳምንታዊ ብዙ ኤች አይ ቪን በተመለከቱ ጉዳዮች እንሰራ ነበር፡፡ እቅድ በማውጣት ለህብረተሰቡ መረጃ ስናደርስ ነበር፤ እስከ ቅርብ ጊዜ ድረስም ስንሰራ ነበር፡፡

1. ጠቅላላ እውቅናን ከመፍጠር በተጨማሪ በልዩ ሁኔታ ትኩረት በመስጠት የሚሰሩ ስራዎች ነበሩን

አዌርነስ ክሬሽን ስንፈጥር ማህበረሰቡ ሊካላከልባቸው የሚችሉ ነገሮችን አትኩረን እንሰራለን፡፡ ለምሳሌ ሶስት መዎች፣ አራት መዎች የሚባሉ ነገሮች አሉ፡፡ ለምሳሌ አንዱን ሳምንት ስለ ኮንደም አጠቃቀም ልናወራ እንችላለን፡፡ በአመት 360 ቀናት ካሉህ እነዚህን ከፋፍለን እንጠቀማለን፡፡ ሹፌሮች፣ ሴተኛ አዳሪዎች፣ ጨዋታ የበዛት፣ ማንኛውም መንገደኛ ጥንቃቄ እንዲያደርጉ እንሰራለን፡፡ ወቅታዊ ሁኔታዎችን ከግምት ያስገባ አገልግሎት ይሰጣሉ፡፡ በኤች አይ ቪ ከተያዙ ደግሞ እንዴት ከበሽታው ጋር መኖር እንደሚቻል እውቅና ይፈጠራል፡፡በጤናው ዘርፍ ያሉ ሙያተኞችን፣ ሃላፊዎችን እና በበሽታው የሚኖሩ ሰዎችን ሚዲያ ላይ በመጋበዝ መረጃዎችን እናስተላልፋለን፡፡ መረጃውን የሚቀበሉ ሰዎች ሁሉም ቢሆንም ለበሽታው ተጋላጭ ማህበረሰብ ክፍሎችንም ትኩረት እናደርጋለን፡፡ወደ ሴተኛ አዳሪዎችም መረጃ እንጠይቃለን፤ **ከድህነት አንጻር** ለምሳሌ ኮንዶም ሳያደርግ እስከከፈላት ድረስ ግንኙነት እንደሚያደርጉ ይነግሩናል፡፡ ከገጠር የሚመጡ ሰዎችን በተለይ ምርመራ ስለማድረግ ወደ ከተማ በሚመጡበት ጊዜ ሚዲያ ላይ እናቀርባለን፡፡ ወደ ገጠር የኤች ኢይ ቪ ጉዳይ መረጃው እየደረሰ ስለመሆኑ ማወቅ የሚቻለው በተለይ ለጋብቻ የተጫጩ ወጣቶች ወደ ጤና ተቁዋም በመምጣት ለቫይረሱ ምርመራ ያደርጋሉ፡፡ እናም አንዳንድ ጊዜ ከገጠሩ ይልቅ ከተማው ተግባራዊነት ላይም ሆነ በቂ መረጃ ያገኘ ስለመሆኑ ነገሩ ተከርብቶ ታገኘዋለህ፡፡

1. አገልግሎታችሁን ተደራሽ ለማድረግ ለይታችሁ የምትሰሩባቸው ሁኔታዎች ይኖራሉን

ለቫይረሱ ተጠቂ የሆኑ ግለሶብችን ተማሪዎችን፣ ሹፌሮችን፣ ሴተኛ አዳሪዎችን ያማከለ ስራ እንዲሁም ሃላፊዎችን፣ የሃይማኖት አባቶችንና ጤና ሙያተኞችን ሁሉ እናሳትፋለን

ሁለተኛው ተደራሽነት ጂኦግራፊ ተደራሽነት፡ በቴሌቪዥን የሚተላለፍ ፕሮግራም እንደመስራታችን መጠን መብራትና ቴሌቪዥን ካለበት ሁሉ እንደርሳለን ብለን እናምናለን፡፡

ሶስተኛው ተደራሽነት ደግሞ ቫይረሱን ከመከላከል አንጻር በየዞኑ በተራ ልምዶችን እንዲያካፍሉ እደርለን፤ ለምሳሌ በዚህ ሳምንት ማርቆስ ላይ ከሰራን ቀጣይ ደብረታቦር ከዛ ደግሞ ሌሎች ዞኖችን እናሳትለን፡፡

1. ተደራሽነት ላይ ስትሰሩ በፖሊሲ ደረጃ ግንዛቤው አለ

አሚኮ ሬዲዮ ፕሮግራሞች ስላሉት ገጠሩን ማህረሰብ በዚሁ ለመድረስ እየተሞከረ ነው፡፡

ሶሻል ሚዲያውንም አልፎ አልፎ መረጃ እናስተላልፋለን፡፡

1. ይህንን ስራዎች ስትሰሩ ምን አይነት ድጋፍ ታገኛላችሁ

ቫይረሱ በደማቸው ያለ ሰዎችን ራሳቸውን እንዲደጉሙ የማድረግ ስራዎች ይሰራሉ፡፡

1. የእናንተ ስራ የተሳለጠ እንዲሆን ከሰው ሃይል፣ ከፋይናንስ፣ አመራር ድጋፍ ምን ይመስላል

ሚዲያ ስለሆነ በተመደበበለት በጀት ይሰራል፡፡ ትኩረት የሚባል መርሃ ግብር በጤና ቢሮ ድጋፍ የሚሰራ ስራ ነበር፡፡ ከትኩረት መርሃ ግብር በተጨማሪ ምክር ከሃኪምዎ ሚባል የጤና መርሃ ግብር ስላለ ስለ ቫይረሱም ጊዜ ይመደብለታል፡፡ አሁን በምናወራበት ሰዓት ይሄ ፕሮግራም ከቆመ ሁለት ወይም ሶስት ወር ሁኖታል፤ የቆመው በገንዘብ እጥረት ሳይሆን መርሃ ግብሮች በተመቻቸ ሰሌዳ እንዲመሩ፣ ግንዛቤ የሚሰጡ ሰዎችን ባለማመቻቸቱ ምክንያት ትኩረት የሚለው መርሃ ግብር ተቁዋርጡዋል፡፡ በእኛ በራሳችን ለተወሰነ ጊዜ መሄድ ብንችልም ከጤና ቢሮው ቀጣይነት ያለው ክትትል አለመኖር ግን መርሃ ግብሩ መካሄድ አይችልም፡፡

1. ምናልባት ይሄን ነገር ፖሊሲዎች መደራረብ ወይም የአጀንዳዎች ቅደም ተከተል መቀያየር ያመጣው ነገር ይሆን

ገንዘብ ድጋፍ በተጨማሪ በአወረድ እስከ ወረዳ ያሉ አመራሮች ተባባሪ መሆን አለባቸው፤ በእርግጥ ቀደም ሲል የነበረው አይነት ትኩረትና የገንዘብም ድጋፍ ከመንግስታዊም ሆነ መንግስታዊ ካልሆኑ ድርጅቶች ድጋፍ ቀንስዋል፡፡ ስለዚህ ተባባሪ ስላልሆኑ ይህ ፕሮግራም ቆሙዋል፡፡

1. በሶስትና ከዚያ በላይ ተጎጂ የሆኑ ማህረሰብ ክፍልን ተደራሽ ስለማድረግ የምትለኝ ነገር አለህ

ገጠር ስትኖር፣ ድሃ ስትሆን እና ድሃ ከሆንህ መረጃውን የማግኘት እድሉ አናሳ ነው፡፡ የእውቀትና የግንዛቤ ችግር ካልተማረው ላይ ይኖራል፡፡

1. የኤች አይ ቪ አገልግሎትን ተደራሽንትን ከግምት አስገብታችሁ በምትሰሩት ስራ እንደ ተግዳሮት ልትጠቅሰው የምትችለው ጉዳይ አለህ

ባይ ዘ ዌይ ኤ ቢግ ቢግ ፕሮበሌም ብለን ልንጠቅሰው የምንችለው ነገር ምንድን ነው መሰለህ የአወቅሁሽ ናቅሁሽ ነገር ኤች አይቪ ላይ ይታያል ነው ለእኔ፡፡ ትልቁ ተግዳሮት እኔንም ሊጨምር ይችላል በጣም ግራ የሚያጋባ ነው በእውነት ነው የምነግርህ ብዙ ሰው በበሽታው እየተያዘ ግን ንቀነዋል ነው የምልህ፡፡ ከጤና ሙያተኛው እስከ ሴተኛ አዳሪ ትኩረት እንኩዋን ሲቁዋረጥ ምንም የማይመስላቸው.. በሽታው ጋር ተለማመደነው ይሁን፣ አሁን አሁን ሰው ታሞ ስለማናይ ይሆን የለም የሚል ብቻ ውስጣችን ውስጥ ሰፍኑዋል ነው፡፡ ሁሉም ሰው አዌርነስ አለው ብትልም የመዳፈራችን ነገር እና በሽታው እንደሌለ የማሰብ ነገር ነው ያለ፤ ለምን የሚለውን ሌላ ተጨማሪ ጥናት ሊያስፈልገው ይችላል፡፡

ሌላኛው ተግዳሮት በበሽታው ያሉ ሰዎችን እንዲያስተምሩ ስንጋብዝ አለመገኘት ችግር ነው፡፡ ይህ ምክንያት ነጻ ያለመሆንና አድሎና መገለልን ስለሚፈሩ ግንዛቤ ፍጠሩልን ስንላቸው እሺ አይሉም፡፡

1. ተቀናጅተው ከመስራትና ክትትል ከማድረግ አንጻር ሚዲያችሁ በኩል ምን ይመስላል፡፡

አንደኛው መስሪያ ቤት ከሌላኛው ልምድ የመውሰድ ነገር ቢኖርም፤ ቫይረሱ በደማቸው ከሚገኝባቸው ሰዎችን በገንዘብ ከመርዳት ባለፈ ሰፋ ያለ ስራዎች እየተሰሩ አደለም፤ ያው አሁን ትኩረታችን በአገሪቱ ባለችበት ሁኔታ ነው (በሰዓቱ የእርስ በእርስ ጦርነት ወቅት ነው)፡፡ ፕሮግራሞች ለረጅም ጊዜ ሲሰሩ የተደጋገሙ ሲለሚመስሉን የመሰላቸት ነገሮች ይኖራሉ፡፡

በሽታውን ከመከላከል አንጻር ግን ከቀድሞው ይልቅ ኮንዶም ዲስትሪቢውሽን የለም፤ ዋጋውም ውድ እየሆነ ሰዎች ያለኮንዶም ሴክስ እንዲያደርጉ ይጋብዛል፡፡

1. ወደ ፊት የተሻለ ነገር ቢሰራ/ቢሆን የምትለው ነገር ካለ

ጤና ሚኒስቲርና ጤና ቢሮው ትኩረት ቢሰጡት፣ ሜንስትሪም የሚባለው በወሬ ብቻ ሳይሆን ጤና ቢሮው እስከታች ደረስ ማንቀሳቀስ አለበት፣ እስከ ቀበሌ በመውረድ በጤና ኤክስቴንሽን ጭምር፡፡ ሜኒስትሪም ማድረግ ሲባል ልጆችን እያሳደግሁ ነው ከሚለው ተግባርና ዝንባሌ በዘለለ መሰራት አለበት፡፡ መድሃኒቱ እስካልተገኘ ድረስ ፕሮግሞች ቀጣይነት እንዲኖራቸው መሆን አለባቸው፡፡ ስብሰባ በጣም ይበዛል ተግባር ግን የለም፡፡ ሚስጢር አደለም በተለይ የዚህ ሙያተኞች በሙሉ ወደ ስብሰባ ነው የሚሮጡት ስለዚህ ተአማኒነት ያለው ስራ ለመስራት የጤና ቢሮው ሙያተኞች በሚጋበዙበት ቦታ አለመገኘት እና አበል ያለበት ስብሰባ ላይ ማተኮር እንጅ ተግባርን ያገናዘበ አለመሆን

**#22**

1. **Please tell me about your responsibilities regarding HIV/AIDS**

Making news, Awareness Creation has been working with governmental and non-governmental organizations for years. But a few years ago, the health office in collaboration with Amhara Media Corporation had been working on a weekly program called Attention on many issues related to HIV. We were making plans and providing information to the community. We were working until recently.

2. **In addition to creating public awareness activities, please tell me any works that you have given uniquely.**

When we create Awareness Creations, we focus on issues that the community use to prevent HIV. For example, there are labeling or words start with similar letter in Amharic (three , or four መ) and we work interchangeably and flexibly. For example, one week we might work on condom use awareness creation. If you have 360 ​​days in a year, we will divide these and use them. We urge drivers, prostitutes, gamblers, any traveler to be careful from HIV. We provide services that take current conditions into account. If they are infected with HIV (high risk groups), they will be advised how to live with the disease. We invite professionals in the health sector, managers and people living with the disease to the media and transmit information. Although the people who receive the information are all, we also pay attention to the sections of community that are vulnerable to the disease. We also request information from prostitutes; In terms of poverty, for example, they tell us that they will have sex as long as they pay her without wearing a condom. We also invite people from rural areas into the media, especially when they come to the city to undertake HIV testing. It is possible to know whether the information is reaching the rural areas about HIV, by especially young people who are engaged to get married come to the health center and get tested for the virus. And sometimes you will find that the city is far to practicality than the countryside.

3. Will there be situations where you have to work separately to make your service accessible?

We involve students, drivers, prostitutes as well as leaders, religious leaders and health professionals. The second accessibility is geographical accessibility: as we are doing a television program, we believe that we can reach wherever there is electricity and television. The third approach is to share experiences of each zone to other in terms of preventing the virus; For example, if we work in Debre-Markos this week, then we will work on other zones next time.

4. When working on accessibility, is there awareness at the policy/strategy level?

Amico has radio programs and is trying to reach the rural community.

We also use social media sometimes to share HIV-related information.

5. What kind of support do you get when you do this work?

We supports people living with HIV to support their own life.

6. What is the support from human resources, finance, management to make your work easier?

Since it is a media, it works with its the allocated budget. There was a program called Focus/’Tikuret’/ that supported by the Health Bureau. In addition to the focus/Tikuret/ program, there is a health program called ‘Counseling from your Doctor’ to use our media as information transmission with a scheduled program weekly. As of now, this program has been stopped for two or three months; It stopped not because of lack of money, but because of not facilitating the people who provide awareness on media program. Although we can go on our own for a certain period of time, without continuous monitoring and support from the health office, the program cannot be carried out.

7. Is this may be caused by overlapping policies or changing the order of agendas? In addition to financial support, leaders from the district must be cooperative and supportive; In fact, the previous attention and financial support from both governmental and non-governmental organizations has decreased. Therefore, this program has stopped because they are not supportive.

8. Do you have something to tell me about making accessible to three or more vulnerable groups?

When you live in a rural area, you are poor, and you are less likely to have access to the information. The problem of knowledge and understanding is common in the uneducated.

9. Do you have an issue that you can mention as a challenge in your work considering access to HIV services? By the way, a big problem that I can mention is that it has been seen the proverb in HIV that ‘**I knew you and I unvalue you’**. The biggest challenge can include me too. It's very confusing. I'm telling you the truth. Many people are getting infected, but we ignore it. From the health professional to the prostitute, they don't mind when the weekly program Focus/’Tikuret’/ stopped. We have become accustomed to the disease, but now we don't see anyone sick, so it's just that the virus is not there. Even if you say that everyone has awareness, it is something that we care about and think that the disease does not exist. It may require further research as to why. Another challenge is the lack of awareness creators when we invite people with the disease to teach on media. This is because they are afraid of not being free and being discriminated against, so they don't agree when we invite them to create awareness.

10. What does your media look like in terms of coordination and monitoring?

Even if there is experience share between sectors; Apart from financially helping people with the virus in their blood, there are no extensive works being done. Right now, our focus is on the situation in the country (it is a time of civil war). When programs run for a long time and seem repetitive, things tend to get boring. In terms of preventing the disease, there is no more condom distribution than before; And the price is getting more expensive, inviting people to have sex without a condom.

11.Is there anything you would like to suggest doing better in the future?

If the Ministry of Health and the Health Bureau pay attention to it, HIV mainstreaming is not only a rumor, but the health Bureau should move it all the way down, even through health extension. Mainstreaming concept should be beyond the activities and attitude of only raising children. Programs should be continued because the disease is persisted. There are too many meetings but no action. It is not secret, especially those who are working on HIV/AIDS are run to meeting or training if there is per diem. Otherwise, you could not get them if you invite them to teach the community or allow opportunity to media press without per diem.
